# Age, sex, disease severity, and disease duration difference in placebo response: implications from a meta-analysis of diabetes mellitus

**DOI:** 10.1186/s12916-020-01787-4

**Published:** 2020-11-16

**Authors:** Chu Lin, Xiaoling Cai, Wenjia Yang, Fang Lv, Lin Nie, Linong Ji

**Affiliations:** 1 Department of Endocrinology and Metabolism, Peking University People’s Hospital, No.11 Xizhimen South Street, Xicheng District, Beijing, 100044 China; 2 Department of Endocrinology and Metabolism, Beijing Airport Hospital, Beijing, China

**Keywords:** Placebo response, Type 1 diabetes mellitus, Type 2 diabetes mellitus, HbA1c, Weight

## Abstract

**Background:**

The placebo response in patients with diabetes mellitus is very common. A systematic evaluation needs to be updated with the current evidence about the placebo response in diabetes mellitus and the associated factors in clinical trials of anti-diabetic medicine.

**Methods:**

Literature research was conducted in Medline, Embase, the Cochrane Central Register of Controlled Trials, and *ClinicalTrials.gov* for studies published between the date of inception and June 2019. Randomized placebo-controlled trials conducted in type 1and type 2 diabetes mellitus (T1DM/T2DM) were included. Random-effects model and meta-regression analysis were accordingly used. This meta-analysis was registered in PROSPERO as CRD42014009373.

**Results:**

Significantly weight elevation (effect size (ES) = 0.33 kg, 95% CI, 0.03 to 0.61 kg) was observed in patients with placebo treatments in T1DM subgroup while significantly HbA1c reduction (ES = − 0.12%, 95% CI, − 0.16 to − 0.07%) and weight reduction (ES = − 0.40 kg, 95% CI, − 0.50 to − 0.29 kg) were observed in patients with placebo treatments in T2DM subgroup. Greater HbA1c reduction was observed in patients with injectable placebo treatments (ES = − 0.22%, 95% CI, − 0.32 to − 0.11%) versus oral types (ES = − 0.09%, 95% CI, − 0.14 to − 0.04%) in T2DM (*P* = 0.03). Older age (*β* = − 0.01, 95% CI, − 0.02 to − 0.01, *P* < 0.01) and longer diabetes duration (*β* = − 0.02, 95% CI, − 0.03 to − 0.21 × 10^−2^, *P* = 0.03) was significantly associated with more HbA1c reduction by placebo in T1DM. However, younger age (*β* = 0.02, 95% CI, 0.01 to 0.03, *P* = 0.01), lower male percentage (*β* = 0.01, 95% CI, 0.22 × 10^−2^, 0.01, *P* < 0.01), higher baseline BMI (*β* = − 0.02, 95% CI, − 0.04 to − 0.26 × 10^−2^, *P* = 0.02), and higher baseline HbA1c (*β* = − 0.09, 95% CI, − 0.16 to − 0.01, *P* = 0.02) were significantly associated with more HbA1c reduction by placebo in T2DM. Shorter diabetes duration (*β* = 0.06, 95% CI, 0.06 to 0.10, *P* < 0.01) was significantly associated with more weight reduction by placebo in T2DM. However, the associations between baseline BMI, baseline HbA1c, and placebo response were insignificant after the adjusted analyses.

**Conclusion:**

The placebo response in diabetes mellitus was systematically outlined. Age, sex, disease severity (indirectly reflected by baseline BMI and baseline HbA1c), and disease duration were associated with placebo response in diabetes mellitus. The association between baseline BMI, baseline HbA1c, and placebo response may be the result of regression to the mean.

## Background

A placebo is defined as any therapy that is used for its non-specific psychological and physiologic effect, but has no demonstrated pharmacological effect on the condition being treated [1]. Pharmaceutical and clinical studies have used placebos as a methodological tool to avoid bias, and also to evaluate the pure therapeutic effect of interventional therapies, aiming to avoid introducing ineffective or even harmful treatments into clinical practice [1–4]. Placebo effect is even promoted as a potential treatment modality in medicine [1–3].

According to the expert consensus statement in placebo research field, placebo response, defined as any health change after the administration of placebo, including natural history of a disease or fluctuation of symptoms, response biases, effects of co-interventions, or statistical regression to the mean, has been widely observed in many clinical trials [5]. In fact, an increase in the magnitude of placebo response without significant change in the efficacy outcomes with respect to effect size and success rate is an emerging pattern over time in trials of many conditions, such as depression [6], epilepsy [7], and also diabetes mellitus (DM) [8]. A placebo has been widely used in clinical research in both type 1 diabetes mellitus (T1DM) and type 2 diabetes mellitus (T2DM). For example, the results of the new treatment were compared with placebo to prove the effect on glucose and body weight control. The placebo response was also used to estimate sample size in placebo-controlled trials and to evaluate the true effect size in active-controlled studies or real-world observational studies without a placebo group.

As for T2DM, the placebo response was previously reported by Khan et al. by analyzing the data from the U.S. Food and Drug Administration approved between 1999 and 2015 [8]. However, considering the increasing magnitude of placebo response and expanding clinical trial data, placebo response in T2DM, in terms of both glycemic and weight control, needs to be further evaluated and updated. Placebo response in T1DM has still not been systematically evaluated. Moreover, the potential factors associated with placebo response that might include patient age, gender, disease duration, follow-up duration, and ways of medication delivery have not been fully investigated and delineated. Knowledge from such investigations might suggest potential predictors for placebo responders and inform more precise clinical study design that allow more efficient efficacy evaluation of medical intervention in subpopulation of diabetes.

Since nonpharmacological interventions including behavior modification and nutritional therapies also play a role in glucose control in diabetes management, placebo response and its features mediated by psychosocial context may be more likely to be revealed in DM, which makes it an ideal model to explore placebo response. Therefore, we utilized the data of randomized controlled trials (RCTs) in both T1DM and T2DM and performed a meta-analysis to comprehensively outline the placebo response in DM. We also conducted the analysis for associated factors to propose a featured placebo response pattern associated with patient-level and study-level characteristics.

## Methods

### Data sources and searches

A systemic literature search was conducted for the published studies in the following databases: Medline, Embase, the Cochrane Central Register of Controlled Trials (CENTRAL), and *ClinicalTrials.gov* website, from the inception of each databases until June 2019. A search strategy was performed by using the following terms: metformin (MET); sulfonylurea (SU); alpha glucosidase inhibitor (AGI); thiazolidinedione (TZD); pramlintide; dipeptidyl peptidase-4 (DPP-4) inhibitor; sodium-glucose cotransporter 2 (SGLT2) inhibitor; glucagon-like peptide*-*1 receptor agonist (GLP-1RA); T1DM; T2DM; placebo-controlled; RCTs; cardiovascular outcome and renal outcome. Searches were firstly performed in January 2014 and subsequently updated in December 2017 and June 2019. This meta-analysis was registered in PROSPERO as CRD42014009373.

### Study selection

Studies were included in this meta-analysis if following criteria were met: (1) placebo-controlled, randomized trials; (2) trials in patients with T1DM and T2DM; and (3) trials with HbA1c level and body weight measured in both placebo and active treatment groups. The exclusion criteria were as follows: (1) trials in patients with gestational diabetes or pre-diabetes; (2) trials in which the anti-diabetic treatment in the placebo group could be changed during the period of study; (3) trials with active agent control; and (4) trials with cross-over study design.

### Data extraction and quality assessment

Two investigators independently extracted data by using a standardized form. Information including author, publication year, patient age, and male percentage was obtained from each trial for both placebo and active treatment groups. Trials with placebo agents administered by oral route were grouped for oral placebo response, and trials with placebo agents administered by injection route were grouped for injectable placebo response. Any disagreement would be resolved by the discussion between two investigators under the supervision of a third independent investigator. By using the Cochrane instrument [9], we evaluated the quality of each RCT.

### Data synthesis and analysis

Placebo response were evaluated as pooled effect size (ES) with 95% confidence intervals (CIs) of HbA1c and weight changes from baseline in placebo treatment group by synthesizing mean value with standard error. Higgins *I*^2^ statistics were used to evaluate the between-study heterogeneity. Random-effects model was used in the meta-analysis. Subgroup comparisons for pooled ES were performed within the framework of a meta-analysis. The associations of continuous variables with placebo response on HbA1c and weight changes, including age, male percentage, baseline BMI, baseline HbA1c, diabetes duration, and study duration, were calculated by meta-regression analysis. Since publication year is a discontinuous variable, the associations between it and placebo response on HbA1c as well as weight change were analyzed by ANOVA tendency test. Indirect comparisons between HbA1c reduction and elevation groups as well as between weight reduction and elevation groups were conducted by Mann-Whitney test. The results of indirect comparisons were expressed as median value with accompanying interquartile range (Q1, Q3).

Publication bias was evaluated by Egger’s test, with a *P* value > 0.05 indicating low risk of publication bias. Pool ES analyses and subgroup comparisons were conducted by Review Manager statistical software package (version 5.3, Nordic Cochrane Centre, Copenhagen, Denmark). Meta-regression analysis was performed by the STATA statistical software package (Version 11.0). Mann-Whitney test and ANOVA tendency analysis were conducted by SPSS software (SPSS 24.0. Armonk, NY: IBM Corp). We conducted this study according to the Preferred Reporting Items for Systematic Reviews and Meta-Analyses (PRISMA) guidelines for conducting and reporting meta-analyses of RCTs.

## Results

### Overall characteristics

In all, 432 placebo-controlled clinical trials conducted in patients with DM were included, among which 41 trials were in T1DM with 4328 participants and 391 trials were in T2DM with 33,987 participants (Additional file 1: Figure S1). The baseline characteristics of enrolled RCTs were summarized in detail for T1DM (Additional file 1: Table S1) [10–59] and T2DM (Additional file 1: Table S2) [60–450] respectively. The risk of bias was evaluated by the Cochrane instrument. In T1DM, there were 5 RCTs with high risk and 18 RCTs with uncertain risk of selection bias for random sequence generation, 4 RCTs with high risk and 15 RCTs with uncertain risk of selection bias for allocation concealment, and 5 RCTs with high risk of attrition bias (Additional file 1: Table S3). In T2DM, there were 8 RCTs with high risk and 15 RCTs with uncertain risk of selection bias for random sequence generation, 5 RCTs with high risk and 21 RCTs with uncertain risk of selection bias for allocation concealment, 22 RCTs with uncertain risk of performance bias, 13 RCTs with uncertain risk of detection bias, 37 RCTs with high risk and 30 RCTs with uncertain risk of attrition bias, and 12 RCTs with uncertain risk of reporting bias (Additional file 1: Table S4). The publication bias was accessed by Egger’s test in T1DM (*t* = − 0.29, *P* = 0.771) (Additional file 1: Figure S2) and T2DM (*t* = − 1.34, *P* = 0.179) (Additional file 1: Figure S3) respectively, which both turned out to be insignificant.

### Overall placebo response

**Fig. 1:**
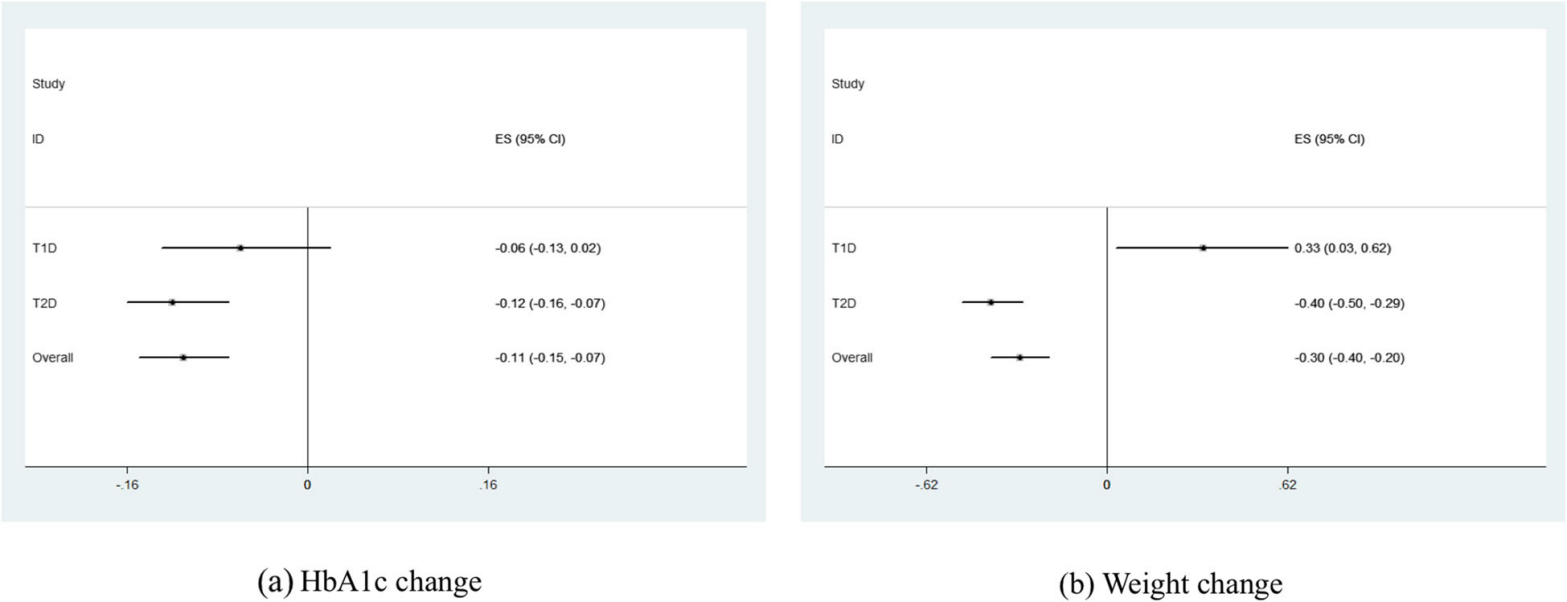
Placebo response stratified by types of diabetes mellitus. **a** HbA1c change. **b** Weight change

**Fig. 2:**
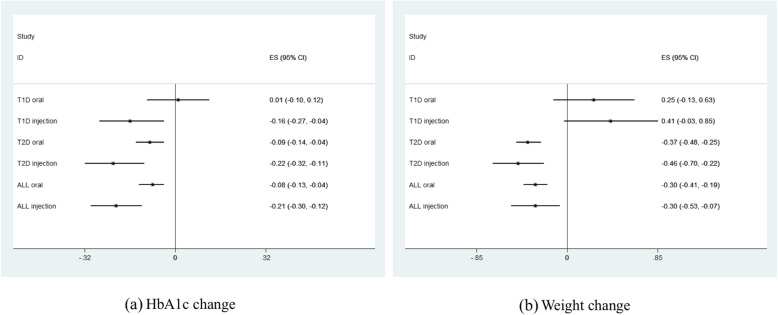
Placebo response stratified by administration routes. **a** HbA1c change. **b** Weight change

According to the meta-analysis, placebo treatment was generally associated with significantly HbA1c reduction (ES = − 0.11%, 95% CI, − 0.15 to − 0.07%) and weight reduction (ES = − 0.30 kg, 95% CI, − 0.40 to − 0.20 kg) in patients with DM (Table 1). As for specific DM types, significantly weight elevation (ES = 0.33 kg, 95% CI, 0.03 to 0.61 kg) was observed in patients with placebo treatments in T1DM subgroup while significantly HbA1c reduction (ES = − 0.12%, 95% CI, − 0.16 to − 0.07%) and weight reduction (ES = − 0.40 kg, 95% CI, − 0.50 to − 0.29 kg) were observed in patients with placebo treatments in T2DM subgroup (Fig. 1). As for treatment design, significantly HbA1c elevation (ES = 0.08%, 95% CI, 0.01 to 0.16%) was shown in monotherapy subgroup while add-on therapy was significantly associated with HbA1c reduction (ES = − 0.20%, 95% CI, − 0.25 to − 0.15%). Furthermore, significantly weight reduction was observed in both monotherapy (ES = − 0.56 kg, 95% CI, − 0.73 to − 0.39 kg) and add-on therapy (ES = − 0.19 kg, 95% CI, − 0.32 to − 0.07 kg) subgroups (Table 1). However, no significant difference was revealed in placebo response from different treatment designs by subgroup comparisons in terms of HbA1c and weight change. In T1DM, significantly HbA1c reduction was observed in patients with injectable placebo treatment (Fig. 2). In T2DM, significantly HbA1c reduction and weight reduction were observed in both oral and injectable placebo treatment subgroups (Fig. 2). Moreover, the magnitude of HbA1c reduction was greater in patients with injectable placebo (ES = − 0.22%, 95% CI, − 0.32 to − 0.11%) treatment than oral ones (ES = − 0.09%, 95% CI, − 0.14 to − 0.04%, *P* for subgroup comparison = 0.03) (Table 1).

**Table 1:** Pooled estimated effect of placebo response in patients with diabetes mellitus

Variables	No. of trials	HbA1c change (%)				Weight change (kg)			
		Pooled ES	95% CI	*P* value	*I*^2^ (%)	Pooled ES	95% CI	*P* value	*I*^2^ (%)
Types of diabetes mellitus									
Diabetes in total	433	− 0.11	− 0.15, − 0.07	**< 0.01**	88	− 0.30	− 0.40, − 0.20	**< 0.01**	71
Type 1 diabetes	41	− 0.06	− 0.13, 0.02	0.16	74	0.33	0.03, 0.62	**0.03**	70
Type 2 diabetes	392	− 0.12	− 0.16, − 0.07	**< 0.01**	89	− 0.40	− 0.50, − 0.29	**< 0.01**	67
Treatment design									
Monotherapy	148	0.08	0.01, 0.16	**0.02**	79	− 0.56	− 0.73, − 0.39	**< 0.01**	63
Add-on therapy	285	− 0.20	− 0.25, − 0.15	**< 0.01**	89	− 0.19	− 0.32, − 0.07	**< 0.01**	71
Administration route									
Oral total	355	− 0.08	− 0.13, − 0.04	**< 0.01**	88	− 0.30	− 0.41, − 0.19	**< 0.01**	61
Injection total	78	− 0.21	− 0.30, − 0.12	**< 0.01**	88	− 0.30	− 0.53, − 0.07	**0.01**	84
Type 1 diabetes									
Oral	31	0.01	− 0.10, 0.12	0.84	73	0.25	− 0.13, 0.63	0.20	63
Injection	10	− 0.16	− 0.27, − 0.04	**< 0.01**	75	0.41	− 0.03, 0.85	0.07	77
Type 2 diabetes									
Oral	324	− 0.09	− 0.14, − 0.04	**< 0.01**	89	− 0.37	− 0.48, − 0.25	**< 0.01**	58
Injection	68	− 0.22*	− 0.32, − 0.11	**< 0.01**	88	− 0.46	− 0.70, − 0.22	**< 0.01**	82
Comparator agent type									
Type 1 diabetes									
Metformin	13	0.21^†^	− 0.06, 0.48	0.13	72	0.71	− 0.29, 1.70	0.17	37
AGI	2	0.01	− 0.36, 0.38	0.97	82	− 0.63	− 2.10, 0.84	0.40	94
TZD	4	− 0.60	− 1.37, 0.18	0.13	0	1.21	− 6.52, 8.94	0.76	0
DPP-4i	1	− 0.12	− 1.59, 1.35	0.87	NA	0.02	− 5.21, 5.25	0.99	NA
GLP-1RA	5	− 0.26	− 0.44 -0.07	**< 0.01**	56	0.41	− 0.27, 1.08	0.24	45
SGLT2i	11	− 0.09	− 0.20, 0.02	0.10	75	0.39	0.08, 0.70	**0.01**	36
Pramlintide	5	− 0.09	− 0.23, 0.05	0.20	79	0.41	− 0.17, 0.99	0.17	85
Type 2 diabetes									
Metformin	23	0.14^†‡^	− 0.05, 0.33	0.14	59	− 0.57	− 0.93, − 0.21	**< 0.01**	0
AGI	40	− 0.01	− 0.24, 0.22	0.91	89	− 0.17	− 0.51, 0.16	0.31	0
TZD	64	0.07^†‡^	− 0.09, 0.24	0.39	76	− 0.17	− 0.66, 0.32	0.50	35
SU	24	0.05^‡^	− 0.19, 0.29	0.68	70	− 0.48	− 1.96, 1.00	0.53	78
DPP-4i	95	− 0.11^‡^	− 0.20, − 0.02	**0.01**	92	− 0.32	− 0.49, − 0.14	**< 0.01**	67
GLP-1RA	68	− 0.22	− 0.32, − 0.11	**< 0.01**	88	− 0.46	− 0.70, − 0.22	**< 0.01**	82
SGLT2i	78	− 0.26	− 0.35, − 0.17	**< 0.01**	90	− 0.42	− 0.60, − 0.24	**< 0.01**	60

Font bold was used if *P* < 0.05 was noticed

*ES* effect size, *SU* sulfonylurea, *AGI* alpha glucosidase inhibitor, *TZD* thiazolidinedione, *DPP-4* dipeptidyl peptidase-4, *SGLT2* sodium-glucose cotransporter 2, *GLP-1RA* glucagon-like peptide-1 receptor agonist

*Compared with oral subgroup, *P* < 0.05

^†^Compared with GLP-1RA group, *P* < 0.05

^‡^Compared with SGLT2i group, *P* < 0.05

### Placebo response stratified by hypoglycemic comparators

**Fig. 3:**
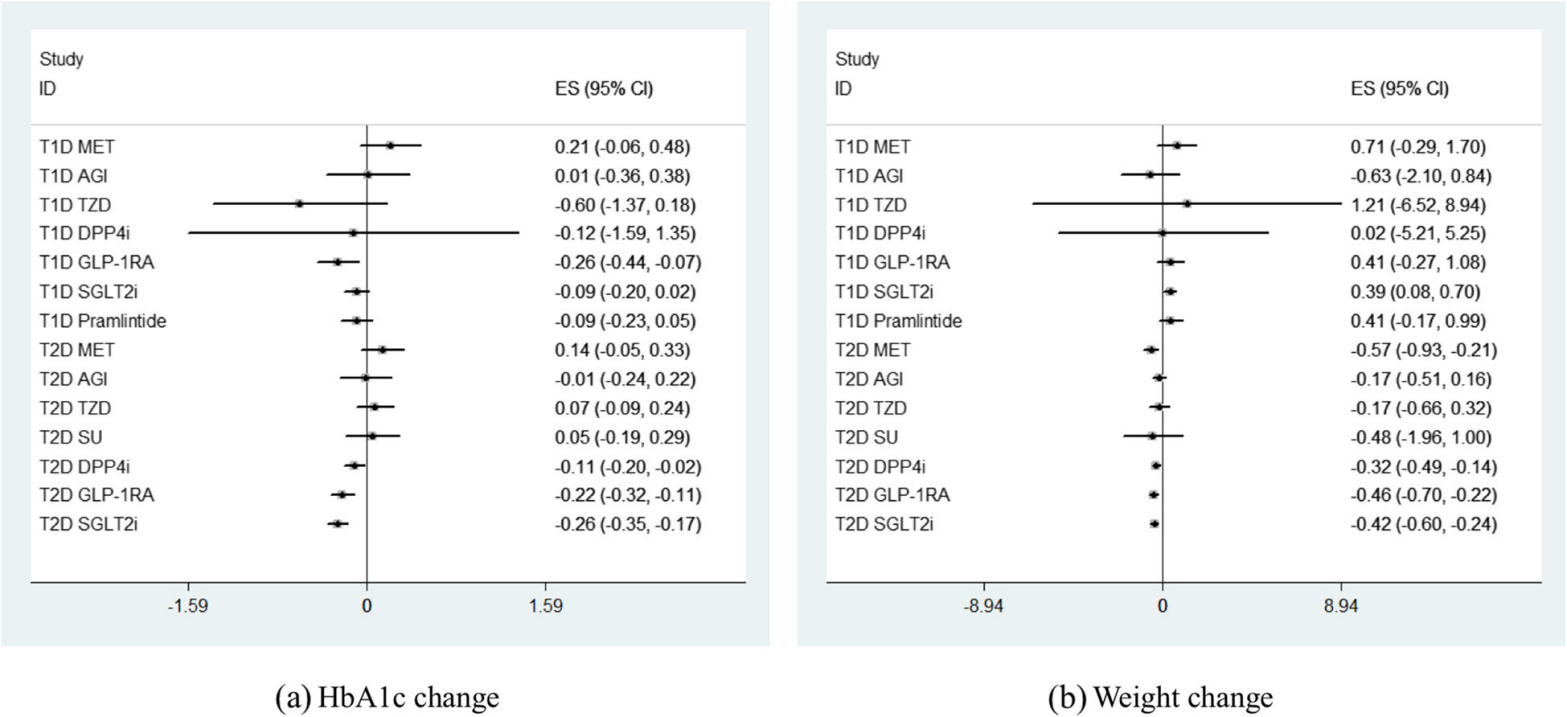
Placebo response stratified by types of hypoglycemic agents. **a** HbA1c change. **b** Weight change

Placebo responses stratified by hypoglycemic comparators were also systematically summarized in Table 1. In T1DM, generally distinct degree of HbA1c reductions was observed in different active comparator subgroups except metformin and AGI subgroups (Fig. 3). Comparable weight elevations were observed in most subgroups except a mild weight reduction seen in AGI subgroup (Fig. 3). In T2DM, placebo in metformin, TZD, and SU subgroups conferred HbA1c elevation effect while HbA1c reductions were observed in AGI, SGLT2i, DPP-4i, and GLP-1RA subgroups (Fig. 3). Weight reductions were shown in all hypoglycemic agent subgroups in T2DM (Fig. 3). By subgroup comparisons, greater HbA1c reduction was observed in GLP-1RA subgroup versus metformin subgroup in T1DM (*P* = 0.01). In terms of T2DM, greater HbA1c reduction was observed in GLP-1RA subgroup versus metformin subgroup (*P* < 0.01) and TZD subgroup (*P* < 0.01) respectively (Table 1). Similarly, greater HbA1c reduction was observed in SGLT2i subgroup versus metformin subgroup (*P* < 0.01), TZD subgroup (*P* < 0.01), SU (*P* = 0.02) subgroup, and DPP-4i subgroup (*P* = 0.02) respectively (Table 1).

### Associated factors with placebo response

#### Age

**Fig. 4:**
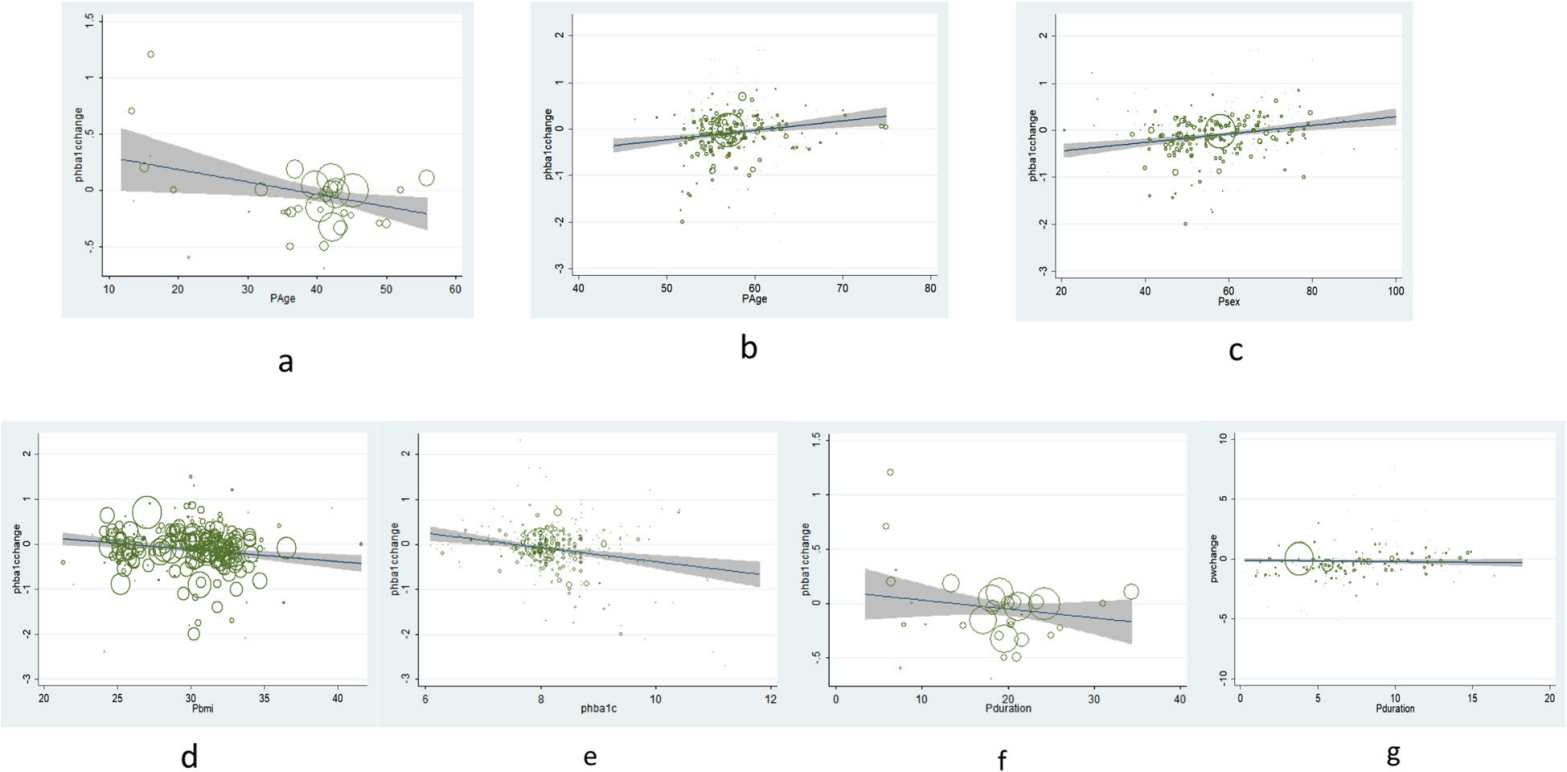
Meta-regression analysis for factors associated with placebo response in diabetes mellitus. **a** Association between age and placebo response on HbA1c change in type 1 diabetes (*β* = − 0.01, 95% CI, − 0.02 to − 0.01, *P* < 0.01). **b** Association between age and placebo response on HbA1c change in type 2 diabetes (*β* = 0.02, 95% CI, 0.01 to 0.03, *P* < 0.01). **c** Association between male percentage and placebo response on HbA1c change in type 2 diabetes (*β* = 0.01, 95% CI, 0.22 × 10^−2^ to 0.01, *P* < 0.01). **d** Association between baseline BMI and placebo response on HbA1c change in type 2 diabetes (*β* = − 0.02, 95% CI, − 0.04 to − 0.26 × 10^−2^, *P* = 0.02). **e** Association between baseline HbA1c and placebo response on HbA1c change in type 2 diabetes (*β* = − 0.09, 95% CI, − 0.16 to − 0.01, *P* = 0.02). **f** Association between diabetes duration and placebo response on HbA1c change in type 1 diabetes (*β* = − 0.02, 95% CI, − 0.03 to − 0.21 × 10^−2^, *P* = 0.03). **g** Association between diabetes duration and placebo response on weight change in type 2 diabetes (*β* = 0.06, 95% CI, 0.02 to 0.10, *P* < 0.01)

The HbA1c level with placebo treatments modestly increased in patients with T1DM below 18 years old (Additional file 1: Figure S4). However, this effect shifted into HbA1c reduction when the patients’ ages became older. Moreover, meta-regression analysis suggested older age was significantly associated with greater HbA1c reduction (*β* = − 0.01, 95% CI, − 0.02 to − 0.01, *P* < 0.01) (Fig. 4a). As for body weight, a profound increase with placebo treatments was found in T1DM patients under 18 (Additional file 1: Figure S4), but no specific changing tendency was observed later (Table 2).

**Table 2:** Meta-regression analysis and tendency test of placebo response with associated factors in patients with diabetes mellitus

Variable	HbA1c change			Weight change		
Analysis for type 1 diabetes						
Multivariate meta-regression analysis						
	*β*	95% CI	*P* value	*β*	95% CI	*P* value
Age (year)	− 0.01	− 0.02, − 0.01	**< 0.01**	− 0.04	− 0.09, 0.01	0.09
Male percentage (%)	0.04 × 10^−2^	− 0.09 × 10^−2^, 0.99 × 10^−2^	0.93	− 0.03	− 0.06, 0.49 × 10^−2^	0.09
Duration of DM (year)	− 0.02	− 0.03, − 0.21 × 10^−2^	**0.03**	− 0.01	− 0.07, 0.05	0.71
Study duration (week)	0.18 × 10^−2^	− 0.14 × 10^−2^, 0.50	0.27	− 0.26 × 10^−2^	− 0.01, 0.83 × 10^−2^	0.63
Baseline HbA1c (%)	0.04	− 0.10, 0.18	0.57	− 0.18	− 0.68, 0.31	0.45
Adjusted HbA1c (%)*	− 0.09	− 0.19, 0.01	0.07	− 0.26	− 0.67, 0.15	0.20
Baseline BMI (kg/m^2^)	− 0.01	− 0.05, 0.03	0.57	0.08	− 0.07, 0.23	0.30
*Adjusted BMI (kg/m*^*2*^*)*^†^	− 0.03	− 0.09, 0.02	0.19	− 0.01	− 0.22, 0.20	0.92
ANOVA tendency analysis						
	Linear term	*F* value	*P* value	Linear term	*F* value	*P* value
Publication year	0.03	0.28	0.60	1.89	3.00	0.09
Analysis for type 2 diabetes						
Multivariate meta-regression analysis						
	*β*	95% CI	*P* value	*β*	95% CI	*P* value
Age (year)	0.02	0.01, 0.03	**0.01**	0.02	− 0.01, 0.05	0.22
Male percentage (%)	0.01	0.22 × 10^−2^, 0.01	**< 0.01**	0.21 × 10^−2^	− 0.01, 0.01	0.71
Duration of DM (year)	− 0.01	− 0.02, 0.01	0.30	0.06	0.02, 0.10	**< 0.01**
Study duration (week)	0.10 × 10^−3^	− 0.23 × 10^−2^, 0.25 × 10^−2^	0.94	− 0.05 × 10^−2^	− 0.01, 0.01	0.86
Baseline HbA1c (%)	− 0.09	− 0.16, − 0.01	**0.02**	0.13	− 0.10, 0.35	0.28
Adjusted HbA1c (%)*	− 0.04	− 0.16, 0.08	0.52	0.10	− 0.23, 0.44	0.54
Baseline BMI (kg/m^2^)	− 0.02	− 0.04, − 0.26 × 10^−2^	**0.02**	− 0.03	− 0.07, 0.01	0.14
*Adjusted BMI (kg/m*^*2*^*)*^†^	− 0.01	− 0.03, 0.01	0.41	− 0.01	− 0.06, 0.03	0.51
ANOVA tendency analysis						
	Linear term	*F* value	*P* value	Linear term	*F* value	*P* value
Publication year	0.85	3.21	0.07	6.85	3.31	0.07

Font bold was used if *P* < 0.05 was noticed

*BMI* body mass index, *DM* diabetes, mellitus

*Adjusted by age, male percentage, duration of diabetes, study duration and baseline BMI

^†^Adjusted by age, male percentage, duration of diabetes, study duration and baseline HbA1c

Unlike T1DM, a profound HbA1c reduction with placebo treatments was observed in T2DM below 50 years old. Such effect grew milder along with growing ages and almost diminished in elderly over 60 (Additional file 1: Figure S5). With significantly younger patient ages in HbA1c reduction subgroup versus HbA1c elevation subgroup (Additional file 1: Table S5), meta-regression analysis suggested younger age was associated with more HbA1c reduction (*β* = 0.02, 95% CI, 0.01 to 0.03, *P* = 0.01) (Fig. 4b). Different degree reductions of body weight were shown in different age subgroups (Additional file 1: Figure S5) but no significant association was discovered.

#### Sex

In T2DM, HbA1c reduction with placebo treatment was more frequently observed in groups with lower male percentages (Additional file 1: Figure S6) and the male percentages in HbA1c reduction subgroup were significantly lower than those in HbA1c elevation subgroup (Additional file 1: Table S5). Meta-regression analysis consistently showed that a lower male percentage was significantly associated with more HbA1c reduction by placebo in T2DM (*β* = 0.01, 95% CI, 0.22 × 10^−2^ to 0.01, *P* < 0.01) (Fig. 4c). Although weight reduction was displayed in subgroups stratified by male percentage in T2DM (Additional file 1: Figure S6), no significant association was shown by meta-regression. Moreover, no significant association was found between male percentage and placebo response on HbA1c or weight change in T1DM (Additional file 1: Figure S7).

#### Baseline BMI

In T2DM, the HbA1c level with placebo treatment was decreased in patients with baseline BMI over 25 kg/m^2^ (Additional file 1: Figure S8). Accordingly, baseline BMI was significantly higher in patients with HbA1c reduction versus HbA1c elevation (Additional file 1: Table S5). Meta-regression further confirmed that higher baseline BMI was significantly associated with more HbA1c reduction (*β* = − 0.02, 95% CI, − 0.04 to − 0.26 × 10^−2^, *P* = 0.02) (Fig. 4d). However, the association turned to be negative after adjusted by age, male percentage, duration of diabetes, study duration, and baseline HbA1c (*β* = − 0.01, 95% CI, − 0.03 to 0.01, *P* = 0.41). Weight reductions were observed in different BMI strata with placebo treatments except for the subgroup over 35 kg/m^2^ (Additional file 1: Figure S8). Although baseline BMI was significantly lower in patients with weight reduction versus weight elevation (Additional file 1: Table S5), no specific response pattern was found for baseline BMI and weight change by meta-regression in T2DM. In T1DM, no significant association was found for BMI and placebo response on HbA1c or weight change (Additional file 1: Figure S9).

#### Baseline HbA1c

In T2DM, the HbA1c alteration mediated by placebo treatments was mild in patients with baseline HbA1c below 8.5%, but it reduced more when baseline HbA1c got higher (Additional file 1: Figure S10). The comparison for baseline HbA1c level between HbA1c reduction and elevation subgroups also confirmed a significantly higher baseline HbA1c level in patients with HbA1c reduction (Additional file 1: Table S5). Consistently, meta-regression analysis indicated that higher baseline HbA1c was significantly associated with greater HbA1c reduction by placebo in T2DM (*β* = − 0.09, 95% CI, − 0.16 to − 0.01, *P* = 0.02) (Fig. 4e). However, this association was insignificant after adjusted by age, male percentage, duration of diabetes, study duration, and baseline BMI (*β* = − 0.04, 95% CI, − 0.16 to 0.08, *P* = 0.52). As for weight change, no specific response pattern was observed in T2DM (Additional file 1: Figure S10), although a significant higher baseline HbA1c level was observed in patients with weight elevation versus weight reduction (Additional file 1: Table S5). No significant association was found between baseline HbA1c and HbA1c or weight change in T1DM (Additional file 1: Figure S11).

#### Diabetes duration

Placebo response on HbA1c reduction in T1DM was more profound in patients with diabetes duration between 10 and 30 years (Additional file 1: Figure S12) and a longer diabetes duration was significantly associated with greater HbA1c reduction by placebo (*β* = − 0.02, 95% CI, − 0.03 to − 0.21 × 10^−2^, *P* = 0.03) (Fig. 4f). With different extents of weight reduction observed in diabetes duration subgroups (Additional file 1: Figure S12), no significant association was observed in T1DM.

In T2DM, although moderate HbA1c reduction in placebo treatment was observed in strata of different diabetes duration (Additional file 1: Figure S13), no significant association was found. However, an attenuated magnitude of placebo response on weight reduction was exhibited with increasing diabetes duration (Additional file 1: Figure S13). With significantly shorter diabetes duration observed in patients with weight reduction versus weight elevation (Additional file 1: Table S5), meta-regression analysis suggested a shorter diabetes duration was significantly associated with more weight reduction by placebo (*β* = 0.06, 95% CI, 0.02 to 0.10, *P* < 0.01) (Fig. 4 g).

#### Study duration

In T1DM, modest HbA1c reduction and weight elevation were observed among most study duration strata (Additional file 1: Figure S14). As for T2DM, with the study duration increasing, tendencies of placebo response shifting from HbA1c elevation to HbA1c reduction and from weight reduction to weight gain were displayed (Additional file 1: Figure S15). However, no specific distribution pattern was supported by meta-regression analyses in both T1DM and T2DM.

#### Publication year

In T1DM, HbA1c level in placebo treatment groups was increased in 1990s but decreased afterwards while weight level was reduced first but increased after 2000 (Additional file 1: Figure S16). The absolute alteration values for HbA1c and weight both peaked in 2006–2010 (Additional file 1: Figure S16). In T2DM, HbA1c level in placebo treatment arms tended to elevate modestly from the baseline between 1990 and 2005 but to decrease after 2006 (Additional file 1: Figure S17). As for weight change, except for a relatively modest increase before 1990, the placebo treatments mostly resulted in weight reduction in T2DM (Additional file 1: Figure S17). However, although comparisons showed that publication years of studies with HbA1c elevation and weight elevation in placebo treatment arms were significantly earlier than those with HbA1c and weight reduction (Additional file 1: Table S5), no significant associations were shown by ANOVA tendency tests in both T1DM and T2DM.

## Discussion

This meta-analysis delineated the placebo response in both T1DM and T2DM with data from over 400 RCTs with almost 40,000 participants. More importantly, we characterized the placebo response pattern associated with patient age, sex, disease duration, and possibly disease severity (indirectly reflected by baseline BMI and baseline HbA1c) in diabetes, which tries to raise researchers’ attention again to weighting important moderators and predictors in placebo response.

As is mentioned, placebo response includes all health changes that result after the administration of an inactive treatment, thus including natural history, spontaneous recovery, and regression to the mean [451, 452]. So far, underlying mechanisms of placebo response are not fully understood. Individual patient and clinician factors, mutual interactions, and treatment environment may also somehow influence the placebo response to different extent. As for DM, placebo response can be related to optimal dietary treatment [453–456], or increased physical activity [457, 458], or improved education and management [459, 460], or patients’ expectations [461], in addition to fluctuation of symptoms and regression to the mean.

In this meta-analysis, data of T1DM have been included for analysis for the first time. Interestingly, significantly weight elevation was observed in T1DM patients with placebo treatments while significantly HbA1c reduction and weight reduction were observed in T2DM patients with placebo treatments. Considering all included studies in T1DM were with insulin add-on treatments, such difference in placebo response may be due to the background administration of insulin in T1DM population. Furthermore, we differentiated the oral and injection route for drug administration and found injectable placebo led to more HbA1c reduction compared with oral types in T2DM. It was hypothesized that a stronger signal delivered by subcutaneous puncture stimuli might trigger a positive psychological feedback that enhanced the placebo response, for example, raising patients’ expectations towards treatment benefits. But further evidence was still needed.

Patient age has been shown to consistently affect placebo response with different clinical conditions. Evaluations across medicine found that younger age was associated with higher placebo response, predominantly in psychiatric conditions and internal medicine [462]. In our study, older age was associated with more HbA1c reduction with placebo treatments in T1DM while younger age was associated with more HbA1c reduction with placebo treatments in T2DM. Such difference may be explained by distinct patient age distribution in T1DM and T2DM. In T1DM, adolescences and youth may not be mature enough to understand the disease severity and properly stick to the management routine. With their ages growing, their increased knowledge of DM may help them understand and believe in positive effects from well-organized DM management. In T2DM, patients are generally at a relatively mature age with disease onset. When they get older, less confidence and enthusiasm towards treatments and higher chance of experiencing treatment failure are likely to minimize the placebo response in these patients.

Sex is a controversial moderator for placebo response. Systematic reviews from major medical areas (neurology, psychiatry and internal medicine) but not from diabetes showed that only in 3 analyses female sex was associated with a higher placebo response, indicating poor evidence for contribution of sex to placebo response [463, 464]. But still, Enck et al. proposed that placebo response was predominantly the result of a conditioning learning response in females while a verbal manipulating of expectancies in males [463]. As a supplement, we did discover the sex difference of placebo response in T2DM in terms of HbA1c reduction.

Disease severity is another important associated factor with placebo response. In fact, a positive relationship between disease severity and magnitude of placebo response was previously observed in osteoarthritis [464]. As indirect indicators of disease severity, we found higher baseline BMI and higher baseline HbA1c were significantly associated with more HbA1c reduction achieved by placebo in T2DM, which was different from Khan’s finding [8]. The inconsistence between our results and Khan’s results might be due to different databases for placebo-controlled trials and more complicated groups of add-on treatment in our study. However, such associations turned to be negative after adjusted by other baseline parameters, including age, sex, duration of diabetes, and baseline BMI or HbA1c, which might influence the outcomes. Therefore, the correlations between increased disease severity (baseline BMI and higher baseline HbA1c) and greater HbA1c reduction may be the result of regression to the mean, which is also commonly observed in placebo response. Further investigations are needed to assess the potential link between placebo response and disease severity.

Disease duration is accompanied with alterations of patients’ understanding of disease itself and treatments. The association between the shorter disease duration and the larger placebo response effect size has been reported in fibromyalgia [465]. Similarly, we observed a shorter diabetes duration associated with more weight reduction by placebo in T2DM. It is possibly related to increasing treatment inertia and worsening adherence to nonpharmacological interventions in patients with longer durations. Moreover, worsening interrupted internal environment and neuro-endocrine-immune regulatory network with longer disease duration may also make it harder for placebo response to manifest.

This meta-analysis also has several limitations. First, included studies have different inclusion criteria, various baseline characteristics, non-uniform outcome definitions, and variable sample sizes. These factors will lead to heterogeneity among studies in this meta-analysis. Second, we did not include unpublished trials in our meta-analysis. Published studies may have an inherent bias, as unpublished trials tend to have null effects. However, the reported data from such unpublished trials were likely to be incomplete, espacially for placebo treatment group, which makes it hard for the quality evaluation and subsequent analysis. Therefore, we decide to exclude such studies. Moreover, we cannot fully assess the manipulation of each trials. Uncertainty of possibly inadequate blinding, additional bias from clinicians, known as “placebo by proxy” [466], and influence from underestimated interactions between patients and clinicians may affect the interpretation of our results. In addition, as a meta-analysis, data of HbA1c and body weight change in placebo treatment group were used as the surrogate parameters, but not the data at patient-level, which would be more helpful to draw a conclusion. It is noted that the associations between baseline BMI, HbA1c, and placebo response are still not definite, since the adjusted meta-regression tuned out to be negative. The correlations between the baseline severity and placebo response observed in our meta-analysis may be the result of regression to the mean. Therefore, statistical outcomes for this part should be interpreted with caution. Also, since we did not include studies with untreated control, we could only evaluate the placebo response rather than placebo effect in DM. More investigations, especially studies with untreated control, are needed to accurately record and analyze the placebo response and even placebo effect in DM, and possibly better differentiate the effect of regression to the mean.

## Conclusions

Placebo response in diabetes was systematically outlined in this meta-analysis. Age, sex, and disease duration were associated factors with placebo response in diabetes, which may require more considerations when designing and conducting placebo-controlled clinical trials in diabetes. Baseline disease severity was also associated with placebo response in diabetes, which is likely to be the result of the regression to the mean. More investigations are encouraged to further explore unique or even potentially generalized placebo response patterns in DM and a wider field of medicine concerning more diseases.

## Supplementary information

**Additional file 1: Table S1.** Baseline characteristics of included RCT studies with type 1 diabetes. **Table S2.** Baseline characteristics of included RCT studies with type 2 diabetes. **Table S3.** Risk of bias of included RCT studies with type 1 diabetes. **Table S4.** Risk of bias of included RCT studies with type 2 diabetes. **Table S5.** Baseline characteristics by placebo response with HbA1c and weight change. **Figure S1.** Flowchart of the study. **Figure S2.** Egger’s test for trials with type 1 diabetes. **Figure S3.** Egger’s test for trials with type 2 diabetes. **Figure S4.** Placebo response stratified by patient age in type 1 diabetes mellitus. **Figure S5.** Placebo response stratified by patient age in type 2 diabetes mellitus. **Figure S6.** Placebo response stratified by male percentage in type 2 diabetes mellitus. **Figure S7.** Placebo response stratified by male percentage in type 1 diabetes mellitus. **Figure S8.** Placebo response stratified by baseline BMI in type 2 diabetes mellitus. **Figure S9.** Placebo response stratified by baseline BMI in type 1 diabetes mellitus. **Figure S10.** Placebo response stratified by baseline HbA1c in type 2 diabetes mellitus. **Figure S11.** Placebo response stratified by baseline HbA1c in type 1 diabetes mellitus. **Figure S12.** Placebo response stratified by diabetes duration in type 1 diabetes mellitus. **Figure S13.** Placebo response stratified by diabetes duration in type 2 diabetes mellitus. **Figure S14.** Placebo response stratified by study duration in type 1 diabetes mellitus. **Figure S15.** Placebo response stratified by study duration in type 2 diabetes mellitus. **Figure S16.** Placebo response stratified by publication year in type 1 diabetes mellitus. **Figure S17**. Placebo response stratified by publication year in type 2 diabetes mellitus.

## Data Availability

All data generated or analyzed during this study are included in this article and its supplementary information files.

## Abbreviations

AGI: Alpha glucosidase inhibitor
ANOVA: Analysis of variance
BMI: Body mass index
CI: Confidence interval
DM: Diabetes mellitus
DPP-4: Dipeptidyl peptidase-4
ES: Effect size
GLP-1RA: Glucagon-like peptide-1 receptor agonist
INR: Interquartile range
MET: Metformin
RCT: Randomized controlled trial
SGLT2: Sodium-glucose cotransporter 2
SU: Sulfonylurea
T1DM: Type 1 diabetes mellitus
T2DM: Type 2 diabetes mellitus
