# Supplementary material for: Age, sex, disease severity, and disease duration difference in placebo response: implications from a meta-analysis of diabetes mellitus
Source: BMC Med. 2020 Nov 16;18:322. doi: 10.1186/s12916-020-01787-4 (PMC7667845; doi:10.1186/s12916-020-01787-4)
Supplement: Supplementary file 1 — Additional file 1: Table S1. Baseline characteristics of included RCT studies with type 1 diabetes. Table S2. Baseline characteristics of included RCT studies with type 2 diabetes. Table S3. Risk of bias of included RCT studies with type 1 diabetes. Table S4. Risk of bias of included RCT studies with type 2 diabetes. Table S5. Baseline characteristics by placebo response with HbA1c and weight change. Figure S1. Flowchart of the study. Figure S2. Egger’s test for trials with type 1 diabetes. Figure S3. Egger’s test for trials with type 2 diabetes. Figure S4. Placebo response stratified by patient age in type 1 diabetes mellitus. Figure S5. Placebo response stratified by patient age in type 2 diabetes mellitus. Figure S6. Placebo response stratified by male percentage in type 2 diabetes mellitus. Figure S7. Placebo response stratified by male percentage in type 1 diabetes mellitus. Figure S8. Placebo response stratified by baseline BMI in type 2 diabetes mellitus. Figure S9. Placebo response stratified by baseline BMI in type 1 diabetes mellitus. Figure S10. Placebo response stratified by baseline HbA1c in type 2 diabetes mellitus. Figure S11. Placebo response stratified by baseline HbA1c in type 1 diabetes mellitus. Figure S12. Placebo response stratified by diabetes duration in type 1 diabetes mellitus. Figure S13. Placebo response stratified by diabetes duration in type 2 diabetes mellitus. Figure S14. Placebo response stratified by study duration in type 1 diabetes mellitus. Figure S15. Placebo response stratified by study duration in type 2 diabetes mellitus. Figure S16. Placebo response stratified by publication year in type 1 diabetes mellitus. Figure S17. Placebo response stratified by publication year in type 2 diabetes mellitus. [file 12916_2020_1787_MOESM1_ESM.docx]

**Supplementary materials**

**Table legends**

Table S1. Baseline characteristics of included RCT studies with type 1 diabetes

Table S2. Baseline characteristics of included RCT studies with type 2 diabetes

Table S3. Risk of bias of included RCT studies with type 1 diabetes

Table S4. Risk of bias of included RCT studies with type 2 diabetes

Table S5. Baseline characteristics by placebo response with HbA1c and weight change

**Figure legends**

Figure S1. Flowchart of the study

Figure S2. Egger’s test for trials with type 1 diabetes

Figure S3. Egger’s test for trials with type 2 diabetes

Figure S4. Placebo response stratified by patient age in type 1 diabetes mellitus

Figure S5. Placebo response stratified by patient age in type 2 diabetes mellitus

Figure S6. Placebo response stratified by male percentage in type 2 diabetes mellitus

Figure S7. Placebo response stratified by male percentage in type 1 diabetes mellitus

Figure S8. Placebo response stratified by baseline BMI in type 2 diabetes mellitus

Figure S9. Placebo response stratified by baseline BMI in type 1 diabetes mellitus

Figure S10. Placebo response stratified by baseline HbA1c in type 2 diabetes mellitus

Figure S11. Placebo response stratified by baseline HbA1c in type 1 diabetes mellitus

Figure S12. Placebo response stratified by diabetes duration in type 1 diabetes mellitus

Figure S13. Placebo response stratified by diabetes duration in type 2 diabetes mellitus

Figure S14. Placebo response stratified by study duration in type 1 diabetes mellitus

Figure S15. Placebo response stratified by study duration in type 2 diabetes mellitus

Figure S16. Placebo response stratified by publication year in type 1 diabetes mellitus

Figure S17. Placebo response stratified by publication year in type 2 diabetes mellitus

**Table S1. Baseline characteristics of included RCT studies with type 1 diabetes**

| **Author, year** | **Study duration** | **Treatment group** | **No. of patients** | **Age (years)** | **Men (%)** | **BMI (kg/m2)** | **DM duration (years)** | **Baseline HbA1c (%)** | **Baseline Weight (kg)** |
| --- | --- | --- | --- | --- | --- | --- | --- | --- | --- |
| **Metformin versus placebo in adjunct to insulin treatment** | | | | | | | | | |
| Anderson, 2017(10) | 12 months | MET 1000mg bid+INS | 45 | 14±2.5 | 47 | / | 5.2±3.6 | 8.4±0.95 | / |
|  |  | PBO+INS | 45 | 13.3±2.6 | 44 | / | 5.8±4.1 | 8.8±0.85 | / |
| Codner, 2013(11) | 9 months | MET 850mg bid+INS | 13 | 17.7±1.6 | 0 | 23.7±3 | 9.3±5.1 | 10.3±2.3 | / |
|  |  | PBO+INS | 11 | 16.7±1.7 | 0 | 26.2±5.5 | 5.5±3.1 | 9.6±1.5 | / |
| Hamilton, 2003(12) | 12 weeks | MET 500-1000mg bid+INS | 14 | 15.9±1.9 | 43 | 22.8±4.2 | 9.9±4.4 | 9.3±1.4 | 63.3±13.6 |
|  |  | PBO+INS | 13 | 16±1.7 | 54 | 25.7±2.9 | 7±3.8 | 8.6±0.8 | 71.6±11.7 |
| Jacobsen, 2008(13) | 24 weeks | MET 2000mg qd+INS | 12 | 43.5±13.1 | / | 29.5±2.7 | 17.8±10.3 | 8.85±0.1 | 87.6±13.2 |
|  |  | PBO+INS | 12 | 37.3±9.6 | / | 29.2±2.8 | 20.3±10.2 | 9.34±0.94 | 92±10.2 |
| Lund, 2008(14); Lund, 2009(15) | 52 weeks | MET 2000mg qd+INS | 49 | 46.1±11.6 | 67 | 26.2±3.4 | 30±23 | 9.48±0.99 | 80.5±12.5 |
|  |  | PBO+INS | 51 | 44.9±10.8 | 61 | 25.8±4.3 | 26±25 | 9.6±0.86 | 79±15.3 |
| Libman, 2015(16) | 26 weeks | MET2 000mg qd+INS | 71 | 15.4±1.7 | 38 | / | 7.5±3.6 | 8.8±0.8 | 77±3 |
|  |  | PBO+INS | 69 | 15.1±1.8 | 30 | / | 6.4±3 | 8.8±0.7 | 76±2.5 |
| Meyer, 2002(17) | 24 weeks | MET 850mg bid+INS | 31 | 39.9±12.9 | 55 | 26.4±4.6 | 16.9±8.9 | 7.58±0.84 | 78.4±18.1 |
|  |  | PBO+INS | 31 | 41.1±9.8 | 65 | 25.8±3.6 | 21.6±10.2 | 7.57±0.76 | 74.5±11.7 |
| Nadeau, 2016(18) | 24 weeks | MET 500-2000mg qd+INS | 40 | 15.9±1.7 | / | 23.5±3 | 6.7±3.6 | 9.5±1.3 | 65.7±12.3 |
|  |  | PBO+INS | 40 | 16±1.6 | / | 24.3±4.1 | 6.3±3.5 | 9.4±1.1 | 67.1±13.2 |
| Nwosu, 2015(19) | 36 weeks | MET 1000mg qd+INS | 15 | 15±2.5 | 53.3 | 28.2±6.6 | 5.7±4.4 | 9.3±1.5 | 75.5±25 |
|  |  | PBO+INS | 13 | 14.5±3.1 | 38.5 | 27.5±3.7 | 5.7±5 | 8.7±0.4 | 70.8±17.9 |
| Petrie, 2016(20); Petrie, 2017(21) | 36 months | MET 2000mg qd+INS | 219 | 55.2±8.5 | 59 | 28.4±4.5 | 33.4±11 | 8.1±0.9 | 83.9±15.4 |
|  |  | PBO+INS | 209 | 55.8±8.8 | 59 | 28.5±4.1 | 34.3±10.5 | 8±0.8 | 83.5±13.7 |
| Pitocco, 2013(22) | 24 weeks | MET 850-2550mg qd+INS | 21 | 46±8 | 42.8 | 28.7±2.1 | 9.2±0.7 | 7.24±0.9 | 83±12 |
|  |  | PBO+INS | 21 | 41±10 | 42.8 | 27.3±2 | 8.8±0.8 | 7.73±0.42 | 77±11 |
| Sarnblad, 2003(23) | 12 weeks | MET 500-1000mg bid+INS | 16 | 17.2±1.7 | 31 | 26.2±8.4 | 9.1±5 | 9.3±1.1 | 68.8±17 |
|  |  | PBO+INS | 14 | 16.9±1.4 | 29 | 23.9±6.1 | 7.1±3 | 9.3±1.4 | 66.6±18.3 |
| Ziaee, 2017(24) | 24weeks | MET 500-2000mg qd+INS | / | / | 50 | 23.21±1.4 | / | 8.36±0.8 | / |
|  |  | PBO+INS | / | / | 50 | / | / | / | / |
| **AGI versus placebo in adjunct to insulin treatment** | | | | | | | | | |
| Hollander, 1997(25) | 36 weeks | Acarbose 50-300mg tid+INS | 114 | 37.7±1.1 | 66 | 24.6±0.3 | 15.8±1 | 6.58±0.09 | 73.2±1.2 |
|  |  | PBO+INS | 122 | 36.8±1.1 | 65 | 24.9±0.3 | 13.4±1 | 6.59±0.09 | 73±1.2 |
| Riccardi, 1999(26) | 24 weeks | Acarbose 50-100mg tid+INS | 57 | 32.6±11.78 | 47 | 24.62±3.53 | / | 9.1±1.37 | 66.1±11.58 |
|  |  | PBO+INS | 59 | 36.3±15.35 | 42 | 24.74±3.05 | / | 9.1±1.34 | 68.1±10.54 |
| **TZD versus Placebo in adjunct to insulin treatment** | | | | | | | | | |
| Bhat, 2007(27) | 6 months | Pioglitazone 30mg qd+INS | 30 | 22.4±5.8 | / | 19.7±1.4 | 7.6±4.4 | 7.08±0.48 | 48.7±5.6 |
|  |  | PBO+INS | 30 | 21.5±5.4 | / | 19.6±1.7 | 7.5±4.6 | 7.3±0.37 | 50±6.9 |
| Strowig, 2005(28) | 8 months | Rosiglitazone 4mg bid+INS | 25 | 43.7±13.3 | 64 | 32.7±5.4 | 20.7±13.3 | 7.9±1.3 | 92.7±11.8 |
|  |  | PBO+INS | 25 | 41.1±9.2 | 72 | 31.1±3.1 | 18.1±9.3 | 7.7±0.8 | 96.4±12.2 |
| Tafuri, 2013(29) | 24 weeks | Pioglitazone 15-30mg qd+INS | 8 | 10.2 | 38 | 19.6 | 2.9 | 6.7 | 39.5 |
|  |  | PBO+INS | 7 | 11.7 | 57 | 18.8 | 3.4 | 7.9 | 45 |
| Zdravkovi, 2006(30) | 6 months | Pioglitazone 30mg qd+INS | 18 | 14±1.9 | 50 | 23.4±5.9 | 5.7±3.1 | 8.8±0.8 | / |
|  |  | PBO+INS | 17 | 14.7±2 | 47 | 26.1±5.1 | 7±3.9 | 8.9±1 | / |
| **GLP-1RA versus placebo in adjunct to insulin treatment** | | | | | | | | | |
| Ahren, 2016(31) | 26 weeks | Liraglutide 1.8mg qd+INS | 205 | 43.2±28.5 | 45 | 28.9 | 21.4 | 8.04 | 83.6 |
|  |  | Liraglutide 1.2mg qd+INS | 209 | 42.8±27.5 | 49 | 28.8 | 21.1 | 8.07 | 84.7 |
|  |  | Liraglutide 0.6mg qd+INS | 211 | 43.9±34 | 44 | 28.9 | 21 | 8.09 | 83.1 |
|  |  | PBO+INS | 206 | 42.7±26 | 46 | 28.9 | 20.7 | 8.12 | 84.2 |
| Dejgaard, 2015(32); Dejgaard, 2016(33) | 24 weeks | Liraglutide 1.8mg qd+INS | 50 | 47±13 | 60 | 30.3±3.5 | 20±12 | 8.7±0.7 | 93.4±14.2 |
|  |  | PBO+INS | 50 | 49±12 | 70 | 29.8±3.1 | 25±12 | 8.7±0.7 | 94±12.5 |
| Frandsen, 2015(34); Frandsen, 2017(35) | 12 weeks | Liraglutide 1.8mg qd+INS | 18 | 39.5±2.7 | 61 | 24.17±0.64 | 18.33±2 | 8.8±0.2 | 75.83±2.89 |
|  |  | PBO+INS | 18 | 36.1±1.6 | 72 | 22.75±0.41 | 19.56±1.6 | 8.7±0.1 | 74.89±1.66 |
| Kuhadjya, 2016(36) | 12 weeks | Liraglutide 0.6mg qd+INS | 14 | 45±4 | 64.3 | 26±3 | 19±3 | 7.46±0.19 | 80±4 |
|  |  | Liraglutide 1.2mg qd+INS | 16 | 42±3 | 50 | 33±2 | 21±3 | 7.84±0.17 | 96±4 |
|  |  | Liraglutide 1.8mg qd+INS | 16 | 42±3 | 25 | 28±4 | 20±3 | 7.41±0.15 | 83±4 |
|  |  | PBO+INS | 17 | 50±3 | 41.2 | 28±2 | 19±3 | 7.69±0.17 | 80±6 |
| Mathieu, 2016(37) | 52 weeks | Liraglutide1.8mg qd+INS | 346 | 43.7±13.3 | 47.7 | 29.5±5.2 | 21.5±12.6 | 8.14±0.74 | 86.3±17.3 |
|  |  | Liraglutide1.2mg qd+INS | 346 | 43.9±13.1 | 48.3 | 29.3±5.1 | 21.6±12.2 | 8.16±0.779 | 85.4±17.2 |
|  |  | Liraglutide0.6mg qd+INS | 350 | 43.6±12.8 | 46.9 | 29.5±5.3 | 20.9±12.2 | 8.18±0.738 | 86.5±17.3 |
|  |  | PBO+INS | 347 | 43.4±12.6 | 48.1 | 29.8±5.6 | 21.6±11.8 | 8.15±0.728 | 86.4±17.8 |
| **DPP-4 inhibitors versus placebo in adjunct to insulin treatment** | | | | | | | | | |
| Garg, 2013(38) | 20 weeks | Sitagliptin 100mg qd+INS | 63 | 37±13 | 51 | 27.5±4.9 | 22±11 | 8.2±0.7 | 82±16 |
|  |  | PBO | 62 | 39±15 | 58 | 27.4±4.2 | 20±11 | 8.6±0.7 | 82±15 |
| **SGLT2 inhibitors versus placebo in adjunct to insulin treatment** | | | | | | | | | |
| Buse, 2018(39) | 52 weeks | Sotagliflozin 200mg qd+INS | 263 | 46.6±13.48 | 47.9 | 29.81±5.68 | 25±13.15 | 7.61±0.735 | 86.96±18.539 |
|  |  | Sotagliflozin 400mg qd+INS | 262 | 46.4±13.12 | 45.8 | 29.63±5.29 | 24±12.88 | 7.56±0.724 | 86.5±18.004 |
|  |  | PBO+INS | 268 | 45.2±12.72 | 51.1 | 29.55±5.18 | 24.2±12.38 | 7.54±0.712 | 87.3±17.709 |
| Dandona, 2017(40) | 24 weeks | Dapagliflozin 5mg qd+INS | 259 | 41.9±14.1 | 43 | 28.3±5.8 | 19.7±12.0 | 8.53±0.71 | 80.8±18.2 |
|  |  | Dapagliflozin 10mg qd+INS | 259 | 42.7±14.1 | 50 | 28.1±5.2 | 19.9±11.1 | 8.52±0.64 | 82.0±17.3 |
|  |  | PBO+INS | 260 | 42.7±13.6 | 51 | 28.6±5.2 | 21.2±12.2 | 8.53±0.67 | 84.3±18.3 |
| Danne, 2018(41) | 52 weeks | Sotagliflozin 200mg qd+INS | 261 | 42.3±13.59 | 53.3 | 27.97±5.28 | 18.2±10.82 | 7.74±0.806 | 81.93±17.386 |
|  |  | Sotagliflozin 400mg qd+INS | 263 | 41.7±13.23 | 50.6 | 27.85±4.92 | 18.9±11.18 | 7.71±0.819 | 81.97±17.963 |
|  |  | PBO+INS | 258 | 39.7±13.43 | 51.9 | 27.5±5.17 | 18.1±10.72 | 7.79±0.881 | 81.08±16.857 |
| Famulla, 2016(42);  Pieber, 2015(43) | 4 weeks | Empagliflozin 2.5mg qd+INS | 19 | 41.9±12.4 | 79 | 24.7±3.6 | / | 8.35±0.75 | 75.9±14.2 |
|  |  | Empagliflozin 10mg qd+INS | 19 | 39.6±11.6 | 79 | 27.4±3.5 | / | 8.28±0.79 | 87.1±13.3 |
|  |  | Empagliflozin 25mg qd+INS | 18 | 41.9±9.7 | 56 | 25.4±3.5 | / | 8.15±0.54 | 76.9±14.5 |
|  |  | PBO+INS | 19 | 40.5±10.6 | 68 | 25.4±3.7 | / | 8.18±0.67 | 79.8±13.8 |
| Garg, 2017(44) | 24 weeks | Sotagliflozin 400mg qd+INS | 699 | 43.3±14.2 | 51.2 | 28.29±5.13 | 20.5±12.4 | 8.26±0.96 | 82.4±17.13 |
|  |  | PBO+INS | 703 | 42.4±14.0 | 48.2 | 28.10±5.18 | 19.6±12.1 | 8.21±0.92 | 81.55±17.03 |
| Henry, 2015(45); Rodbard, 2016(46); Peters, 2016(47) | 18 weeks | Canagliflozin 100mg qd+INS | 117 | 42.0±11.6 | 59 | 28.0±3.9 | 22.0±11.5 | 7.9±0.5 | 84.1±14.2 |
|  |  | Canagliflozin 300mg qd+INS | 117 | 42.8±11.0 | 55.6 | 28.1±3.9 | 21.9±10.6 | 8.0±0.5 | 82.9±15.0 |
|  |  | PBO+INS | 117 | 42.0±11.9 | 53.8 | 28.0±3.6 | 23.3±11.0 | 7.9±0.6 | 83.0±15.4 |
| Kuhadjya, 2016(48) | 12 weeks | Dapagliflozin 10mg qd+INS | 17 | 55±3 | 70 | 31±1 | 25±3 | 7.8±0.21 | 85±3.8 |
|  |  | PBO+INS | 9 | 52±3 | 50 | 27±2 | 31±5 | 7.4±0.2 | 79±3.8 |
| Sands, 2015(49) | 4 weeks | Sotagliflozin 400mg+INS | 16 | 45.5±17 | 50 | 27.1±3.1 | 16.8±19.7 | 7.94±0.55 | 9.45±3.45 |
|  |  | PBO+INS | 17 | 34.0±13 | 47 | 26.2±3.0 | 18.5±18.05 | 7.98±0.51 | 8.89±3.96 |
| Shimada, 2018 (50) | 4 weeks | Empagliflozin 2.5mg qd+INS | 13 | 44.2±12.6 | 38.5 | 24.4±3.93 | 16.8±1.2 | 8.02±0.36 | 63.3±10.5 |
|  |  | Empagliflozin 10mg qd+INS | 12 | 44.5±11.8 | 33.3 | 22.68±3.27 | 14.3±8.4 | 8.12±0.37 | 59.9±10.6 |
|  |  | Empagliflozin 25mg qd+INS | 12 | 46.6±10.8 | 66.7 | 22.6±2.7 | 20.8±13.5 | 7.89±0.91 | 60.5±10.2 |
|  |  | PBO+INS | 11 | 43.9±11.7 | 45.5 | 23.7±2.6 | 14.8±10.0 | 8.23±0.47 | 63.6±7.7 |
| Mathieu, 2018 (51) | 24 weeks | Dapagliflozin 5mg qd+INS | 271 | 42.7±13.4 | 43.5 | 27.3±5.1 | 19.3±11.8 | 8.45±0.69 | 78.8±17.4 |
|  |  | Dapagliflozin 10mg qd+INS | 270 | 42.4±12.8 | 44.8 | 27.8±5.5 | 19.4±11.9 | 8.43±0.69 | 80.1±18.3 |
|  |  | PBO+INS | 272 | 43.0±13.7 | 43.8 | 27.6±5.4 | 19.0±11.6 | 8.43±0.65 | 78.9±18.9 |
| Rosenstock, 2018 (52) | 52 weeks | Empagliflozin 10mg qd+INS | 243 | 45.7±12.5 | 48.6 | 29.5±5.5 | 22.8±12.6 | 8.10±0.60 | 86.2±18.2 |
|  |  | Empagliflozin 25mg qd+INS | 241 | 45.3±13.9 | 46.1 | 29.5±6.0 | 22.5±13.0 | 8.06±0.53 | 86.6±18.3 |
|  |  | PBO+INS | 239 | 44.5±13.5 | 45.6 | 28.5±5.3 | 22.4±12.4 | 8.13±0.57 | 83.4±15.3 |
|  | 24 weeks | Empagliflozin 2.5mg qd+INS | 237 | 43.4±14.2 | 49.8 | 28.0±4.4 | 20.8±11.9 | 8.14±0.61 | 81.6±14.6 |
|  |  | Empagliflozin 10mg qd+INS | 244 | 42.4±13.3 | 46.7 | 28.7±5.1 | 20.5±11.9 | 8.19±0.64 | 83.7±17.0 |
|  |  | Empagliflozin 25mg qd+INS | 242 | 44.2±13.5 | 50.8 | 28.4±5.6 | 21.2±11.4 | 8.19±0.65 | 83.3±18.9 |
|  |  | PBO+INS | 238 | 42.2±13.2 | 47.9 | 27.8±5.1 | 21.7±13.0 | 8.19±0.58 | 8071±16.9 |
| **Pramlintide versus placebo in adjunct to insulin treatment** | | | | | | | | | |
| Edelman, 2006(53); Marrero, 2007(54); Kovatchev, 2008(55) | 29 weeks | Pramlintide 15-60ug tid+INS | 148 | 41±14 | 48.6 | 27.7±4.6 | 19±12 | 8.1±0.8 | 81±17 |
|  |  | PBO+INS | 147 | 41±12 | 40.8 | 27.8±4.8 | 21±12 | 8.1±0.8 | 81±17 |
| Herrmann, 2013(56) | 29 weeks | Pramlintide 30-60ug tid+INS | 82 | 42±14 | 39 | 27±4 | 20±12 | 8.1±0.7 | 79±16 |
|  |  | PBO+INS | 73 | 41±12 | 29 | 28±5 | 24±12 | 8±0.8 | 80±17 |
| Ratner, 2004(57) | 52 weeks | Pramlintide 60ug tid+INS | 164 | 39.2±13.1 | 52 | 26.4±4.5 | 18.6±10.7 | 8.9±1.1 | 77.3±14.6 |
|  |  | Pramlintide 60ug qid+INS | 161 | 41.9±13.1 | 52 | 26.8±4.4 | 19.2±10.7 | 8.9±1 | 78.3±14.5 |
|  |  | Pramlintide 90ug tid+INS | 172 | 41±12.8 | 47 | 26.3±4.1 | 18.6±11.4 | 8.9±0.9 | 75.8±14.7 |
|  |  | PBO+INS | 154 | 41.3±13.6 | 53 | 26.5±4.9 | 18.2±10.5 | 9±1.1 | 76.9±15.8 |
| Ratner, 2005(58) | 26 weeks | Pramlintide30/60 tid/qid+INS | 281 | 41±12 | 50 | 25.7±3.6 | 18±10 | 7.9±0.4 | 75.4±13.1 |
|  |  | PBO+INS | 196 | 42±13 | 55 | 25.8±4.0 | 19±11 | 7.9±0.4 | 76.0±14.3 |
| Whitehouse,  2002(59) | 52 weeks | Pramlintide 30ug qid+INS | 243 | 40.3±11.6 | 55 | 25.2±3.3 | 16.5±10 | 8.7±1.3 | 75±13.8 |
|  |  | PBO+INS | 237 | 40.4±12.1 | 55 | 25.8±3.5 | 17.1±10.5 | 8.9±1.5 | 75.6±13.3 |

**Table S2. Baseline characteristics of included RCT studies with type 2 diabetes**

| **Author, year** | **Study duration** | | **Treatment group** | **No. of patients** | **Age (years)** | **Men (%)** | **BMI (kg/m^2^)** | **DM duration (years)** | **Baseline HbA1c (%)** | | **Baseline Weight (kg)** |  |  |
| --- | --- | --- | --- | --- | --- | --- | --- | --- | --- | --- | --- | --- | --- |
| **Exclusive PBO effect (monotherapy)** | | | | | | | | | | | |  |  |
| **SU versus PBO** | | | | | | | | | | | | |  |
| Madsbad, 2004(60) | | 12 weeks | Glimepiride 4mg qd | 26 | 57±9.2 | 61.5 | 30.2±4.6 | 3.4±2.9 | 7.4±1.2 | | / |  |  |
|  | |  | PBO | 29 | 57±9.4 | 69 | 30.3±4.2 | 3.8±3.4 | 7.8±0.9 | | / |  |  |
| Scott, 2007(61) | | 12 weeks | Glipizide 20mg qd | 123 | 54.7±10.7 | 56.9 | 30.6±5.3 | 4.7±4.2 | 7.9±1 | | / |  |  |
|  | |  | PBO | 125 | 55.3±9.7 | 62.4 | 31.6±5.8 | 4.7±4.2 | 7.9±1.0 | | / |  |  |
| Goldberg, 1996(62) | | 14 weeks | Glimepiride 4mg qd | 76 | 57.8 | 53.9 | / | 5 | 7.7 | | 86.1 |  |  |
|  |  |  | PBO | 73 | 60.4 | 64.9 | / | 6 | 7.8 | | 85.0 |  |  |
| Rosenstock, 1996(63) | | 14 weeks | Glimepiride 8mg qd | 88 | 61.8±9.9 | 74 | / | 7 | 8.1±1.0 | | / |  |  |
|  |  |  | PBO | 79 | 61.1±9.7 | 67 | / | 6 | 8.0±1.1 | | / |  |  |
| Simonson, 1997(64) | | 16 weeks | Glipizide 20mg qd | 69 | 59.3 | 68.1 | 28.8 | 7.8 | 8.7±0.2 | | 84.8 |  |  |
|  |  |  | PBO | 69 | 60.2 | 76.8 | 29.7 | 7.5 | 8.3±0.2 | | 87.1 |  |  |
| Fischer, 2003(65) | | 16 weeks | Glibenclamide 1mg tid | 27 | 58.1±7.0 | 59.3 | / | 5.8±0.8 | 8.3±0.2 | | 79.3±2.4 |  |  |
|  |  |  | PBO | 25 | 58.6±6.3 | 68 | / | 6.4±0.9 | 8.3±0.2 | | 79.2±1.8 |  |  |
| Hanefeld, 2002(66) | | 16 weeks | Glibenclamide 1mg tid | 8 | 60.6±2.5 | 62.5 | 27.1±1.1 | 7.1±1.2 | 8.4±0.4 | | / |  |  |
|  |  |  | PBO | 8 | 59±1.6 | 75 | 27.2±1.1 | 6.8±1.6 | 8.7±0.6 | | / |  |  |
| Hoffmann, 1994(67) | | 24 weeks | Glibenclamide 4.3mg qd | 27 | 59.5±5.7 | 48 | 26.5±2.1 | 1.4±1.1 | 8.30±0.37 | | / |  |  |
|  |  |  | PBO | 30 | 56.9±6.7 | 40 | 26.8±1.5 | 1.0±0.9 | 8.29±0.37 | / | |  |  |
| Segal, 1997(68) | | 24 weeks | Glibenclamide 3.5mg bid | 37 | 56 | 62.2 | 29.2 | / | 7.96 | / | |  |  |
|  |  |  | PBO | 42 | 59 | 57.1 | 29.1 | / | 8.25 | / | |  |  |
| Ebeling, 2001(69) | | 6 months | Glibenclamide 2.5mg qd | 10 | / | / | 30.2±1.7 | / | 8.9±0.3 | / | |  |  |
|  |  |  | PBO | 10 | / | / | 31.9±1.5 | / | 8.6±0.2 | / | |  |  |
| Coniff, 1995(70) | | 36 weeks | Tolbutamide | 66 | 55.4 | 56 | 29.5 | 5.6 | 6.95 | 84.8 | |  |  |
|  |  |  | PBO | 62 | 56.3 | 52 | 29.9 | 5.5 | 7.1 | 85.8 | |  |  |
| **MET versus PBO** | |  |  |  |  |  |  |  |  |  | |  |  |
| Johnson, 1993(71) | | 12 weeks | MET 850mg tid | 4 | / | / | 30.1±2.4 | / | 11.2±2.0 | 81.3±10.9 | |  |  |
|  | |  | PBO | 4 | / | / |  | / |  |  | |  |  |
| Tessari, 1994(72) | | 12 weeks | MET 850mg bid | 11 | 53±3 | 54.5 | 28±1 | / | 8.0±0.6 | / | |  |  |
|  | |  | PBO | 6 | 60±3 | 33.3 | 28±1 | / | 6.7±0.3 | / | |  |  |
| List, 2009(73) | | 12 weeks | MET 1500mg qd | 56 | 54±9 | 48 | 32±5 | / | 7.6±0.8 | 88±20 | |  |  |
|  | |  | PBO | 54 | 53±11 | 56 | 32±5 | / | 7.9±0.9 | 89±18 | |  |  |
| Fonseca, 2013(74) | | 12 weeks | MET 1500mg qd | 69 | 53.1±11.7 | 58 | 29.8±5.5 | 4.13±4.71 | 8.03±0.90 | 84.1±21.8 | |  |  |
|  | |  | PBO | 69 | 53.4±9.7 | 46.4 | 30.9±5.5 | 4.64±5.93 | 7.84±0.78 | 81.8±17.4 | |  |  |
| Garber, 1997(75) | | 14 weeks | MET 1500mg qd | 76 | 59±10 | 63 | / | / | 9.7±1.5 | 89.6±16.2 | |  |  |
|  | |  | PBO | 79 | 55±11 | 56 | / | / | 9.9±1.9 | 90.9±20.1 | |  |  |
| Natali, 2004(76) | | 16 weeks | MET 1500mg qd | 28 | 58±10 | 78.6 | 28.0±3.5 | 6.3±5.3 | 7.8±1.1 | 77.3±12.5 | |  |  |
|  | |  | PBO | 22 | 58±9 | 81.8 | 30.2±3.1 | 3.4±3.4 | 7.6±0.8 | 86.9±10.5 | |  |  |
| Fujioka, 2005(77) | | 16 weeks | MET 1000mg bid | 123 | 57±10 | 47 | 30.6±4.6 | 3.1±2.7 | 8.4±1.1 | / | |  |  |
|  | |  | PBO | 117 | 54±10 | 49 | 30.7±4.1 | 2.7±2.7 | 8.3±1.1 | / | |  |  |
| Hoffmann, 1997(78) | | 24 weeks | Metformin | 31 | 55.9±7.8 | 45 | 27.4±2.2 | 2.1±1.5 | 9.7±0.9 | 79±8.8 | |  |  |
|  | |  | PBO | 32 | 60.2±8.6 | 38 | 26.3±2.2 | 3.6±2.8 | 9.4±0.9 | 74.9±9.7 | |  |  |
| Horton, 2000(7[9](#_ENREF_20)) | | 24 weeks | MET 500mg tid | 178 | 56.8±10.9 | 68.5 | 29.6±4.3 | 4.5±5.5 | 8.4±1.2 | / | |  |  |
|  | |  | PBO | 172 | 59.6±10.9 | 60.5 | 29.2±3.9 | 4.6±4.7 | 8.3±1.1 | / | |  |  |
| Fujioka, 2005(77) | | 24 weeks | MET 1000mg qd | 161 | 55±11 | 53 | 28.7±3.9 | 3.3±2.8 | 8.1±0.9 | / | |  |  |
|  | |  | PBO | 79 | 58±11 | 63 | 28.9±3.5 | 3.2±2.6 | 7.9±0.9 | / | |  |  |
| Goldstein, 2007(80) | | 24 weeks | MET 1000mg bid | 177 | / | / | / | / | 8.68±0.91 | / | |  |  |
|  | |  | PBO | 165 | / | / | / | / | 8.68±1.00 | / | |  |  |
| Haak, 2012(81) | | 24 weeks | MET 1000mg bid | 138 | 55.2±10.6 | 53.1 | 29.5±5.3 | / | 8.5±0.9 | 80±18.5 | |  |  |
|  | |  | PBO | 72 | 55.7±11.0 | 50 | 28.6±5.2 | / | 8.7±1.0 | 76.8±17.5 | |  |  |
| Hällsten, 2002(82) | | 26 weeks | MET 1000mg bid | 13 | 57.8±2.2 | 61.5 | 29.9±1.1 | / | 6.9±0.2 | / | |  |  |
|  | |  | PBO | 14 | 57.7±1.9 | 71.4 | 30.3±1.2 | / | 6.3±0.1 | / | |  |  |
| Viljanen, 2005(83) | | 26 weeks | MET 1000mg bid | 12 | 57.8±8.7 | 58.3 | 29.6±4.0 | / | 6.9±0.9 | 87.6±10.7 | |  |  |
|  | |  | PBO | 11 | 58.7±8.3 | 81.8 | 29.8±4.1 | / | 6.2±0.7 | 89.1±9.3 | |  |  |
| DeFronzo, 1995(84) | | 29weeks | MET 2550mg qd | 143 | 53±1 | 43.4 | 29.9±0.3 | 6.0±0.5 | 8.4±0.1 | 94.4±1.1 | |  |  |
|  | |  | PBO | 146 | 53±1 | 42.5 | 29.2±0.3 | 6.0±0.6 | 8.2±0.2 | 92.2±1.2 | |  |  |
| Dornan, 1991(85) | | 8 months | MET 1000g tid | 30 | 55±1 | / | 30±1 | / | 11.7±0.4 | 84.6±2.7 | |  |  |
|  | |  | PBO | 30 | 55±1 | / | 30±1 | / | 11.8±0.4 | 79.5±2.5 | |  |  |
| Chiasson, 2001(86) | | 36 weeks | MET 500mg tid | 83 | 57.9±8.6 | 73.5 | 30.7±5.1 | 7.5±7.4 | 8.2±0.9 | 89±17.8 | |  |  |
|  | |  | PBO | 83 | 57.7±9.9 | 67.5 | 31.1±4.4 | 5.1±4.9 | 8.1±0.7 | 88.6±14.1 | |  |  |
| **AGI versus PBO** | |  |  |  |  |  |  |  |  |  | |  |  |
| Wagner, 2006(87) | | 12 weeks | Acarbose 100mg tid | 14 | 57(52-58) | 57.1 | 28.7 | 3.5(1-6) | 5.9±0.75 | 53.1 | |  |  |
|  | |  | PBO | 17 | 54(50-58) | 82.3 | 28.7 | 4(2-5) | 6.6±0.5 | 60.5 | |  |  |
| Coniff, 1995(88) | | 16 weeks | Acarbose 100mg tid | 58 | 55 | 53 | 31 | 6 | 8.69 | 85.9 | |  |  |
|  | |  | PBO | 64 | 54 | 58 | 32 | 5 | 8.67 | 91.3 | |  |  |
| Calle-Pascuac, 1996(89) | | 16 weeks | Acarbose 100mg tid | 20 | / | / | 35.3±8.8 | / | 6.3±0.8 | 88.7±18.2 | |  |  |
|  |  |  | PBO | 20 | / | / | 35.9±7.6 | / | 6.4±1.3 | 89.5±16.1 | |  |  |
| Scott, 1999(90) | | 16 weeks | Acarbose 100mg tid | 53 | 56±9 | 62 | 31.0±3.0 | 1.75±1.25 | 7.0±0.87 | 91±13 | |  |  |
|  | |  | PBO | 52 | 57±8 | 65 | 29.0±3.0 | 2.17±1.42 | 6.89±0.85 | 84±12 | |  |  |
| Hanefeld, 2002(66) | | 16 weeks | Acarbose 100mg tid | 11 | 60.4±1.3 | 91 | 27.5±0.7 | 7.7±1.3 | 8.2±0.3 | / | |  |  |
|  | |  | PBO | 8 | 59±1.6 | 75 | 27.2±1.1 | 6.8±1.6 | 8.7±0.6 | / | |  |  |
| Fischer, 2003(65) | | 16 weeks | Acarbose 100mg tid | 25 | 59.4±5.6 | / | 27.3±0.8 | 7.8±0.9 | 8.1±0.2 | 78.2±2.3 | |  |  |
|  | |  | PBO | 25 | 58.6±6.3 | / | 27.0±0.7 | 6.4±0.9 | 8.3±0.3 | 79.2±1.8 | |  |  |
| Delgado, 2002(91) | | 4 months | Acarbose 100mg qd | 9 | / | / | 36.1±1.5 | / | 6.8±0.5 | 103.8±2.9 | |  |  |
|  | |  | PBO | 8 | / | / | 34.4±2.8 | / | 7.5±0.6 | 90.6±8.9 | |  |  |
| Hanefeld, 2009(92) | | 20 weeks | Acarbose 100mg tid | 42 | 62.3±8.7 | / | 31.02±5.12 | / | 6.11±0.48 | / | |  |  |
|  | |  | PBO | 45 | 59.9±10.0 | / | 30.78±3.70 | / | 6.09±0.66 | / | |  |  |
| Rosenbaum, 2002(93) | | 22 weeks | Acarbose 300mg qd | 20 | 59.8±8.2 | 30 | 30.3±2.9 | 6.8 | 6.4±1.7 | 75.1±11.6 | |  |  |
|  |  |  | PBO | 20 | 62±9.7 | 40 | 31.7±3.9 | 6.8 | 6.3±2.1 | 80.2±9.8 | |  |  |
| Hotta, 1993(94) | | 24 weeks | Acarbose 100mg tid | 20 | 49.8 | 26.3 | / | 4.6 | 11.1 | / | |  |  |
|  | |  | PBO | 20 | 47.9 | 22.2 | / | 4.4 | 10.3 | / | |  |  |
| Hoffmann, 1994(67) | | 24 weeks | Acarbose 100mg tid | 28 | 58.8±6.9 | 46 | 26.5±1.6 | 12.7±10.8 | 8.29±0.42 | / | |  |  |
|  | |  | PBO | 30 | 56.9±6.7 | 40 | 26.8±1.5 | 12.1±10.8 | 8.29±0.37 | / | |  |  |
| Hoffmann, 1997(78) | | 24 weeks | Acarbose 100mg tid | 31 | 58.9±9.4 | 19 | 26.4±2.7 | 3.0±2.3 | 9.6±0.9 | 73.9±10.3 | |  |  |
|  | |  | PBO | 32 | 60.2±8.6 | 38 | 26.3±2.2 | 3.6±2.8 | 9.4±0.9 | 74.9±9.7 | |  |  |
| Segal, 1997(68) | | 24 weeks | Miglitol | 40 | 61 | 55 | 28.6 | / | 7.95 | / | |  |  |
|  | |  | PBO | 42 | 59 | 57.1 | 29.1 | / | 8.25 | / | |  |  |
| Chan, 1998(95) | | 24 weeks | Acarbose 100mg tid | 63 | 52.8±10.2 | 50.8 | / | 2.7±3.5 | 8.2±1.0 | 64.1±10 | |  |  |
|  | |  | PBO | 63 | 54±10 | 50.8 | / | 2.1±3.4 | 8.6±1.1 | 65.4±13.3 | |  |  |
| Gentile, 2001(96) | | 28 weeks | Acarbose 100mg tid | 52 | / | / | / | / | 8.9±0.8 | 78±6 | |  |  |
|  | |  | PBO | 48 | / | / | / | / | 8.7±0.9 | 76±4 | |  |  |
| Coniff, 1994(97) | | 36 weeks | Acarbose 50-300mg tid | 91 | 56.0±1.0 | 54 | 32 | 4 | 6.78 | 93.4±1.8 | |  |  |
|  | |  | PBO | 98 | 55.6±1.0 | 45 | 31.5 | 3 | 6.65 | 94.5±1.7 | |  |  |
| Coniff, 1995(70) | | 36 weeks | Acarbose 200mg tid | 67 | 56.2 | 39 | 29.7 | 5.1 | 6.88 | 81.6 | |  |  |
|  | |  | PBO | 62 | 56.3 | 52 | 29.9 | 5.5 | 7.1 | 85.8 | |  |  |
| Chiasson, 2001(86) | | 36 weeks | PBO | 83 | 57.7±9.9 | 67.5 | 31.1±4.5 | 5.1±4.9 | 8.1±0.7 | 91±15.5 | |  |  |
|  | |  | Miglitol | 89 | 57.3±9 | 78 | 31.1±4.4 | 5.2±4.7 | 8.2±0.9 | 88.6±14.1 | |  |  |
| Chiasson, 1994(98) | | 1 year | Acarbose 200mg qd | 38 | / | / | 28.8±0.5 | / | 6.7±0.2 | 84.5±1.2 | |  |  |
|  | |  | PBO | 39 | / | / | 28.8±0.5 | / |  | 81.3±1.2 | |  |  |
| Meneilly, 2000(99) | | 12 months | Acarbose 100mg tid | 22 | 68±1 | / | 29.0±1.0 | / | 7.3±0.1 | / | |  |  |
|  | |  | PBO | 23 | 70±1 | / | 28.0±1.0 | / | 7±0.2 | / | |  |  |
| Josse, 2003(100) | | 12 months | Acarbose 100mg tid | 93 | 69.7±0.5 | / | 28.3±0.4 | 5.8±0.7 | 7.4±0.1 | 79.4±1.3 | |  |  |
|  | |  | PBO | 99 | 70.3±0.5 | / | 28.6±0.4 | 4.8±0.5 | 7.3±0.1 | 81.3±1.6 | |  |  |
| Hasche, 1999(101) | | 24 months | Acarbose 100mg tid | 52 | / | / | 26.9±3.1 | / | 8.9±0.8 | 74.9±8.7 | |  |  |
|  | |  | PBO | 48 | / | / | 26.2±2.4 | / | 8.7±0.9 | 74.5±8.6 | |  |  |
| **TZD versus PBO** | |  |  |  |  |  |  |  |  |  | |  |  |
| Iwamoto, 1996(102) | | 12 weeks | Troglitazone 400mg qd | 136 | 54.6±10.1 | 50.7 | 24.1±3.5 | 6.3±4.4 | 8.61±1.51 | 61±11 | |  |  |
|  | |  | PBO | 126 | 57.4±9.3 | 53.2 | 24.7±3.4 | 7.5±5.4 | 8.51±1.46 | 61.8±10.4 | |  |  |
| Kumar, 1996(103) | | 12 weeks | Troglitazone 400mg bid | 49 | 56 | 57.1 | 27.7±4.0 | 6 | 6.9 | / | |  |  |
|  | |  | PBO | 49 | 57 | 73.5 | 28.9±4.6 | 7 | 7.2 | / | |  |  |
| Patel, 1999(104) | | 12 weeks | Rosiglitazone 2.0mg bid | 80 | 59.7±10.0 | 68.8 | 28.4±4.09 | 5.8 | 9 | / | |  |  |
|  | |  | PBO | 75 | 56.8±11.5 | 69.3 | 28.9±3.98 | 4.2 | 9.1 | / | |  |  |
| Raskin, 2000(105) | | 12 weeks | Rosiglitazone 2mg bid | 73 | 58.47±9.8 | 61.6 | 30.15±4.68 | 5.6±5.93 | 8.7±1.44 | / | |  |  |
|  | |  | PBO | 69 | 60.06±9.4 | 59.4 | 30.44±4.15 | 5.6±5.19 | 8.7±1.63 | / | |  |  |
| Miyazaki, 2001(106) | | 12 weeks | Rosiglitazone 8mg qd | 15 | / | / | 30.0±1.1 | / | 8.7±0.4 | 86±4 | |  |  |
|  | |  | PBO | 14 | / | / | 30.1±1.0 | / | 8.3±0.4 | 87±5 | |  |  |
| Juhl, 2003(107) | | 12 weeks | Rosiglitazone 4mg bid | 10 | 54±9 | 90 | 30.0±2.7 | / | 7.0±1.4 | 92±7 | |  |  |
|  | |  | PBO | 10 | 54±9 | 60 | 31.7±1.9 | / | 6.8±1.0 | 95±10 | |  |  |
| Wallace, 2004([108](#_ENREF_49)) | | 12 weeks | Pioglitazone 45mg qd | 19 | 61.4±6.3 | 73.7 | 29.8±4.5 | 2.6 | 6.7±0.9 | 90.7±3.6 | |  |  |
|  | |  | PBO | 11 | 62.6±10.0 | 72.7 | 28.9±2.8 | 2.5 | 6.7±0.9 | 85.2±4.3 | |  |  |
| Gastaldelli, 2006(109) | | 12 weeks | Rosiglitazone 8mg qd | 13 | 53±2 | 53.8 | 29.3±1.2 | 4±2 | 8.6±0.5 | / | |  |  |
|  | |  | PBO | 13 | 56±2 | 61.5 | 30.2±1.0 | 3±1 | 8.2±0.4 | / | |  |  |
| Sourij, 2006(110) | | 12 weeks | Pioglitazone 30mg qd | 21 | / | / | / | / | 6.1±0.6 | / | |  |  |
|  | |  | PBO | 21 | / | / | / | / | 6.1±0.5 | / | |  |  |
| Oz Gul, 2008(111) | | 12 weeks | Rosiglitazone 4mg qd | 11 | / | / | 28.3±4.09 | / | 7.0±1.07 | / | |  |  |
|  | |  | PBO | 10 | / | / | 29.2±2.3 | / | 6.39±1.1 | / | |  |  |
| Oz Gul, 2010(112) | | 12 weeks | Rosiglitazone 4mg qd | 20 | / | / | 29.6±4.8 | / | 7.3±1.3 | / | |  |  |
|  | |  | PBO | 21 | / | / | 29.6±4.1 | / | 7.3±0.9 | / | |  |  |
| Kong, 2011(113) | | 12 weeks | Pioglitazone 30mg | 37 | 53.6±7.6 | 56.8 | 24.86±3.26 | 5.59±4.60 | 7.49±0.82 | 64.09±10.79 | |  |  |
|  | |  | PBO | 32 | 54.0±8.5 | 59.4 | 25.53±4.03 | 5.85±3.89 | 7.35±0.62 | 67.71±13.7 | |  |  |
| Colca, 2013(114) | | 12 weeks | Pioglitazone 45mg | 55 | 55 | 56 | / | / | 8.15 | 88 | |  |  |
|  | |  | PBO | 56 | 53 | 48 | / | / | 7.98 | 88 | |  |  |
| Ebeling, 1999(115) | | 16 weeks | Troglitazone 400mg qd | 15 | 62.6±2.2 | 33.3 | 32.3±1.3 | 15.9±2.1 | 8.7±0.3 | / | |  |  |
|  | |  | PBO | 12 | 63.5±2.8 | 50 | 33.1±1.0 | 14.3±1.9 | 8.8±0.3 | / | |  |  |
| Miyazaki, 2001(116) | | 16 weeks | Pioglitazone 45mg qd | 12 | / | / | 28.7±1.1 | / | 8.9±0.3 | 84.8±3.6 | |  |  |
|  | |  | PBO | 11 | / | / | 29.5±1.3 | / | 7.9±0.3 | 81.4±5 | |  |  |
| Phillips, 2001(117) | | 16 weeks | Rosiglitazone 4mg bid | 187 | 56.5±9.7 | 65.2 | 29.9±4.3 | 5.9±6.1 | 9.0±1.5 | / | |  |  |
|  | |  | PBO | 173 | 57.7±9.2 | 68.8 | 29.1±4.2 | 6.6±6.9 | 8.9±1.5 | / | |  |  |
| Rosenblatt, 2001(118) | | 16 weeks | Pioglitazone 30mg qd | 101 | 53.8±10.0 | 50.5 | 31.5±4.7 | / | 10.65±1.77 | 89.8±18.0 | |  |  |
|  | |  | PBO | 96 | 55.2±10.0 | 56.2 | 30.7±5.0 | / | 10.42±1.70 | 87.2±18.4 | |  |  |
| Carey, 2002(119) | | 16 weeks | Rosiglitazone 8mg qd | 16 | 54.2±11.1 | 87.5 | 29.8±4.0 | 3.3±4.5 | 7.8±1.3 | 93.9±14.1 | |  |  |
|  | |  | PBO | 17 | 57.9±10.7 | 76.5 | 31.3±3.6 | 3.1±3.3 | 7.1±1.4 | 89.5±12.5 | |  |  |
| Rosenstock,2002(120) | | 16 weeks | Troglitazone 600mg qd | 150 | 58 | 59 | 21-38 | 0.1-33.4 | 8.4±1.1 | / | |  |  |
|  | |  | PBO | 148 | 58 | 59 | 20-38 | 0.2-37.9 | 8.2±1.2 | / | |  |  |
| Natali, 2004(76) | | 16 weeks | Rosiglitazone 8mg qd | 24 | 59±7 | 91.7 | 27.6±2.8 | 6.5±4.5 | 7.2±1.2 | 80.4±10.1 | |  |  |
|  | |  | PBO | 22 | 58±9 | 81.8 | 30.2±3.1 | 3.4±3.4 | 7.6±0.8 | 86.9±10.5 | |  |  |
| Lautamäki, 2005(121) | | 16 weeks | Rosiglitazone 8mg qd | 27 | 64.1±7.8 | 70.4 | 29.6±4.7 | 6.7±6.4 | 7.3±0.9 | 85.3±17.4 | |  |  |
|  | |  | PBO | 27 | 63.2±7.4 | 70.4 | 29.6±3.4 | 6.8±5.9 | 7.1±0.9 | 89.1±14.3 | |  |  |
| Gastaldelli, 2007(122) | | 4 months | Rosiglitazone 8mg qd | 12 | 55±3 | 50 | 29.2±1.3 | 4±2 | 8.7±0.5 | / | |  |  |
|  | |  | PBO | 12 | 56±2 | 66.7 | 29.8±1.2 | 2±1 | 8.1±0.4 | / | |  |  |
| Tan, 2005(123) | | 24 weeks | Rosiglitazone 4mg bid | 6 | / | / | 30.83±1.11 | / | 7.17±0.26 | / | |  |  |
|  | |  | PBO | 6 | / | / | 30.8±1.04 | / | 7.52±0.38 | / | |  |  |
| Miyazaki, 2002(124) | | 26 weeks | Pioglitazone 30mg | 11 | 51±2 | 72.7 | 32.2±1.6 | / | 8.5±0.5 | 97±4 | |  |  |
|  | |  | PBO | 11 | 58±3 | 27.3 | 32.8±1.6 | / | 8.6±0.5 | 90±4 | |  |  |
| Fonseca, 1998(125) | | 26 weeks | Troglitazone 400mg | 19 | 54±11 | 59.2 | 32.4±6.6 | 5.3±5.6 | 0.34±0.36 | / | |  |  |
|  | |  | PBO | 18 |  |  |  |  | 0.40±0.40 | / | |  |  |
| Fonseca, 1998(126) | | 26 weeks | Troglitazone 600mg qd | 18 | 60.4±5.9 | 44.4 | 37.3±9.2 | / | 9.5±2 | / | |  |  |
|  | |  | PBO | 8 | 52.6±7.5 | 37.5 | 39.6±13.4 | / | 10.1±1.43 | / | |  |  |
| Aronoff, 2000(127) | | 26 weeks | Pioglitazone 30mg | 85 | / | / | / | / | 10.2±0.21 | 90.3±1.58 | |  |  |
|  | |  | PBO | 79 | / | / | / | / | 10.4±0.22 | 90.4±1.47 | |  |  |
| Haffner, 2002(128) | | 26 weeks | Rosiglitazone 8mg qd | 136 | 60.4±9.3 | 47.8 | 29.5±3.8 | 4.9±5.2 | 8.6±1.5 | / | |  |  |
|  | |  | PBO | 95 | 59.8±10.5 | 61.1 | 30.1±3.9 | 4.5±4.8 | 8.7±1.5 | / | |  |  |
| Hällsten, 2002(82) | | 26 weeks | Rosiglitzone 4mg bid | 14 | 58.6±2.0 | 71.4 | 29.3±1.0 | / | 6.8±0.2 | / | |  |  |
|  | |  | PBO | 14 | 57.7±1.9 | 71.4 | 30.3±1.2 | / | 6.3±0.1 | / | |  |  |
| Scherbaum,2002(129) | | 26 weeks | Pioglitazone 30mg qd | 78 | 59.6 | 41 | 29.3 | 4.6 | 9.06 | 82.0 | |  |  |
|  | |  | PBO | 84 | 59.1 | 56 | 29.2 | 5.6 | 8.75 | 84.8 | |  |  |
| Viljanen, 2005(83) | | 26 weeks | Rosiglitazone 4mg bid | 14 | 58.6±7.7 | 64.3 | 29.3±4.0 | 6.8±0.9 | 6.8±0.9 | / | |  |  |
|  | |  | PBO | 11 | 58.7±8.3 | 81.8 | 29.8±4.1 | 6.2±0.7 | 6.2±0.7 | / | |  |  |
| Khan, 2006(130) | | 26 weeks | Pioglitazone 45mg | 23 | 56.7±7.17 | 60.9 | 30.7±3.05 | / | 9.54±0.326 | 90.3±13.06 | |  |  |
|  | |  | PBO | 21 | 54.8±8.65 | 28.6 | 32.0±4.23 | / | 8.62±0.323 | 89.3±11.25 | |  |  |
| Truitt, 2010(131) | | 26 weeks | Pioglitazone 45mg qd | 91 | 56.6±10.1 | 58.2 | 32.9±5.7 | 6.6±7.5 | 7.98±0.83 | 94.9±21.5 | |  |  |
|  | |  | PBO | 92 | 55.3±9.3 | 51.1 | 32.2±5.8 | 6.7±5.6 | 8.21±0.98 | 92.9±20.4 | |  |  |
| Chou, 2012(132) | | 26 weeks | Pioglitazone 45mg qd | 751 | 55.0±10.8 | 53 | 30.0±5.80 | 4.4±4.99 | 7.7±0.58 | 81.6±19.59 | |  |  |
|  | |  | PBO | 137 | 55.4±12.3 | 48.9 | 30.1±5.43 | 4.9±6.13 | 7.7±0.54 | 82±19.73 | |  |  |
| Ebeling, 2001(69) | | 6 months | Pioglitazone 30mg qd | 9 | / | / | 30.5±1.3 | / | 9.1±0.3 | / | |  |  |
|  | |  | PBO | 10 | / | / | 31.9±1.5 | / | 8.6±0.2 | / | |  |  |
| **DPP-4 inhibitor versus PBO** | | | | | | | | | | | |  |  |
| Ristic, 2005(133) | | 12 weeks | Vildagliptin 100mg qd | 61 | 56.2±10.1 | 55.6 | 31.1±4.01 | 3.03±4.22 | 7.64±0.75 | 91.5±2.2 | |  |  |
|  | |  | PBO | 55 | 54.6±10.6 | 56.9 | 31.6±4.41 | 2.28±2.99 | 7.76±0.83 | 92±2.1 | |  |  |
| Hanefeld, 2007(134) | | 12 weeks | Sitagliptin 100mg qd | 110 | 56.0±7.9 | 55.5 | 31.6±5.8 | 3.6±3.9 | 7.8±0.9 | / | |  |  |
|  | |  | PBO | 111 | 55.9±9.3 | 63.1 | 31.4±5.1 | 3.3±3.4 | 7.6±0.9 | / | |  |  |
| Scott, 2007(61) | | 12 weeks | Sitagliptin 50mg bid | 124 | 55.1±9.8 | 52.4 | 30.4±4.9 | 4.2±4.0 | 7.8±1.0 | / | |  |  |
|  | |  | PBO | 125 | 55.3±9.7 | 62.4 | 31.6±5.8 | 4.7±4.2 | 7.9±1.0 | / | |  |  |
| Nonaka, 2008(135) | | 12 weeks | Sitagliptin 100mg qd | 75 | 55.6±8.6 | 60 | 25.2±3.5 | 4.0±4.1 | 7.5±0.9 | / | |  |  |
|  | |  | PBO | 76 | 55.0±8.0 | 66 | 25.1±3.2 | 4.1±4.6 | 7.7±0.9 | / | |  |  |
| Rosenstock,2008(136) | | 12 weeks | Saxagliptin 5 mg qd | 47 | 53.7±10.14 | 53 | 30.8±4.21 | 0.8 | 7.9±1.09 | 89.8±15.92 | |  |  |
|  | |  | PBO | 67 | 55.2±9.8 | 63 | 31.1±4.46 | 1.8 | 8.0±0.88 | 93.1±19.21 | |  |  |
| Kikuchi, 2009(137) | | 12 weeks | Vildagliptin 50 mg | 76 | 58.8 ±8.6 | 67.1 | 24.3 ±2.8 | 4.7±4.3 | 7.4 ±0.8 | 62.7±9.3 | |  |  |
|  | |  | PBO | 72 | 60.4±8.1 | 63.9 | 24.6 ±3.1 | 7.1 ±5.5 | 7.4 ±0.8 | 63.8±10.1 | |  |  |
| Iwamoto, 2010(138) | | 12 weeks | PBO | 73 | 60.2±8.0 | 68.5 | 24.1±3.2 | 6.4±5.5 | 7.74±0.93 | / | |  |  |
|  | |  | Sitagliptin 100mg qd | 70 | 58.3±9.5 | 51.4 | 24.2±2.8 | 5.4±5.4 | 7.56±0.80 | / | |  |  |
| Pattzi, 2010(139) | | 12 weeks | Dutogliptin 200mg | 174 | 53±10 | 53.4 | 31.7±5.6 | / | 8.5±1.0 | / | |  |  |
|  | |  | PBO | 86 | 53±11 | 52.3 | 31.7±5.8 | / | 8.4±1.1 | / | |  |  |
| cRhee, 2010(140) | | 12 weeks | Gemigliptin 200mg | 35 | 54.29±10 | 51.43 | 24.97±3.26 | 3.18±3.25 | 8.16±1.13 | 67.27±11.47 | |  |  |
|  | |  | PBO | 34 | 51.26±8.6 | 67.65 | 25.56±2.93 | 4.31±4.90 | 8.20±1.21 | 70.56±11.97 | |  |  |
| Seino, 2011(141) | | 12 weeks | Alogliptin 25 mg qd | 80 | 59.5±11.2 | 78.8 | 24.70±4.15 | 6.98±6.99 | 7.88±0.99 | 66±13.34 | |  |  |
|  | |  | PBO | 75 | 59.1±10.5 | 74.7 | 24.39±3.69 | 6.83±6.07 | 7.85±0.89 | 64.57±12.01 | |  |  |
| Kawamori, 2012(142) | | 12 weeks | Linagliptin 5 mg | 159 | 60.3±9.4 | 69.8 | 24.6 ±4.0 | / | 8.07 ±0.66 | / | |  |  |
|  | |  | PBO | 80 | 59.7±8.9 | 71.3 | 24.3±3.4 | / | 7.95±0.67 | / | |  |  |
| Kadowaki, 2013(143) | | 12 weeks | Teneligliptin 40mg | 81 | 57.5±10.4 | 65.4 | 24.1±3.7 | 6.5±6.1 | 7.7±0.7 | / | |  |  |
|  | |  | PBO | 80 | 58.5±9.6 | 63.8 | 25.2±3.9 | 5.8±5.0 | 8.0±0.7 | / | |  |  |
| Inagaki, 2014(144) | | 12 weeks | SYR-472 100mg | 55 | 57.8±10.4 | 51 | 25.1±4.0 | 6.1±5.2 | 8·41±0·97 | / | |  |  |
|  | |  | PBO | 55 | 61.6±9.8 | 66 | 24.8±3.4 | 6.0±4.0 | 8·15±0·95 | / | |  |  |
| Sheu,2015(145) | | 12 weeks | Omarigliptin | 114 | 55.1±8.8 | 60.5 | 29.2±5.2 | 5.9±5.2 | 8.1±1.0 | 80.5±17.5 | |  |  |
|  | |  | PBO | 114 | 55.9±8.4 | 57.0 | 29.6±5.3 | 5.8±4.6 | 8.1±0.9 | 82.1±20.4 | |  |  |
| Yoon, 2017(146) | | 12 weeks | Gemigliptin | 64 | 61.7±7.9 | 59.4 | 26.0±3.7 | 16.7±9.0 | 8.32±0.9 | 68.62±10.50 | |  |  |
|  | |  | PBO | 60 | 62.3±9.0 | 57.6 | 26.5±4.3 | 15.9±8.7 | 8.43±1.0 | 69.06±11.17 | |  |  |
| Pan 2015([147](#_ENREF_88)) | | 16 weeks | Alogliptin 25mg qd | 88 | 51.4±10.4 | 58.9 | 25.8±3.1 | 1.9±2.4 | 8.05±0.92 |  | |  |  |
|  | |  | PBO | 88 | 53.2±9.0 | 57.1 | 25.8±3.0 | 2.0±2.5 | 7.86±0.79 |  | |  |  |
| Agarwal,2018(148) | | 16 weeks | Teneligliptin | 158 | 49.6±8.79 | 63.9 | / | / | 7.75 | 67.7±11.7 | |  |  |
|  | |  | PBO | 79 | 48.9±7.84 | 54.4 | / | / | 7.74 | 69.2±12.49 | |  |  |
| Raz, 2006(149) | | 18 weeks | Sitagliptin 100mg qd | 193 | / | / | / | / | 8.04±0.82 | / | |  |  |
|  | |  | PBO | 103 | / | / | / | / | 8.05±0.9 | / | |  |  |
| Mohan, 2009(150) | | 18 weeks | Sitagliptin 100mg qd | 352 | 50.9 ± 9.3 | 57 | 25.1± 3.4 | 2.1± 1.7 | 8.7 ± 1.0 | 66.8±10.2 | |  |  |
|  | |  | PBO | 178 | 50.9 ± 9.3 | 60 | 24.9 ± 3.4 | 1.9 ±1.6 | 8.8± 1.1 | 66.6±11.4 | |  |  |
| Aschner, 2006(151) | | 24 weeks | Sitagliptin 100mg qd | 229 | / | / | / | / | 8.01±0.88 | / | |  |  |
|  | |  | PBO | 244 | / | / | / | / | 8.03±0.82 | / | |  |  |
| Dejager, 2007(152) | | 24 weeks | Vildagliptin 100mg qd | 92 | 53.6±10.8 | 53.3 | 32.4±6.1 | 2.4±4.2 | 8.4±0.8 | / | |  |  |
|  | |  | PBO | 94 | 52.2±11.2 | 47.9 | 32.6±5.6 | 1.6±2.5 | 8.4±0.8 | / | |  |  |
| Goldstein, 2007(80) | | 24 weeks | Sitagliptin 100mg qd | 175 | / | / | / | / | 8.87±0.99 | / | |  |  |
|  | |  | PBO | 165 | / | / | / | / | 8.68±1.00 | / | |  |  |
| Pi-Sunyer, 2007(153) | | 24 weeks | Vildagliptin 50mg bid | 83 | 50.2±12.7 | 56.6 | 32.2±6.0 | 2.4±3.2 | 8.4±0.9 | 89.9±18.5 | |  |  |
|  | |  | PBO | 92 | 52.0±12.0 | 54.3 | 32.7±6.4 | 2.5±3.7 | 8.5±0.8 | 93±23.2 | |  |  |
| Rosenstock,2009(154) | | 24 weeks | Saxagliptin 5 mg qd | 106 | 53.91±12 | 50.9 | 32.24±4.5 | 2.5±3.3 | 8.0±1.1 | 90.87±18.15 | |  |  |
|  | |  | PBO | 95 | 53.91±12 | 49.5 | 30.93±4.26 | 2.3±2.7 | 7.9±0.9 | 86.56±16.9 | |  |  |
| Del Prato, 2011(155) | | 24 weeks | Linagliptin 5 mg | 333 | 56.4±10.1 | 48.8 | 29.04±4.80 | / | 8.0±0.05 | 78.53±16.73 | |  |  |
|  | |  | PBO | 163 | 54.4±10.3 | 47.3 | 29.08±4.84 | / | 8.0±0.07 | 79.21±15.95 | |  |  |
| Pan, 2012(156) | | 24 weeks | Saxagliptin 5mg qd | 284 | 51.2 ±10.0 | 56.3 | 25.9 ±3.4 | 0.8 ±1.4 | 8.1 ±0.8 | 69.2±11.4 | |  |  |
|  | |  | PBO | 284 | 51.6 ±10.3 | 54.6 | 25.9 ±3.7 | 1.2 ±2.6 | 8.2 ±0.8 | 69.2±12.4 | |  |  |
| Frederich, 2012(157) | | 24 weeks | Saxagliptin 5 mg qd | 74 | 54.7±9.71 | 51.4 | 31.0±5.23 | 1.7±2.4 | 8.0±0.9 | 86.5±20.71 | |  |  |
|  | |  | PBO | 74 | 55.6±10.3 | 47.3 | 31.1±4.54 | 1.7±2.8 | 7.8±1.0 | 85.4±14.4 | |  |  |
| Haak, 2012(81) | | 24 weeks | Linagliptin 5 mg | 142 | 56.2±10.8 | 56.3 | 29.0±4.7 | / | 8.7±1.0 | 79.1±17.3 | |  |  |
|  | |  | PBO | 72 | 55.7±11.0 | 50 | 28.6±5.2 | / | 8.7±1.0 | 76.8±15.0 | |  |  |
| Yang, 2012(158) | | 24 weeks | Gemigliptin 200mg | 87 | 54 | 57 | 25.4 | 3.24±3.84 | 8.2±1.0 | / | |  |  |
|  | |  | PBO | 87 | 52 | 44 | 26.7 | 2.86±4.36 | 8.3±1.1 | / | |  |  |
| Roden, 2013(159) | | 24 weeks | Sitagliptin 100mg qd | 223 | 55.1±9.9 | 63 | 28.2±5.2 | / | 7.85±0.79 | 79.3±20.4 | |  |  |
|  | |  | PBO | 228 | 54.9±10.9 | 54 | 28.7±6.2 | / | 7.91±0.78 | 78.2±19.9 | |  |  |
| Inagaki, 2015(160) | | 24 weeks | Trelagliptin | 101 | 58 | 72 | 25.4±4.42 | 6.25±5.94 | 7.73±0.85 | 69.23±14.27 | |  |  |
|  | |  | PBO | 50 | 62 | 86 | 24.6±4.27 | 7.54±5.50 | 7.72±0.77 | 67.20±14.72 | |  |  |
| Wu 2015(161) | | 24 weeks | Linagliptin 5mg qd | 34 | 52.5±11.0 | 65.7 | 24.37±2.09 | / | 7.97±0.68 |  | |  |  |
|  | |  | PBO | 23 | 51.2±7.5 | 50 | 24.11±2.28 | / | 8.00±0.69 |  | |  |  |
| Hong, 2016(162) | | 24 weeks | Teneligliptin | 99 | 56.64±10 | 52.53 | 24.96±2.51 | 4.59 | 7.63±0.69 | 65.81±11.39 | |  |  |
|  | |  | PBO | 43 | 57.93±12 | 65.12 | 25.07±3.23 | 4.59 | 7.77±0.81 | 66.61±12.15 | |  |  |
| Ji 2016(163) | | 24 weeks | Sitagliptin 100mg qd | 120 | 53.6±9.7 | 68.5 | 25.4±3.4 | 1.1±0.2 | 9.0±1.1 |  | |  |  |
|  | |  | PBO | 127 | 51.7±10.2 | 61.7 | 26.0±3.5 | 1.1±0.2 | 8.7±1.1 |  | |  |  |
| Chacra, 2017(164) | | 24 weeks | Omarigliptin | 107 | 65.9±9.4 | 63.6 | 29.5±4.5 | 14.9±8.2 | 8.3±0.8 | 80.0±16.3 | |  |  |
|  | |  | PBO | 106 | 64.5±9.7 | 59.4 | 30.7±6.8 | 15.1±8.7 | 8.3±0.8 | 84.2±20.4 | |  |  |
| Home, 2018(165) | | 24 weeks | Omarigliptin | 165 | 57.4±9.2 | 57.6 | 31.1±6.1 | 5.4±3.8 | 8.0±0.9 | 87.6±21.6 | |  |  |
|  | |  | PBO | 164 | 57.0±9.7 | 59.1 | 30.8±6.6 | 5.7±4.7 | 8.1±1.0 | 87.9±23.2 | |  |  |
| Gantz, 2017(166) | | 24 weeks | Omarigliptin | 166 | 60±11 | 62.7 | / | 7.4±5.5 | 7.9±0.7 | 67±13 | |  |  |
|  | |  | PBO | 83 | 61±9 | 68.7 | / | 8.6±5.1 | 8.1±0.7 | 64±12 | |  |  |
| Prately,2014(167) | | 26 weeks | Alogliptin | 112 | 52.6±9.38 | 42.9 | 30.8±5.22 | 3.6±4.12 | / | / | |  |  |
|  | |  | PBO | 109 | 53.1±9.6 | 50.5 | 31.2±5.27 | 4.3±4.78 | / | / | |  |  |
| Ji, 2017(168) | | 26 weeks | Alogliptin | 162 | 55.4±9.62 | 60.1 | 26.16±3.92 | / | 8.48±0.71 | / | |  |  |
|  | |  | PBO | 161 | 52.2±10.2 | 58.3 | 26.56±4.22 | / | 8.21±0.77 | / | |  |  |
| Scherbaum,2008(169) | | 52 weeks | Vildagliptin 50mg qd | 156 | 63.3±10.2 | 59.6 | 30.4±4.9 | 2.5±2.9 | 6.7±0.4 | / | |  |  |
|  | |  | PBO | 150 | 62.8±11.0 | 59.3 | 30.0±4.9 | 2.7±3.2 | 6.8±0.4 | / | |  |  |
| **SGLT2i versus PBO** | | | | | | | | | | | |  |  |
| Ott, 2017(170) | | 6 weeks | Dapagliflozin 10mg qd | 31 | / | / | 29.5±4.1 | / | 6.62±0.7 | / | |  |  |
|  | |  | PBO | 31 | / | / | 29.9±4.2 | / | 6.79±0.8 | / | |  |  |
| List, 2009(73) | | 12 weeks | Dapagliflozin 10mg qd | 47 | 54±9 | 53 | 31±5 | / | 8.0±0.8 | 86±17 | |  |  |
|  | |  | PBO | 54 | 53±11 | 56 | 32±5 | / | 7.9±0.9 | 89±18 | |  |  |
| Ferrannini, 2013(171) | | 12 weeks | Empagliflozin 25mg qd | 82 | 57 | 50 | 28.3 | / | 7.8±0.8 | 81.2±40 | |  |  |
|  | |  | PBO | 82 | 58 | 54.9 | 28.8 | / | 7.8±0.8 | 82.2±50 | |  |  |
| Fonseca, 2013(74) | | 12 weeks | Ipraliflozin 300mg qd | 68 | 54.2±10.7 | 54.4 | 30.7±5.0 | 4.48±4.91 | 7.90±0.67 | 86.7±19.6 | |  |  |
|  | |  | PBO | 69 | 53.4±9.7 | 46.4 | 30.9±5.5 | 4.64±5.93 | 7.84±0.78 | 81.8±17.4 | |  |  |
| Inagaki, 2013(172) | | 12 weeks | Canagliflozin 300mg | 75 | 57.1±10.1 | 73.3 | 25.89±3.68 | / | 8.17±0.81 | 71.3±12.19 | |  |  |
|  | |  | PBO | 75 | 57.7±11.0 | 72.0 | 26.41±4.34 | / | 7.99±0.77 | 72.56±15.36 | |  |  |
| Kaku, 2013(173) | | 12 weeks | Dapagliflozin 10mg | 52 | 56.5±11.5 | 75 | / | 4.73±4.73 | 8.18±0.69 | 70.35±17.48 | |  |  |
|  | |  | PBO | 54 | 58.4±10.0 | 79.6 | / | 4.74±3.82 | 8.12±0.71 | 68.88±14.94 | |  |  |
| Seino, 2014-2(174) | | 12 weeks | Luseogliflozin 5mg | 61 | 56.8±9.3 | 72.1 | 24.5±3.21 | 5.77±5.55 | 8.16±0.96 | 66.3±12.4 | |  |  |
|  | |  | PBO | 54 | 57.6±11 | 74.1 | 25.2±4.26 | 7.3±6.43 | 7.88±0.72 | 68.3±13.4 | |  |  |
| Seino, 2014-3(175) | | 12 weeks | Luseogliflozin 5mg | 54 | 57.3±11.4 | 75.9 | 26.43±4.26 | 4.5±4.2 | 7.86±0.69 | 72.56±13.94 | |  |  |
|  | |  | PBO | 57 | 57.1±10 | 71.9 | 25.15±3.62 | 5.1±4.6 | 7.92±0.84 | 67.32±13.14 | |  |  |
| Ikeda, 2015(176) | | 12 weeks | Tofogliflozin 20mg | 64 | 56.3±0.79 | 67.2 | 30.09±4.652 | 5.21±3.934 | 7.92±0.790 | 84.91±17.305 | |  |  |
|  | |  | PBO | 66 | 53.9±11.12 | 54.5 | 30.37±5.466 | 5.98±5.287 | 7.88±0.694 | 83.73±19.201 | |  |  |
| Sykes, 2015(177) | | 12 weeks | Remoglifflozin 500mg qd | 34 | 53.2±10.9 | 32 | 32.94±4.73 | / | 7.96±0.75 | 86.92±15.41 | |  |  |
|  | |  | PBO | 33 | 52.1±9.64 | 42 | 32.32±5.84 | / | 8.19±0.81 | 88.83±21.14 | |  |  |
| Sykes2015-1(178) | | 12 weeks | Remoglifflozin 500mg qd | 47 | 54.3±9.24 | 57 | 31.15±4.55 | 2.73±4.25 | 8.03±0.68 | 88.04±19.98 | |  |  |
|  | |  | PBO | 47 | 55.8±9.75 | 62 | 31.02±4.22 | 1.71±1.50 | 8.17±0.91 | 89.56±16.98 | |  |  |
| Heerspink,2016(179) | | 12 weeks | Dapagliflozin 10mg qd | 167 | 54.8±8.6 | 57.5 | 31.6±5.6 | 8.6±6.5 | 8.1±1.0 | / | |  |  |
|  | |  | PBO | 189 | 55.1±8.9 | 69.8 | 31.3±5.2 | 8.3±5.9 | 8.1±0.9 | / | |  |  |
| Ferrannini, 2010(180) | | 24 weeks | Dapagliflozin 10mg | 70 | 50.6±9.97 | 48.6 | 33.6±5.4 | 0.45 | 8.01±0.96 | 94.2±18.7 | |  |  |
|  | |  | PBO | 75 | 52.7±10.3 | 41.3 | 32.3±5.5 | 0.5 | 7.84±0.87 | 88.8±19 | |  |  |
| Bailey, 2012(181) | | 24 weeks | Dapagliflozin 5 mg qd | 68 | 51.3±11.5 | 47.1 | 30.97±5.68 | 1.4±3.24 | 7.9±1.03 | 85.4±19.43 | |  |  |
|  | |  | PBO | 68 | 53.5±11.1 | 54.4 | 32.47±4.91 | 1.1±1.95 | 7.8±1.12 | 90±17.98 | |  |  |
| Roden, 2013(159) | | 24 weeks | Empagliflozin 25mg | 224 | 53.8±11.6 | 65 | 28.2±5.5 | / | 7.86±0.85 | 77.8±18.0 | |  |  |
|  | |  | PBO | 228 | 54.9±10.9 | 54 | 28.7±6.2 | / | 7.91±0.78 | 78.2±19.9 | |  |  |
| Inagaki, 2014(182) | | 24 weeks | Canagliflozin 200mg | 88 | 57.4±11.1 | 81.8 | 25.43±4.18 | 5.88±5.93 | 8.04±0.77 | 69.88±14.22 | |  |  |
|  | |  | PBO | 93 | 58.2±11.0 | 64.5 | 25.85±4.39 | 5.63±5.76 | 8.04±0.70 | 68.57±15.15 | |  |  |
| Kaku, 2014(183) | | 24 weeks | Tofogliflozin 20mg | 58 | 56.6±10.2 | 67.2 | 24.99±4.55 | 6.4±5.1 | 8.34±0.81 | 68.06±15.82 | |  |  |
|  | |  | PBO | 56 | 56.8±9.9 | 66.1 | 26.00±4.11 | 6.0±6.1 | 8.41±0.78 | 71.20±12.64 | |  |  |
| Ji, 2014(184) | | 24 weeks | Dapagliflozin 10mg | 133 | 51.2±9.89 | 64.7 | 25.76±3.43 | 1.67±2.8 | 8.28±0.95 | 70.92±11.64 | |  |  |
|  | |  | PBO | 132 | 49.9±10.9 | 65.9 | 25.93±3.64 | 1.30±2.0 | 8.35±0.95 | 72.18±13.23 | |  |  |
| Kashiwagi, 2015(185) | | 24 weeks | Ipragliflozin 50mg | 118 | 63.9±6.59 | 78.0 | 25.84±3.450 | 9.5±7.7 | 7.53±0.538 | 69.16±11.571 | |  |  |
|  | |  | PBO | 46 | 65.7±6.93 | 78.3 | 24.96±3.362 | 9.4±8.3 | 7.55±0.526 | 66.70±10.940 | |  |  |
| Kashiwagi, 2015(186) | | 24 weeks | Ipragliflozin 50mg | 112 | 56.2±10.7 | 58.9 | 25.96±4.410 | 7.5±5.7 | 8.25±0.719 | 68.52±13.864 | |  |  |
|  | |  | PBO | 56 | 57.7±9.24 | 58.9 | 25.47±3.092 | 8.1±5.2 | 8.38±0.738 | 67.51±11.365 | |  |  |
| Seino, 2014-1 (187) | | 24 weeks | Luseogliflozin 2.5mg | 79 | 58.9±10.1 | 75.9 | 25.98±4.88 | 6.5±5.9 | 8.14±0.91 | 70.19±13.65 | |  |  |
|  | |  | PBO | 79 | 59.6±9.3 | 70.9 | 25.34±4.19 | 6.1±5.4 | 8.17±0.8 | 66.67±11.23 | |  |  |
| Seino, 2015(188) | | 24 weeks | Luseogliflozin 2.5mg | 150 | 61.2±8.4 | 74.7 | 24.78±3.63 | 7.4±5.6 | 8.07±0.85 | 66.39±11.48 | |  |  |
|  | |  | PBO | 71 | 59.9±10.5 | 67.6 | 24.66±3.31 | 7.9±6.6 | 8.01±0.73 | 65.34±10.57 | |  |  |
| Haneda, 2016(189) | | 24 weeks | Luseogliflozin 2.5mg | 95 | / | / | / | / | 7.72±0.68 | 66.9±13.6 | |  |  |
|  | |  | PBO | 50 | / | / | / | / | 7.69±0.65 | 67.77±11.79 | |  |  |
| Li, 2016(190) | | 24 weeks | Dapagliflozin | 18 | / | / | / | / | / | / | |  |  |
|  | |  | PBO | 10 | / | / | / | / | / | / | |  |  |
| Pollock, 2019[(191)](#_ENREF_132) | | 24weeks | Dapagliflozin | 145 | 64.7±8.6 | 30 | 30.19±5.3 | 17.55±7.7 | 8.44±1.0 | / | |  |  |
|  | |  | PBO | 148 | 64.7±8.5 | 71 | 30.34±5.6 | 17.71±9.5 | 8.57±1.2 | / | |  |  |
| Stenlöf, 2013(192) | | 26 weeks | Canagliflozin 300mg qd | 197 | 55.3±10.2 | 45.2 | 31.7±6.0 | 4.3±4.7 | 8.0±1.0 | 86.9±20.5 | |  |  |
|  | |  | PBO | 192 | 55.7±10.9 | 45.8 | 31.8±6.2 | 4.2±4.1 | 8.0±1.0 | 87.6±19.5 | |  |  |
| Terra,2017(193) | | 26 weeks | Ertugliflozin 15mg qd | 152 | 56.2±10.8 | 59.2 | 32.5±5.7 | 5.22±5.55 | 8.35±1.12 | 90.6±18.3 | |  |  |
|  | |  | PBO | 153 | 56.1±10.9 | 53.6 | 33.3±6.8 | 4.63±4.52 | 8.11±0.92 | 94.2±25.2 | |  |  |
| Aronson, 2018(194) | | 26 weeks | Ertugliflozin 15mg qd | 152 | 56.2±10.8 | 59.2 | / | / | 8.35±1.12 | 90.6±18.3 | |  |  |
|  | |  | PBO | 153 | 56.1±10.9 | 53.6 | / | / | 8.11±0.92 | 94.2±25.2 | |  |  |
| **Augmented PBO effect (add-on therapy)** | | | | | | | | | | | |  |  |
| **SU versus PBO** | |  |  |  |  |  |  |  |  |  | |  |  |
| Lins, 1988(195) | | 12 weeks | Glibenclamide 3.5mg bid +INS | 10 | 67±2 | 40 | / | / | 8.2±0.8 | / | |  |  |
|  |  |  | PBO+INS | 10 | 60±3 | 60 | / | / | 8.1±0.5 | / | |  |  |
| Stuart, 1997(196) | | 12 weeks | Glyburide 3mg bid+INS | 9 | / | / | / | / | 7.7±0.2 | 98±3.2 | |  |  |
|  |  |  | PBO+INS | 9 | / | / | / | / | 7.4±0.3 | 98±3.2 | |  |  |
| Forst, 2010(197) | | 12 weeks | Glimepiride 3mg qd | 65 | 59.4±9.9 | 63.1 | 31.5±4.2 | 6.7±5.9 | 8.2±0.7 | 90.5±15 | |  |  |
|  |  |  | PBO+MET | 71 | 60.1±8.1 | 62 | 32.2±4.2 | 6.2±5.1 | 8.4±0.7 | 93.1±16.8 | |  |  |
| Burant, 2012(198) | | 12 weeks | glimepiride 4mg qd | 62 | 52.2±9.7 | 55 | 31.0±5.4 | 6.4±5.7 | 8.43±0.81 | 83.5±17.8 | |  |  |
|  |  |  | PBO | 61 | 52.9±11.3 | 43 | 31.2±5.0 | 5.6±4.8 | 8.46±1.07 | 84.5±20.7 | |  |  |
| Schade, 1987(199) | | 4 months | Glyburide 20mg qd+INS | 8 | / | / | / | / | 10.6±0.4 | 84.6±4.4 | |  |  |
|  |  |  | PBO+INS | 8 | / | / | / | / |  |  | |  |  |
| Stenman, 1988(200) | | 4 months | Glibenclamide 15mg qd+INS | 8 | / | / | / | / | 9.2±0.2 | 74.7±3.0 | |  |  |
|  | |  | PBO+INS | 8 | / | / | / | / |  | 73.4±3.2 | |  |  |
| Riddle, 1992(201) | | 16 weeks | Glyburide 20mg qd+INS | 11 | / | / | / | / | 11.0±0.1 | / | |  |  |
|  |  |  | PBO+INS | 10 | / | / | / | / | 10.9±0.2 | / | |  |  |
| Feinglos, 2005(202) | | 16 weeks | Glipizide 2.5mg qd+MET | 61 | 57.7±10.7 | 45.9 | 31.7±4.4 | 6.5 | 7.45 | 90±18.7 | |  |  |
|  |  |  | PBO+MET | 61 | 58.8±10.0 | 41 | 32.1±4.9 | 4.6 | 7.64 | 90.8±18.4 | |  |  |
| Lewitt, 1989(203) | | 6 months | Glyburide15mg qd+INS | 31 | 67 | 87.1 | 26.7±2.9 | / | 9.9±1.3 | / | |  |  |
|  |  |  | PBO+INS | 31 | 67 | / | 26.7±2.9 | / | 9.9±1.3 | / | |  |  |
| Riddle, 1998(204) | | 24 weeks | Glimepiride 8mg bid+INS | 72 | 58±8 | 62.5 | 32.2±4.4 | 7±4 | 9.7±1.3 | 93.9±15.9 | |  |  |
|  |  |  | PBO+INS | 73 | 58±8 | 54.8 | 33.7±5.4 | 7±4 | 9.8±1.3 | 99.2±20.8 | |  |  |
| Roberts, 2005(205) | | 26 weeks | Glimepiride 8mg qd+MET+SU | 82 | 56.5±9.8 | 61 | 33.98±5.15 | 8.7±6.8 | 8.15±0.76 | 100.88±18.97 | |  |  |
|  |  |  | PBO+MET+SU | 77 | 56.4±10.0 | 62.3 | 32.76±5.11 | 7.9±4.9 | 8.15±0.65 | 96.31±19.1 | |  |  |
| Nauck, 2009(206) | | 26 weeks | Glimepiride 4mg qd+MET | 242 | 57±9 | 57 | 31.2±4.6 | 8±5 | 8.4±1.0 | / | |  |  |
|  |  |  | PBO+MET | 121 | 56±9 | 60 | 31.6±4.4 | 8±6 | 8.4±1.1 | / | |  |  |
| Karlander, 1991(207) | | 325 days | Glyburide 10.5mg qd+INS | 10 | / | / | / | / | 11.1±1.5  10.3±2.5 | 83.5±18.5 | |  |  |
|  |  |  | PBO+INS | 10 | / | / | / | / |  | 82.7±26.0 | |  |  |
| Camerini-Davalos, 1994(208) | | 3 years | Glipizide 5mg qd+INS | 40 | 45.2±1.7 | / | 25.1±0.2 | 10.5 | 11.2±0.3 | / | |  |  |
|  |  |  | PBO+INS | 29 | 46.4±2.0 | / | 24.3±0.4 | 14.1 | 10.3±0.3 | / | |  |  |
| **MET versus PBO** | |  |  |  |  |  |  |  |  |  | |  |  |
| Willms, 1999(209) | | 12 weeks | MET 850mg bid+SU | 29 | 53.4±8.2 | 48.1 | / | 9.3±6.9 | 10.6±1.4 | 88.6±17.7 | |  |  |
|  |  |  | PBO+ SU | 29 | 59.2±9.4 | 58.6 | / | 10.0±6.4 | 10.6±1.6 | 90.2±15.4 | |  |  |
| Avilés-Santa, 1999(210) | | 24 weeks | MET 2500mg qd+INS | 21 | 53.1±9.4 | 28.6 | / | 9.2±6.4 | 9.0±1.4 | 103.9±25.2 | |  |  |
|  |  |  | PBO+INS | 22 | 54.6±7.8 | 45.5 | / | 10.1±4.7 | 9.1±1.5 | 106.6±23.2 | |  |  |
| Chiasson, 2001(86) | | 36 weeks | MET 500mg tid | 83 | 57.9±8.6 | 73.5 | 30.7±5.1 | 7.5±7.4 | 8.2±0.9 | 89±17.8 | |  |  |
|  |  |  | PBO | 83 | 57.7±9.9 | 67.5 | 31.1±4.4 | 5.1±4.9 | 8.1±0.7 | 88.6±14.1 | |  |  |
| Hermann, 2001(211) | | 12 months | MET 850mg bid+INS | 16 | 56.9±10.2 | 43.8 | 33.6±3.5 | 13 | 9.1±1.3 | 96.4±16.5 | |  |  |
|  |  |  | PBO+INS | 19 | 58.1±9.7 | 63.2 | 32.6±3.8 | 13 | 8.7±1.0 | 94.2±9.4 | |  |  |
| Douek, 2005(212) | | 12 months | MET 2000mg qd+INS | 92 | 58±8.9 | 67.4 | 30.9±4.5 | 9±5.2 | 9.7±1.3 | 88.5±14.7 | |  |  |
|  |  |  | PBO+INS | 91 | 58±7.7 | 62.6 | 31.5±4.3 | 10±5.2 | 10.0±1.5 | 91.1±15.7 | |  |  |
| Gram, 2011(213) | | 2 years | MET 1000mg bid+NPH | 45 | 55.4±8.5 | 57.8 | 35.7±6.4 | 8.2±4.0 | 8.9±1.2 | 105.1±17.7 | |  |  |
|  | |  | PBO+NPH | 46 | 55.8±7.7 | 71.7 | 34.0±6.0 | 7.3±4.3 | 8.7±1.3 | 100.2±19.8 | |  |  |
| Gram, 2011(213) | | 2 years | MET 1000mg bid+ASP | 45 | 56.1±8.2 | 62.2 | 33.7±6.1 | 8.7±4.5 | 8.5±1.2 | 100.5±17.9 | |  |  |
|  | |  | PBO+ASP | 48 | 57.1±8.5 | 47.9 | 33.7±5.0 | 9.1±5.5 | 8.5±1.2 | 98.3±16.6 | |  |  |
| Kooy, 2009(214) | | 4.3 years | MET 850mg tid hydrochloride+INS | 196 | 64±10 | 41.3 | 30±5 | 14±9 | 7.9±1.2 | 85±16 | |  |  |
|  |  |  | PBO+INS | 194 | 59±11 | 50 | 30±5 | 12±8 | 7.9±1.2 | 87±15 | |  |  |
| **AGI versus PBO** | | | | | | | | | | | | |  |
| Willms, 1999(209) | | 12 weeks | Acarbose 100mg tid+SU | 31 | 60.3±8.8 | 48.4 | / | 10.4±6.8 | 10.6±1.3 | 86.±15.4 | |  |  |
|  |  |  | PBO+ SU | 29 | 59.2±9.4 | 58.6 | / | 10.0±6.4 | 10.6±1.6 | 90.2±15.4 | |  |  |
| Nemoto, 2011(215) | | 12 weeks | Miglitol 50mg tid+INS | 107 | / | / | / | / | 7.85±1.02 | / | |  |  |
|  |  |  | PBO+INS | 100 | / | / | / | / |  | / | |  |  |
| Hwu, 2003(216) | | 18 weeks | Acarbose 100mg tid+INS | 54 | 58.1±8.4 | / | / | 13.4±9.1 | 9.5±0.8 | 62±9.6 | |  |  |
|  |  |  | PBO+INS | 53 | 54.7±8.6 | / | / | 10.9±6.1 | 9.5±1 | 61.4±12.1 | |  |  |
| Schnell, 2007(217) | | 20 weeks | Acarbose 100mg tid+INS | 82 | 61.5±8.9 | / | 30.4±4.2 | 11.5±7 | 9.8±1.5 | 85.44±13.64 | |  |  |
|  |  |  | PBO+INS | 81 | 62.3±7.4 | / | 29.9±4.5 | 9.6±5.1 | 9.4±1 | 84.41±13.18 | |  |  |
| Kelley, 1998(218) | | 24 weeks | Acarbose 100mg tid+INS | 72 | 61.8 | 63 | 31 | 12.5 | 8.77 | 91.4 | |  |  |
|  |  |  | PBO+INS | 73 | 60.8 | 48 | 31.1 | 12.3 | 8.69 | 88.8 | |  |  |
| Lam KSL, 1998(219) | | 24 weeks | Acarbose 100mg tid+SU+MET | 45 | 57.8±1.3 | 44.4 | 24.8±0.5 | 10.2±0.7 | 9.5±0.1 | / | |  |  |
|  |  |  | PBO+SU+MET | 44 | 56.9±1.3 | 43.2 | 24.1±0.4 | 10.1±0.8 | 9.4±0.1 | / | |  |  |
| Mitrakou,1998(220) | | 24 weeks | Miglitol 100mg tid+INS | 60 | 57.4±5.6 | 48.3 | 24.4±3.1 | 8.5±4.5 | 9.9±0.5 | 67.3±6.2 | |  |  |
|  |  |  | PBO+ INS | 60 | 57.4±5.8 | 61.7 | 24.5±3.4 | 7.9±3.2 | 9.9±0.4 | 68.9±7.3 | |  |  |
| Standl, 1999(221) | | 24 weeks | Acarbose 200mg tid+INS | 24 | 59.3±8.5 | / | 25.2±2.2 | 11.5±6.8 | 10.9±1 | 70.5±11.7 | |  |  |
|  |  |  | PBO+INS | 24 | 62.9±9.4 | / | 24.1±2.0 | 12.2±5.7 | 11.0±1.2 | 66.7±10.8 | |  |  |
| Standl, 2001(222) | | 24 weeks | Miglitol 100mg tid+SU+MET | 65 | 62±8 | 50.8 | 27.7±3.7 | 8 | 8.83±0.85 | 79±13 | |  |  |
|  |  |  | PBO+ SU+MET | 68 | 61±8 | 54.4 | 27.9±3.5 | 9 | 8.84±0.66 | 82±15 | |  |  |
| Lin BJ, 2003(223) | | 24 weeks | Acarbose 100mg tid+SU | 32 | 57.7±7.3 | 53.1 | / | 7 | 9.01±1.2  8.99±0.95 | 63.9±7.2 | |  |  |
|  |  |  | PBO+SU | 32 | 55.4±8.5 | 37.5 | / | 5 |  | 61.6±7.9 | |  |  |
| Phillips, 2003(224) | | 24 weeks | Acarbose 100mg bid +MET | 40 | 58.4±10.7 | 65 | 30.75±2.96 | 5.32±4.55 | 8.05±0.89 | 89.77±12.73 | |  |  |
|  |  |  | PBO+MET | 43 | 62.4±8.02 | 76.7 | 30.09±2.85 | 6.06±5.32 | 7.82±0.83 | 87.88±11.7 | |  |  |
| Hsieh, 2011(225) | | 24 weeks | Miglitol 100mg tid+SU | 52 | 58.4±10.5 | 79.3 | / | / | 8.14±0.72 | 67.2±10.3 | |  |  |
|  |  |  | PBO+SU | 53 | 59±10.7 | 51.4 | / | / | 8.11±0.77 | 69.2±10.6 | |  |  |
| Halimi, 2000(226) | | 6 months | Acarbose 100mg tid +MET | 59 | 56±9.2 | 47.4 | 30.1±3.3 | 9.5±7.4 | 8.6±1.1 | / | |  |  |
|  |  |  | PBO+MET | 70 | 55±10 | 62.8 | 29.7±3.3 | 9±7.5 | 8.5±1.1 | / | |  |  |
| Van Gaal, 2001(227) | | 32 weeks | Miglitol 100mg tid+MET | 77 | 57.9±10 | 41.5 | 30.0±4.0 | 6 | 8.5±1 | / | |  |  |
|  |  |  | PBO+ MET | 75 | 57.9±8.5 | 49.3 | 29.7±3.9 | 6 | 8.4±1 | / | |  |  |
| Chiasson, 2001(86) | | 36 weeks | Miglitol100mg tid +MET | 76 | 58.9±7.9 | 77.6 | 29.5±3.8 | 6.1±5.5 | 8.3±0.8 | 91±15.5 | |  |  |
|  | |  | PBO+MET | 83 | 57.9±8.6 | 73.5 | 30.7±5.1 | 7.5±7.4 | 8.2±0.9 | 88.6±14.1 | |  |  |
| Chiasson-1, 1994(98) | | 1 year | Acarbose 200mg qd +MET | 83 | / | / | 29.4±0.6 | / | 7.8±0.2 | / | |  |  |
|  |  |  | PBO+MET | 83 | / | / | 29.4±0.6 | / |  | / | |  |  |
| Chiasson-2, 1994(98) | | 1 year | Acarbose 200mg qd +SU | 103 | / | / | 27.8±0.4 | / | 8±0.2 | / | |  |  |
|  |  |  | PBO+SU | 103 | / | / | 27.8±0.4 | / |  | / | |  |  |
| Chiasson-3, 1994(98) | | 1 year | Acarbose 200mg qd +INS | 91 | / | / | 30.2±0.5 | / | 7.7±0.2 | / | |  |  |
|  |  |  | PBO+INS | 91 | / | / | 30.2±0.5 | / |  | / | |  |  |
| Johnston, 1998 (228) | | 1 year | Miglitol 100mg tid+SU | 204 | 55±1 | 50 | 31.9±0.1 | 5.1±0.5 | 8.69±0.14 | / | |  |  |
|  |  |  | PBO+SU | 105 | 56.9±1.3 | 51 | 32.0±0.9 | 4.5±0.7 | 8.62±0.18 | / | |  |  |
| Johnston, 1998-2(229) | | 1 year | Miglitol 200mg tid+SU | 220 | 52.9 | / | 31.8 | 5.7 | 8.7 | 85.8 | |  |  |
|  |  |  | PBO+SU | 120 | 53.9 | / | 30.6 | 4.8 | 8.53 | 82.9 | |  |  |
| Bachmann, 2003(230) | | 78 weeks | Acarbose100mg tid +SU | 164 | 63.8±7.1 | 47.6 | 29.0±3.1 | 8±13.3 | 9.42±0.66 | 80.7±11.4 | |  |  |
|  |  |  | PBO+SU | 166 | 63.3±7.2 | 43.3 | 29.0±2.9 | 8±12.5 | 9.38±0.73 | 81.6±11.8 | |  |  |
| **TZD versus PBO** | | | | | | | | | | | | |  |
| Iwamoto, 1996(231) | | 12 weeks | Troglitazone 200mg bid+SU | 122 | 57.8±9.0 | 50.8 | 23.7±3.4 | / | 9.18±1.36 | 59.2±9.3 | |  |  |
|  |  |  | PBO+SU | 126 | 58.7±8.0 | 42.9 | 23.3±3.1 | / | 8.98±1.45 | 57.7±9.6 | |  |  |
| Kawamori, 1998(232) | | 12 weeks | Pioglitazone 30mg qd±SU | 21 | 57.6±8.5 | 66.7 | 23.0±1.8 | 12.5±9.1 | 8.4±1.4 | 61.1±6.6 | |  |  |
|  |  |  | PBO±SU | 9 | 60.6±10.0 | 55.6 | 22.0±3.0 | 11.9±8.1 | 8.7±1.3 | 57.1±14.2 | |  |  |
| Kelly, 1999(233) | | 12 weeks | Troglitazone 200mg tid±SU | 11 | 58.0±8.6 | 72.7 | 28.7±3.9 | / | 7.51±1.38 | 78.9±11.2 | |  |  |
|  |  |  | PBO±SU | 10 | 58.6±7.5 | 80 | 28.6±3.76 | / | 8.38±1.52 | 82.1±12.2 | |  |  |
| Pan, 2002(234) | | 12 weeks | Pioglitazone 30mg qd+SU+MET | 141 | / | / | / | / | 8.5±1.34 | / | |  |  |
|  |  |  | PBO+SU+MET | 142 | / | / | / | / | 8.5±1.12 | / | |  |  |
| Buras, 2005(235) | | 12 weeks | Troglitazone 600mg | 33 | 58±9 | 60.6 | 30.9±5.3 | 8±8 | 7.6±1.4 | / | |  |  |
|  |  |  | PBO | 39 | 57±9 | 66.7 | 32.6±5.0 | 8±9 | 7.9±1.4 | / | |  |  |
| Mimura, 1994(236) | | 3 months | CS 045 200mg bid | 8 | 53±2.7 | 50 | 22.4±0.7 | / | 9.3±0.4 | / | |  |  |
|  |  |  | PBO | 6 | 58±2.1 | 50 | 21.3±1.4 | / | 9.7±0.3 | / | |  |  |
| Osende, 2001(237) | | 3 months | Troglitazone 600mg qd | 19 | 57.2±1.8 | 68.4 | 30.4±1.9 | / | 9.1±0.3 | / | |  |  |
|  |  |  | PBO | 21 | 57.0±1.7 | 52.4 | 31.5±2.1 | / | 9.2±0.2 | / | |  |  |
| Brackenridge, 2009(238) | | 3 months | Pioglitazone 30mg+MET | 8 | 61.0±3.93 | 87.5 | 30.8±1.26 | 4.0±0.8 | 7.5±0.21 | 96.4±3.62 | |  |  |
|  |  |  | PBO+MET | 8 | 60.8±3.45 | 87.5 | 32.0±1.56 | 2.9±0.4 | 6.6±0.14 | 103.9±5.61 | |  |  |
| Buysschaert, 1999(239) | | 16 weeks | Troglitazone 200mg+SU | 90 | 60 | 66.7 | / | 6.43 | 7.9 | / | |  |  |
|  |  |  | PBO+SU | 85 | 60 | 51.8 | / | 7.77 | 8.5 | / | |  |  |
| Kipnes, 2001(240) | | 16 weeks | Pioglitazone 30mg qd+SU | 189 | 56.6±10.1 | 60 | 32.4±7.2 | / | 9.9±0.2 | / | |  |  |
|  |  |  | PBO+SU | 187 | 56.9±8.9 | 58 | 32.0±4.9 | / | 9.9±0.2 | / | |  |  |
| Scott, 2008(241) | | 18 weeks | Rosiglitazone 8mg qd+MET | 87 | 54.8±10.5 | 63 | 30.4±5.5 | 4.6±4 | 7.7±0.8 | 84.9±18.5 | |  |  |
|  | |  | PBO+MET | 92 | 55.3±9.3 | 59 | 30.0±4.5 | 5.4±3.7 | 7.7±0.9 | 84.6±16.5 | |  |  |
| Gastaldelli, 2007(122) | | 4 months | Pioglitazone 45mg qd+SU | 10 | 55±4 | 50 | 28.9±1.3 | 6±2 | 9.3±0.4 | / | |  |  |
|  | |  | PBO+SU | 10 | 55±4 | 40 | 29.9±1.4 | 5±2 | 8.3±0.4 | / | |  |  |
| Berhanu, 2007(242) | | 20 weeks | Pioglitazone 45mg qd+INS+MET | 110 | 52.9±11.3 | 43.6 | 30.7±6.09 | 7.7±6.15 | 8.4±0.13 | / | |  |  |
|  |  |  | PBO+INS+MET | 112 | 52.5±11.1 | 41.1 | 31.8±6.2 | 8.5±5.43 | 8.6±0.13 | / | |  |  |
| Yale, 2001(243) | | 24 weeks | Troglitazone 400mg qd+MET+SU | 101 | 58±0.9 | 55 | 30.1±0.5 | 11.9±0.8 | 9.6±0.1 | 85.2±1.6 | |  |  |
|  |  |  | PBO+MET+SU | 99 | 60±0.9 | 58 | 30.0±0.4 | 10.8±0.6 | 9.7±0.1 | 84.6±1.5 | |  |  |
| Zhu, 2003(244) | | 24 weeks | Rosiglitazone 4mg bid+SU | 210 | 58.9±6.9 | 48 | 24.9±3.1 | 7.9 | 9.8±1.5 | / | |  |  |
|  |  |  | PBO+SU | 105 | 58.8±7.7 | 46 | 25.1±2.8 | 7.6 | 9.8±1.3 | / | |  |  |
| Dailey, 2004(245) | | 24 weeks | Rosiglitazone 8mg qd+glyburide/MET | 181 | 57±9 | 58 | 32±5 | 9±7 | 8.1±0.9 | 93±18 | |  |  |
|  |  |  | PBO+glyburide/MET | 184 | 57±10 | 61 | 32±5 | 9±6 | 8.1±0.8 | 93±18 | |  |  |
| Smith, 2005(246) | | 24 weeks | Piogitazone 45mg qd | 21 | 56.2±9.7 | 42.9 | 32.1±5.6 | / | 6.88±1.35 | 93.5±19.6 | |  |  |
|  |  |  | PBO | 21 | 53.1±9.3 | 47.6 | 31.9±5.0 | / | 6.46±0.72 | 91.5±14.9 | |  |  |
| Davidson, 2007(247) | | 24 weeks | Rosiglitazone 8mg qd+glyburide | 117 | 52±11.9 | 45.3 | 31.3±5.7 | 6.0±5.2 | 9.2±1.3 | 86.3±18.8 | |  |  |
|  |  |  | PBO+ glyburide | 116 | 53±10.4 | 48.3 | 31.9±5.6 | 6.2±5.3 | 9.4±1.4 | 88.3±19.4 | |  |  |
| Hollander, 2007(248) | | 24 weeks | Rosiglitazone 4mg qd+INS | 189 | 52.6±10.1 | 48.1 | 33.7±7.1 | 13.0±7.3 | 9.0±1.2 | 96.8±20.8 | |  |  |
|  |  |  | PBO+INS | 186 | 53.8±10.2 | 46.2 | 33.0±6.5 | 12.6±8.6 | 9.1±1.3 | 95.1±20.9 | |  |  |
| Sridhar, 2013(249) | | 24 weeks | Pioglitazone 30mg qd+glimepide+MET | 25 | 47.9±5.8 | 100 | 25.3±2.7 | 2.2±1.7 | 6.8±0.4 | 70.4±11.4 | |  |  |
|  |  |  | PBO+glimepide+MET | 25 | 44.0±7.2 | 100 | 25.1±3.2 | 2.9±2.1 | 6.8±0.4 | 69.6±7.8 | |  |  |
| Buse, 1998(250) | | 26 weeks | Troglitazone 400mg | 76 | 58±10 | 50 | 34.8±6.7 | / | 9.0±1.4 | 98.9±20.2 | |  |  |
|  |  |  | PBO | 71 | 57±11 | 49 | 34.5±7.2 | / | 9.0±1.4 | 98.9±22.4 | |  |  |
| Schwartz, 1998(251) | | 26 weeks | Troglitazone 600mg qd+INS | 116 | 56±9 | 46 | 35.1±5.5 | 10±5 | 9.3±1.1 | 100.7±17.9 | |  |  |
|  |  |  | PBO+INS | 118 | 56±10 | 51 | 35.0±6.3 | 10±4 | 9.4±1.1 | 100．6±19.3 | |  |  |
| Fonseca, 2000(252) | | 26 weeks | Rosiglitazone 8mg qd+MET | 110 | 58.3±8.8 | 68.2 | 29.8±3.9 | 8.3±6.3 | 8.9±1.5 | / | |  |  |
|  | |  | PBO+MET | 113 | 58.8±9.2 | 74.3 | 30.3±4.4 | 7.3±5.7 | 8.6±1.3 | / | |  |  |
| Wolffenbuttel, 2000(253) | | 26 weeks | Rosiglitazone 2mg bid+SU | 183 | 60.6±8.7 | 55.2 | 28.3±3.9 | 7 | 9.23±1.18 | / | |  |  |
|  |  |  | PBO+SU | 192 | 61.9±9.1 | 57.3 | 28.1±4.1 | 8 | 9.21±1.30 | / | |  |  |
| Raskin, 2001(254) | | 26 weeks | Rosiglitazone 8mg qd+INS | 103 | 57.1±10.0 | 54.4 | 32.3±4.9 | 12.5±8.0 | 9.0±1.3 | / | |  |  |
|  |  |  | PBO+INS | 104 | 55.6±10.3 | 55.8 | 32.7±6.2 | 11.7±6.2 | 8.9±1.1 | / | |  |  |
| Barnett, 2003(255) | | 26 weeks | Rosiglitazone 8mg qd+SU | 84 | 54.3 | 80 | 26.8 | 6.5 | 9.21±1.27 | / | |  |  |
|  |  |  | PBO+SU | 87 | 54.1 | 75 | 26.4 | 6.5 | 9.06±1.3 | / | |  |  |
| Rosenstock, 2008(256) | | 26 weeks | Rosiglitazone 8mg qd+SU | 59 | 63±9 | 44 | 29.9±5.0 | 6.4±4.7 | 8.1±1.5 | 82.3±14.5 | |  |  |
|  |  |  | PBO+SU | 57 | 65±9 | 60 | 29.1±4.5 | 6.6±3.9 | 7.9±1.3 | 83.1±14.2 | |  |  |
| Marre, 2009(257) | | 26 weeks | PBO+SU | 114 | 54.7±10.0 | 47 | 30.3±5.4 | 6.5 | 8.4±1.0 | 81.9±17.1 | |  |  |
|  |  |  | Rosiglitazone 4mg qd+SU | 232 | 56.0±9.8 | 47 | 29.4±4.8 | 6.6 | 8.4±1.0 | 80.6±17 | |  |  |
| Henriksen, 2011(258) | | 26 weeks | Pioglitazone 45mg qd+INS | 102 | 60.1±8.6 | 69 | 33.2±5.0 | 13.8±7.4 | 8.7±1.4 | / | |  |  |
|  |  |  | PBO+INS | 106 | 60.9±7.8 | 62 | 33.9±5.5 | 12.6±7.3 | 8.5±1.3 | / | |  |  |
| Nakamura, 2001(259) | | 6 months | Pioglitazone 30mg qd | 14 | / | / | / | / | 8.4±1.3 | / | |  |  |
|  | |  | PBO | 14 | / | / | / | / | 8.0±1.0 | / | |  |  |
| Reynolds, 2002(260) | | 6 months | Rosiglitazone 4mg qd+INS | / | / | / | 36.4±1.8 | / | 8.0±0.3 | 109.6±9.2 | |  |  |
|  |  |  | PBO+INS | / | / | / | 36.3±1.8 | / | 9.8±0.5 | 106.4±6.4 | |  |  |
| Yang, 2002(261) | | 6 months | Rosiglitazone 2mg bid+SU | 30 | 58.9±9.4 | 43.3 | 25.76±2.87 | / | 9.5±1.1 | 64.9±11.8 | |  |  |
|  |  |  | PBO+SU | 34 | 57.8±8.9 | 38.2 | 25.84±3.50 | / | 9.7±1.4 | 65.3±11.2 | |  |  |
| Mattoo, 2005(262) | | 6 months | Pioglitazone 30mg qd+INS | 142 | 58.8±7.4 | 43.7 | 32.5±4.8 | 13.6±6.8 | 8.85±0.11 | / | |  |  |
|  |  |  | PBO+INS | 147 | 58.9±6.9 | 42.9 | 31.8±5.0 | 13.4±6.1 | 8.79±0.10 | / | |  |  |
| Derosa, 2008(263) | | 6 months | Rosiglitazone 8mg qd+MET | 56 | 55±4 | 46.4 | 28.6±1.9 | 3±1 | 7.8±0.7 | / | |  |  |
|  |  |  | PBO+MET | 61 | 54±3 | 47.5 | 28.4±1.7 | 4±1 | 8.0±0.9 | / | |  |  |
| Galle, 2012(264) | | 6 months | Pioglitazone 30mg+INS | 20 | 68.9±6.8 | 70 | 31.5±4.0 | 13.8±9.8 | 7.4±0.9 | / | |  |  |
|  |  |  | PBO+INS | 19 | 69.6±9.4 | 68.4 | 30.3±4.6 | 12.4±8.2 | 7.7±0.9 | / | |  |  |
| Grey, 2012(265) | | 6 months | Pioglitazone 30mg qd | 10 | 61.9±10.0 | 60 | 31.2±4.7 | / | 7.6±2.1 | 90.1±14.9 | |  |  |
|  |  |  | PBO | 10 | 57.9±15.2 | 50 | 33.2±4.1 | / | 7.1±1.0 | 91.7±12.8 | |  |  |
| Kaku, 2009(266) | | 28 weeks | Pioglitazone 30mg qd+MET | 83 | 52±8.6 | 66.3 | 25.6±4.2 | 4.5±3.7 | 7.58±1.0 | / | |  |  |
|  |  |  | PBO+MET | 86 | 53±7.5 | 57 | 25.4±3.6 | 5.6±5.0 | 7.55±0.9 | / | |  |  |
| Charpentier, 2009(267) | | 7 months | Pioglitazone 30/45mg | 142 | 60.2±9.3 | 64.6 | 29.1±3.3 | 12.5±9.0 | 8.1±0.7 | 82 | |  |  |
|  |  |  | PBO | 147 | 59.2±9.6 | 66.2 | 29.2±3.1 | 12.1±7.9 | 8.2±0.6 | 82.5 | |  |  |
| Negro, 2005(268) | | 12 months | Rosiglitazone 4mg bid+MET | 19 | 60.3±6.4 | 52.6 | 28.3±1.7 | 7.1±2.4 | 8.4±0.6 | 84.1±4.6 | |  |  |
|  |  |  | PBO+MET | 19 | 59±8 | 63.2 | 28.7±1.9 | 6.6±2.9 | 8.1±0.5 | 83.6±4.4 | |  |  |
| Bertrand, 2010(269) | | 12 months | Rosiglitazone 4mg bid | 98 | 64.2±7.3 | 92 | 30.2±4.2 | 7.8±6.4 | 6.9±1.3 | 85.9±14 | |  |  |
|  |  |  | PBO | 95 | 65.9±6.9 | 92 | 29.5±4.6 | 8.4±6.9 | 6.9±0.8 | 83.9±15.1 | |  |  |
| Gram, 2011(213) | | 2 years | Rosiglitazone+NPH | 46 | 57.3±8.9 | 60.9 | 34.0±5.7 | 9.2±6.9 | 8.7±1.2 | 100.9±16.5 | |  |  |
|  | |  | PBO+NPH | 46 | 55.8±7.7 | 71.7 | 34.0±6.0 | 7.3±4.3 | 8.7±1.3 | 100.2±19.8 | |  |  |
| Gram, 2011(213) | | 2 years | Rosiglitazone+ASP | 47 | 56.1±8.3 | 57.4 | 32.7±4.7 | 9.4±6.3 | 8.3±1.0 | 95.6±14.6 | |  |  |
|  | |  | PBO+ASP | 48 | 57.1±8.5 | 47.9 | 33.7±5.0 | 9.1±5.5 | 8.5±1.2 | 98.3±16.6 | |  |  |
| **DPP-4 inhibitor versus PBO** | | | | | | | | | | | | |  |
| Ahrén, 2004(270) | | 12 weeks | LAF237 50mg qd+MET | 56 | 57.9 ±10.0 | 69.6 | 29.4± 3.6 | 5.6 ± 4.2 | 7.7 ± 0.6 | / | |  |  |
|  |  |  | PBO+MET | 51 | 55.7 ±11.0 | 66.7 | 30.2 ± 3.6 | 5.5± 3.7 | 7.8 ± 0.7 | / | |  |  |
| Forst, 2010(197) | | 12 weeks | Linagliptin 5mg+MET | 66 | 59.6±9.8 | 56.1 | 31.7±4.5 | 7.3±7.5 | 8.5±0.8 | 90.7±14.2 | |  |  |
|  |  |  | PBO+MET | 71 | 60.1±8.1 | 62 | 32.2±4.2 | 6.2±5.1 | 8.4±0.7 | 93.1±16.8 | |  |  |
| Kikuchi, 2010(271) | | 12 weeks | Vildagliptin 50mg bid+SU | 102 | 59.2±9.8 | 73.5 | 24.5±2.9 | 8.6±6.7 | 7.8±0.8 | 65±0.95 | |  |  |
|  |  |  | PBO+SU | 100 | 60.3±10.1 | 69 | 24.4±2.6 | 9.8±6.4 | 8±0.8 | 63.6±0.96 | |  |  |
| Kaku, 2011(272) | | 12 weeks | Alogliptin 25 mg+ PIO | 113 | 59.3 ±10.7 | 70/43 | 26.07 ±3.7 | 6.80 ±5.7 | 7.89 ±0.73 | 68.07±13 | |  |  |
|  | |  | PBO+PIO | 115 | 60.1 ±9.7 | 76/39 | 26.4 ±4.4 | 6.7 ±5.3 | 7.92 ±0.85 | 69±14.4 | |  |  |
| Nowicki, 2011(273) | | 12 weeks | Saxagliptin2.5mg qd +INS/OAD | 85 | 66.8±8.3 | 37.6 | 31.2±6.1 | 15.1±7.5 | 8.5±1.2 | 83.6±15.7 | |  |  |
|  |  |  | PBO+INS/OAD | 85 | 66.2±9.1 | 48.2 | 30.2±6.8 | 18.2±8.5 | 8.1±1.1 | 82.2±14.4 | |  |  |
| Seino, 2011(274) | | 12 weeks | Alogliptin25 mg+voglibose | 79 | 62.9 ±9.0 | 50/29 | 23.26 ±2.88 | 8.44 ±5.90 | 7.91±0.91 | 60.4±10.0 | |  |  |
|  | |  | PBO+voglibose | 75 | 62.3 ±10.5 | 48/27 | 24.42 ±4.20 | 7.52 ±6.03 | 8.12±1.19 | 64.4±10.5 | |  |  |
| Rosenstock, 2012(275) | | 12 weeks | Sitagliptin 100mg qd+MET | 65 | 51.7±8.1 | 58 | 31.6±5.0 | 5.6±4.7 | 7.64±0.95 | 87.2±18 | |  |  |
|  |  |  | PBO+MET | 65 | 53.3±7.8 | 48 | 30.6±4.6 | 6.4±5.0 | 7.75±0.83 | 85.9±19.5 | |  |  |
| Ross, 2012(276) | | 12 weeks | Linagliptin 5mg+ MET | 221 | 58.4±10.6 | 54 | 29.6±5.0 | / | 7.98±0.72 | 80.6±17.5 | |  |  |
|  |  |  | PBO+MET | 43 | 59.9±10.7 | 47.7 | 28.7±5.5 | / | 7.92±0.74 | 77.7±19.4 | |  |  |
| Kadowaki, 2013(277) | | 12 weeks | Teneligliptin+PIO | 103 | 59.7±9.7 | 66 | 26.2±5.2 | 7.2±4.8 | 8.1±0.9 | 70.0±16.6 | |  |  |
|  |  |  | PBO+PIO | 101 | 61.1±8.9 | 75.2 | 25.6±3.7 | 7.7±6.1 | 7.9±0.8 | 67.7±13.0 | |  |  |
| Kadowaki, 2014(278) | | 12 weeks | Teneligliptin 20mg qd+SU | 96 | 58.4±8.6 | 64.6 | 24.9±3.6 | 9.3±6.7 | 8.4±0.8 | 66.2±12.1 | |  |  |
|  | |  | PBO+SU | 98 | 60.3±7.8 | 67.3 | 24.6±3.6 | 8.3±6.2 | 8.4±0.8 | 65.9±11.8 | |  |  |
| Kim, 2014(279) | | 16 weeks | Teneligliptin+MET | 136 | 55.7±8.7 | 55.1 | / | 6.7±4.8 | 7.9±0.7 | / | |  |  |
|  |  |  | PBO+ MET | 68 | 56.4±9.2 | 50.0 | / | 8.0±5.9 | 7.8±0.6 | / | |  |  |
| Kadowaki, 2017(280) | | 16 weeks | Teneligliptin+ INS | 77 | 60.1±12.0 | 76.6 | 24.87±3.14 | 11.85±8.14 | 8.70±0.81 | 67.62±12.98 | |  |  |
|  |  |  | PBO +INS | 71 | 57.4±11.3 | 74.6 | 25.11±3.66 | 12.97±9.22 | 8.73±0.81 | 68.61±13.68 | |  |  |
| Pan, 2015(147) | | 16 weeks | Alogliptin 25mg +MET | 91 | 52.6±9.8 | 53.3 | 25.7±3.1 | 5.3±4.2 | 8.05±0.83 | / | |  |  |
|  |  |  | PBO+MET | 93 | 53.4±9.4 | 48.9 | 25.5±3.9 | 5.5±3.9 | 7.98±0.75 | / | |  |  |
| Pan, 2015(147) | | 16 weeks | Alogliptin 25mg +PIO | 60 | 52.6±9.4 | 45.9 | 25.3±3.2 | 5.8±5.3 | 7.94±0.79 | / | |  |  |
|  |  |  | PBO+PIO | 63 | 51.8±10.4 | 62.9 | 26.1±3.0 | 4.9±4.7 | 7.96±0.82 | / | |  |  |
| Raz, 2008(281) | | 18 weeks | Sitagliptin 100mg qd+MET | 96 | 53.6±9.5 | 51 | 30.1±4.4 | 8.4±6.5 | 9.3±0.9 | 81.5±16.8 | |  |  |
|  |  |  | PBO+MET | 94 | 56.1±9.5 | 41.5 | 30.4±5.3 | 7.3±5.3 | 9.1±0.8 | 81.2±19.4 | |  |  |
| Scott, 2008(241) | | 18 weeks | Sitagliptin 100mg qd+MET | 94 | 55.2±9.8 | 55 | 30.3±4.7 | 4.9±3.5 | 7.8±1.0 | 83.1±17.1 | |  |  |
|  |  |  | PBO+MET | 92 | 55.3±9.3 | 59 | 30.0±4.5 | 5.4±3.7 | 7.7±0.9 | 84.6±16.5 | |  |  |
| Lewin, 2012(282) | | 18 weeks | Linagliptin 5mg+SU | 161 | 57.2±9.8 | 47.8 | 28.4±5.0 | / | 8.6±0.9 | / | |  |  |
|  |  |  | PBO+SU | 84 | 56.2±10.2 | 61.9 | 28.2±5.1 | / | 8.6±0.7 | / | |  |  |
| Dobs, 2013(283) | | 18 weeks | Sitagliptin 100mg qd+MET+ROSI | 170 | 54.4±8.8 | 56 | 30.1±6.2 | 9.3±5.9 | 8.8±1.0 | 82.5±19.7 | |  |  |
|  |  |  | PBO+MET+ROSI | 92 | 54.8±9.5 | 60 | 30.8±5.6 | 9.4±6.8 | 8.7±1.0 | 86.8±20 | |  |  |
| 229.Gantz, 2017(284) | | 18 weeks | Omarigliptin+multiple anti-diabetes agents | 2100 | 63.7±8.5 | 69.6 | 31.2±5.5 | 12.0±7.6 | 8.0±0.9 | 89.0±18.5 | |  |  |
|  |  |  | PBO+multiple anti-diabetes agents | 2102 | 63.6±8.5 | 70.7 | 31.4±5.6 | 12.1±8.0 | 8.0±0.9 | 89.6±18.8 | |  |  |
| Charbonnel, 2006(285) | | 24 weeks | Sitagliptin 100mg qd+MET | 454 | / | / | / | / | 7.96±0.81 | / | |  |  |
|  |  |  | PBO+MET | 226 | / | / | / | / | 8.03±0.82 | / | |  |  |
| Rosenstock, 2006(286) | | 24 weeks | Sitagliptin 100mg qd+PIO | 175 | 55.6±10.4 | 53.1 | 32.0±5.2 | 6.1±5.4 | 8.1±0.8 | 90.9±17 | |  |  |
|  |  |  | PBO+PIO | 178 | 56.9±11.1 | 57.9 | 31.0±5.0 | 6.1±5.7 | 8.0±0.8 | 86.4±17.4 | |  |  |
| Bosi, 2007(287) | | 24 weeks | Vildagliptin 100mg+MET | 143 | 53.9±9.5 | 61.5 | 32.9±5.0 | 5.8±4.7 | 8.4±1.0 | / | |  |  |
|  |  |  | PBO+MET | 130 | 54.5±10.3 | 53.1 | 33.2±6.1 | 6.2±5.3 | 8.3±0.9 | / | |  |  |
| Fonseca, 2007(288) | | 24 weeks | Vildagliptin 50mg bid+INS | 144 | 59.6±10.3 | 47.9 | 33.3±5.2 | 14.4±8.6 | 8.4±1.0 | / | |  |  |
|  |  |  | PBO+INS | 152 | 58.9±10.8 | 54.6 | 32.9±5.9 | 14.9±8.4 | 8.4±1.1 | / | |  |  |
| Garber, 2007(289) | | 24 weeks | Vildagliptin 100mg+PIO | 136 | 54.0±9.2 | 44.9 | 32.2±5.8 | 4.6±4.8 | 8.7±1.2 | / | |  |  |
|  |  |  | PBO+PIO | 138 | 54.8±10.6 | 50.7 | 32.3±5.8 | 4.8±4.6 | 8.7±1.2 | / | |  |  |
| Hermansen, 2007(290) | | 24 weeks | Sitagliptin 100mg qd+SU+MET | 222 | 55.6±9.6 | 52.7 | 31.2±6.3 | 8.3±5.5 | 8.34±0.76 | 86.5±21.1 | |  |  |
|  |  |  | PBO+ SU+MET | 219 | 56.5±9.6 | 53.4 | 30.7±6.3 | 9.3±6.8 | 8.34±0.74 | 85.9±21.8 | |  |  |
| Garber, 2008(291) | | 24 weeks | Vildagliptin 100mg+SU | 132 | 58.2±11.1 | 59.8 | 30.8±5.3 | 6.7±5.3 | 8.6±1.0 | / | |  |  |
|  |  |  | PBO+SU | 144 | 57.9±10.5 | 58.3 | 31.0±5.5 | 7.8±5.8 | 8.5±1.0 | / | |  |  |
| Goodman, 2009(292) | | 24 weeks | Vildagliptin 100mg qd+MET | 248 | 54.9±10.8 | 52.8 | 31.4±4.7 | / | 8.5±1 | / | |  |  |
|  |  |  | PBO+MET | 122 | 54.5±9.7 | 67.2 | 31.7±4.3 | / | 8.7±1.1 | / | |  |  |
| Hollander, 2009(293) | | 24 weeks | Saxagliptin 5mg+TZD | 186 | 53.2±10.6 | 47.8 | 29.8±5.3 | 5.2±5.6 | 8.4±1.1 | 80.4±19.4 | |  |  |
|  |  |  | PBO+TZD | 184 | 54.0±10.1 | 46.2 | 30.3±5.8 | 5.1±5.4 | 8.2±1.1 | 80.9±21.5 | |  |  |
| JadzINSky, 2009(294) | | 24 weeks | Saxagliptin 5mg+MET | 320 | 52.0±10.4 | 51.6 | 29.9±4.5 | 2.0±3.6 | 9.4±1.2 | 82.1±16.3 | |  |  |
|  |  |  | PBO+MET | 328 | 51.8±10.7 | 49.7 | 30.2±4.9 | 1.7±3.1 | 9.4±1.3 | 82.8±17.5 | |  |  |
| Pratley, 2009(295) | | 24 weeks | Alogliptin 25mg+PIO | 199 | 55.4±10.2 | 62.8 | 33.1±5.4 | / | 8.0±0.8 | / | |  |  |
|  |  |  | PBO+PIO | 97 | 55.2±10.8 | 54.6 | 33.2±6.2 | / | 8.0±0.8 | / | |  |  |
| Vilsbøll, 2010(296) | | 24 weeks | Sitagliptin 100mg qd+INS | 322 | 58.3±9.1 | 49 | 31±5 | 13±7 | 8.7±0.9 | 86.5±18.6 | |  |  |
|  |  |  | PBO+INS | 319 | 57.2±9.3 | 53 | 31±5 | 12±6 | 8.6±0.9 | 87.3±17.9 | |  |  |
| Gomis, 2011(297) | | 24 weeks | Linagliptin 5mg+PIO | 259 | 57.7±9.6 | 58.7 | 28.7±4.8 | / | 8.6±0.05 | 78.3±15.6 | |  |  |
|  |  |  | PBO+PIO | 130 | 57.1±10.1 | 65.4 | 29.7±4.8 | / | 8.58±0.08 | 82.7±15.8 | |  |  |
| Owens, 2011(298) | | 24 weeks | Linagliptin 5 mg+MET+SU | 792 | 58.3±9.9 | 46.8 | 28.4±4.8 | / | 8.15±0.03 | 76.5±16.8 | |  |  |
|  |  |  | PBO+MET+SU | 263 | 57.6±9.7 | 48.3 | 28.2±4.5 | / | 8.14±0.05 | 76.8±16.8 | |  |  |
| Taskinen, 2011(299) | | 24 weeks | PBO+MET | 177 | 56.6±10.9 | 57 | 30.05±5.01 | / | 8.02±0.88 | 83.3±16.6 | |  |  |
|  |  |  | Linagliptin 5mg qd+MET | 523 | 56.5±10.1 | 53 | 29.85±4.84 | / | 8.09±0.86 | 82.2±17.2 | |  |  |
| Yang, 2011(300) | | 24 weeks | Saxagliptin 5mg qd+MET | 283 | 53.8 ±10.4 | 48.1 | 26.3 ±3.6 | 5.1 ±5.0 | 7.9 ±0.8 | 68.9±12.5 | |  |  |
|  |  |  | PBO+MET | 287 | 54.4 ±10.1 | 48.7 | 26.1 ±3.5 | 5.1 ±4.0 | 7.9±0.8 | 69±11.9 | |  |  |
| Barnett, 2012(301) | | 24 weeks | Saxagliptin 5mg qd+INS+MET | 304 | 57.2±9.43 | 40 | 32.6±5.65 | 11.8±6.93 | 8.7±0.9 | 87.7±18.57 | |  |  |
|  |  |  | PBO+INS+MET | 151 | 57.3±9.27 | 45 | 31.8±4.76 | 12.2±7.37 | 8.6±0.86 | 86.2±16.54 | |  |  |
| Bergenstal, 2012(302) | | 24 weeks | Sitagliptin 100mg+MET | 177 | 55.5±9.9 | 59 | 32.4±5 | 6.0±5.0 | 7.94±0.85 | 92.5±19.7 | |  |  |
|  | |  | PBO+MET | 90 | 56.1±10.1 | 52 | 32.5±5.5 | 5.5±3.9 | 8.03±0.83 | 91.1±19.0 | |  |  |
| Haak, 2012(81) | | 24 weeks | Linagliptin 2.5mg+MET | 143 | 55.6±11.2 | 51 | 29.7±5.3 | / | 8.7±1.0 | 80.8±19 | |  |  |
|  | |  | PBO+MET | 144 | 52.9±10.4 | 56.9 | 28.9±4.8 | / | 8.7±0.9 | 79.9±18.4 | |  |  |
| Haak, 2012(81) | | 24 weeks | Linagliptin 2.5mg+MET | 143 | 56.4±10.7 | 53.8 | 28.6±4.8 | / | 8.7±1.0 | 76.7±16 | |  |  |
|  | |  | PBO+MET | 147 | 55.2±10.6 | 53.1 | 29.5±5.3 | / | 8.5±0.9 | 80±18.5 | |  |  |
| Pan, 2012(303) | | 24 weeks | Vildagliptin 50mg bid+ MET | 146 | 54.2±9.62 | 50 | 26.01±3.26 | 4.92±4.8 | 8.09±0.85 | 71.58±11.93 | |  |  |
|  |  |  | PBO+MET | 144 | 54.5±9.68 | 45.8 | 25.46±3.09 | 5.15±4.58 | 8.01±0.82 | 69.83±11.18 | |  |  |
| Seino, 2012(304) | | 24 weeks | Alogliptin 25mg +MET | 96 | 52.3 ±8.02 | 68.8 | 25.79 ±3.70 | 6.62 ±4.80 | 8.02 ±0.73 | 69.65±12.67 | |  |  |
|  |  |  | PBO+MET | 100 | 52.1 ±8.05 | 72 | 26.14 ±4.58 | 6.04 ±4.36 | 8.00 ±0.86 | 69.89±14.23 | |  |  |
| Yang, 2012(305) | | 24 weeks | Sitagliptin 100mg qd+MET | 197 | 54.1±9.0 | 47 | 25.3±3.1 | 6.4±4.4 | 8.5±0.9 | 67.9±10.7 | |  |  |
|  |  |  | PBO+MET | 198 | 55.1±9.8 | 55 | 25.3±3.6 | 7.3±4.6 | 8.5±0.9 | 68.9±13.3 | |  |  |
| Barnett, 2013(306) | | 24 weeks | Linagliptin 5mg qd | 162 | 74.9±4.4 | 71.6 | 29.6±4.7 | / | 7.8±0.8 | 86.3±16.4 | |  |  |
|  |  |  | PBO | 79 | 74.9±4.2 | 62.0 | 29.8±4.5 | / | 7.7±0.7 | 84.4±15.3 | |  |  |
| Strain, 2013(307) | | 24 weeks | Vildagliptin 50mg bid | 139 | 75.1±4.3 | 52.5 | 29.1±3.8 | 12.2±7.9 | 7.9±0.8 | 79.9±1 | |  |  |
|  |  |  | PBO | 139 | 74.4±4 | 38.1 | 30.5±4.8 | 10.6±6.9 | 7.9±0.7 | 80.7±1 | |  |  |
| Zeng, 2013(308) | | 24 weeks | Linagliptin 5mg qd +MET+SU | 144 | 55.6±9.5 | 52.1 | 25.9±3.2 | / | 8.14±0.85 | 70.2±11.2 | |  |  |
|  |  |  | PBO+MET+SU | 48 | 57.0±8.9 | 52.1 | 25.6±3.4 | / | 8.10±0.84 | 71.4±13.8 | |  |  |
| Lukashevic, 2014(309) | | 24 weeks | Vildagliptin 50mg bid+MET+SU | 158 | 55.3±10.2 | 50.6 | 27.9±4.6 | 7.1±6.2 | 8.7±0.9 | 73.1 | |  |  |
|  |  |  | PBO+MET+SU | 160 | 55.0±11.1 | 45% | 28.0±4.5 | 7.5±6.1 | 8.8±0.9 | 72.4 | |  |  |
| Wang 2015(310) | | 24 weeks | Linagliptin 5mg qd +MET | 184 | 55.1±10.7 | 49.8 | 25.5±3.9 | / | 7.99±0.83 | 68.1±12.9 | |  |  |
|  |  |  | PBO+MET | 80 | 56.5±8.7 | 50 | 25.8±4.0 |  | 8.00±0.80 | 68.5±12.7 | |  |  |
| Yang, 2015(311) | | 24 weeks | Vildagliptin50mg qd+SU | 143 | 58.3±9.8 | 55.2 | 24.8±3.0 | 6.9±4.6 | 8.6±0.9 | 67.4±11.1 | |  |  |
|  |  |  | PBO+SU | 136 | 58.7±9.3 | 58.1 | 25.0±2.8 | 6.9±4.1 | 8.7±1.0 | 68.8±11.7 | |  |  |
| Ning, 2016(312) | | 24 weeks | Vildagliptin 50mg bid +INS | 117 | 58.2±8.74 | 47 | 25.8±2.88 | 10.8±6.71 | 8.6±0.84 | / | |  |  |
|  |  |  | PBO+INS | 118 | 58.5±9.33 | 46.6 | 25.7±2.68 | 11.4±6.53 | 8.6±0.93 | / | |  |  |
| Ahn, 2017(313) | | 24 weeks | Gemigliptin+MET+  SU | 107 | 61.4±9.2 | 37.4 | 25.1±3.1 | 13.0±6.8 | 8.2±0.8 | 63.7±10.5 | |  |  |
|  |  |  | PBO+MET+SU | 109 | 60.4±8.8 | 42.2 | 24.7±2.7 | 12.8±6.4 | 8.2±0.9 | 63.6±10.2 | |  |  |
| Lee,2017(314) | | 24 weeks | Omarigliptin+MET+SU | 154 | 57.2±8.4 | 47.4 | 31.8±6.2 | 9.8±5.3 | 8.5±0.8 | 87.8±18.3 | |  |  |
|  |  |  | PBO+MET+SU | 153 | 58.4±9.4 | 48.4 | 30.6±5.8 | 10.4±5.5 | 8.6±0.8 | 85.4±21.2 | |  |  |
| Shankar,2017(315) | | 24 weeks | Omarigliptin+MET | 201 | 57.5±8.1 | 50.2 | 32.4±5.8 | 8.2±5.2 | 8.1±0.9 | 90.7±18.7 | |  |  |
|  |  |  | PBO+MET | 201 | 56.8±9.1 | 50.7 | 32.6±6.1 | 7.4±5.6 | 8.0±0.9 | 91.2±20.8 | |  |  |
| Kadowaki, 2018(316) | | 24 weeks | Teneligliptin+ Canagliflozin | 77 | 55.9±8.3 | 83.1 | 25.53±3.95 | 8.15±5.86 | 7.98±0.80 | 72.32±12.08 | |  |  |
|  |  |  | PBO+Canagliflozin | 77 | 54.1±10.2 | 75.3 | 26.50±4.82 | 7.34±5.34 | 8.09±0.85 | 73.58±15.75 | |  |  |
| Nauck, 2009(317) | | 26 weeks | Alogliptin 25mg+ MET | 210 | 54±11 | 54.3 | 32±5 | 6±4 | 7.9±0.8 | / | |  |  |
|  | |  | PBO+MET | 104 | 56±11 | 48 | 32±6 | 6±5 | 8.0±0.9 | / | |  |  |
| DeFronzo, 2012(318) | | 26 weeks | Alogliptin 25mg+MET | 129 | 53.7±9.3 | 38.8 | 31.5±5.7 | 5.6±4.9 | 8.6±0.7 | / | |  |  |
|  | |  | PBO+MET | 129 | 55.2±9.9 | 47.3 | 30.6±4.8 | 6.0±5.0 | 8.5±0.6 | / | |  |  |
| Fonseca, 2013(319) | | 26 weeks | Sitagliptin100mg+MET+ PIO | 157 | 55.7±8.7 | 61.8 | 29.9±5.2 | 9.4±5.8 | 8.8±1.0 | 82.1±19.1 | |  |  |
|  | |  | PBO+MET+PIO | 156 | 56.4±9.4 | 62.8 | 30.0±5.2 | 10.2±6.1 | 8.7±1.0 | 83.8±19.1 | |  |  |
| Lavalle-González, 2013(320) | | 26 weeks | PBO+MET | 183 | 55.3±9.8 | 51.4 | 31.1±6.1 | 6.8±5.3 | 8.0±0.9 | 86.6±22.4 | |  |  |
|  |  |  | Sitagliptin 100mg+MET | 366 | 55.3±9.8 | 47 | 32.0±6.1 | 6.8±5.2 | 7.9±0.9 | 87.7±21.6 | |  |  |
| Ji, 2017(168) | | 26 weeks | Alogliptin 25mg +MET | 158 | / | / | 23.89±3.446 | / | 8.39±0.81 | / | |  |  |
|  | |  | PBO+MET | 161 | / | / | 24.81±2.871 | / | 8.40±0.77 | / | |  |  |
| Derosa, 2012(321) | | 12 months | Sitagliptin 100mg qd+MET | 91 | 55.9±8.8 | 46 | 28.1±1.2 | 5.8±2.6 | 8.1±0.8 | 78.4±6.6 | |  |  |
|  |  |  | PBO+MET | 87 | 54.8±7.9 | 51 | 28.9±2.0 | 5.4±2.3 | 8.0±0.7 | 78.6±6.7 | |  |  |
| Derosa, 2012(322) | | 12 months | Vildagliptin 50mg bid+ MET | 84 | 54.2±8.3 | 50 | 27.9±1.5 | 6.1±3.7 | 8.1±0.6 | 76.9±5.8 | |  |  |
|  |  |  | PBO+ MET | 83 | 52.4±7.1 | 51.8 | 27.8±1.4 | 6.3±3.9 | 8.2±0.7 | 78.5±6.4 | |  |  |
| Bosi, 2011(323) | | 52 weeks | Alogliptin 25mg+ MET+PIO | 404 | 54.3±9.86 | 52.0 | 31.5±5.25 | 7.5±5.24 | 8.2±0.86 | 88.2±18.9 | |  |  |
|  |  |  | PBO+MET+PIO | 399 | 55.9±9.94 | 51.1 | 31.6±5.18 | 6.9±4.61 | 8.1±0.83 | 88±19.28 | |  |  |
| Kothny, 2012(324) | | 52 weeks | Vildagliptin 50mg qd | 122 | 67.1±9.0 | 57.4 | 30.3±5.2 | / | 7.9±1.0 | / | |  |  |
|  |  |  | PBO | 89 | 69.3±7.2 | 61.8 | 30.1±5.0 | / | 7.9±1.0 | / | |  |  |
| Kothny, 2012(324) | | 52 weeks | Vildagliptin | 94 | 63.7±9.1 | 52.1 | 30.8±5.8 | / | 7.7±1.0 | / | |  |  |
|  |  |  | PBO | 64 | 65.4±10.5 | 51.6 | 30.0±4.7 | / | 7.5±1.1 | / | |  |  |
| **SGLT2i versus PBO** | | | | | | | | | | | | |  |
| Henry,2018(325) | | 4 weeks | Dapagliflozin+INS/MET | 50 | 56.9±7.1 | 52 | 34.3±5.9 | 10.5±6.0 | 8.31±0.79 | 96.5±23.5 | |  |  |
|  | |  | PBO+INS/MET | 50 | 56.8±6.9 | 50 | 33.2±5.6 | 12.3±7.4 | 8.37±0.81 | 96.0±20.0 | |  |  |
| Latva-Rasku, 2019(326) | | 8 weeks | Dapagliflozin+INS | 15 | 62±8.4 | 86.6 | 32.1±3.9 | 7.8±3.8 | 7.0±0.6 | / | |  |  |
|  | |  | PBO+INS | 16 | 60±7.4 | 75 | 31.7±5.0 | 7.3±3.7 | 6.8±0.5 | / | |  |  |
| Wilding, 2009(327) | | 12 weeks | Dapagliflozin 10mg+INS | 24 | 55.7±9.2 | 54.2 | 35.5±3.6 | 11.8±5.8 | 8.4±0.7 | 103.4±10.2 | |  |  |
|  |  |  | PBO+INS | 23 | 58.4±6.5 | 69.6 | 34.8±4.6 | 13.8±7.3 | 8.4±0.9 | 101.8±16.5 | |  |  |
| Rosenstock, 2012(275) | | 12 weeks | Canagliflozin 300mg qd+MET | 64 | 52.3±6.9 | 56 | 31.6±4.9 | 5.9±5.2 | 7.69±1.02 | 87.3±15.9 | |  |  |
|  |  |  | PBO+MET | 65 | 53.3±7.8 | 48 | 30.6±4.6 | 6.4±5.0 | 7.75±0.83 | 85.9±19.5 | |  |  |
| Wilding, 2013(328) | | 12 weeks | Ipragliflozin 300mg qd+MET | 72 | 56.6±8.9 | 50 | 31.8±4.6 | 5.5±4.8 | 7.87±0.82 | 89.3±15 | |  |  |
|  |  |  | PBO+MET | 66 | 57.3±8.6 | 54.5 | 32.0±4.8 | 5.7±3.2 | 7.68±0.60 | 89±14.5 | |  |  |
| Amin, 2015(329) | | 12 weeks | Ertugliflozin 25mg+MET | 55 | 54.2±8.8 | 32.7 | 29.8±0.67 | 6.0 | 8.30±0.16 | / | |  |  |
|  |  |  | PBO+MET | 54 | 54±8.1 | 44.4 | 30.6±0.61 | 6.4 | 8.08±0.14 | / | |  |  |
| Weber,2016(330) | | 12 weeks | Dapagliflozin 10mg qd+OAD/INS | 225 | 56.0 | 52 | / | 7.7±5.9 | 8.1±0.9 | 88.0±20.5 | |  |  |
|  |  |  | PBO+OAD/INS | 224 | 57 | 58 | / | 7.3±5.0 | 8.0±1.0 | 89.9±18.4 | |  |  |
| Kario,2018(331) | | 12weeks | Empagliflozin 10mg+OAD | 68 | 70.9±8.7 | 52.9 | 26.1±3.8 | 10.6±7.9 | 6.6±0.8 | 65.4±11.4 | |  |  |
|  | |  | PBO+OAD | 63 | 69.3±7.8 | 52.4 | 26.0±4.9 | 9.6±8.2 | 6.6±0.8 | 64.6±14.3 | |  |  |
| Ross, 2015(332) | | 16 weeks | Empagliflozin12.5 mg bid+MET | 215 | / | / | / | / | 7.78±0.05 | 89.42±1.30 | |  |  |
|  |  |  | PBO+MET | 207 | / | / | / | / | 7.69±0.07 | 90.10±1.78 | |  |  |
| Ross, 2015(332) | | 16 weeks | Empagliflozin 25mg qd+MET | 214 | / | / | / | / | 7.73±0.05 | 88.72±1.27 | |  |  |
|  |  |  | PBO+MET | 207 | / | / | / | / | 7.69±0.07 | 90.10±1.78 | |  |  |
| Schumm-Draeger,2015(333) | | 16 weeks | Dapagliflozin 5mg bid | 99 | 55.3±9.3 | 46.5 | 33.09±4.94 | 5.12±4.2 | 7.78±0.76 | 93.62±16.641 | |  |  |
|  |  |  | PBO | 101 | 58.5±9.4 | 46.5 | 31.74±4.69 | 5.53±4.23 | 7.94±0.85 | 88.82±15.327 | |  |  |
| Schumm-Draeger,2015(333) | | 16 weeks | Dapagliflozin 10mg qd | 99 | 58.5±9.8 | 49.5 | 32.25±5.01 | 5.45±4.05 | 7.71±0.71 | 90.58±15.929 | |  |  |
|  |  |  | PBO | 101 | 58.5±9.4 | 46.5 | 31.74±4.69 | 5.53±4.23 | 7.94±0.85 | 88.82±15.327 | |  |  |
| Ishihara, 2016(334) | | 16 weeks | Ipragliflozin+INS | 168 | 58.7±11.1 | 62.5 | 25.61±3.53 | 12.5 | 8.67±0.77 | 69.05±11.61 | |  |  |
|  |  |  | PBO+INS | 87 | 59.2±9.3 | 58.6 | 26.42±3.81 | 14.2 | 8.62±0.86 | 70.32±12.17 | |  |  |
| Terauchi, 2017(335) | | 16 weeks | Tofogliflozin 20mg qd+INS | 141 | 59.1±10.8 | 63.8 | 25.8±3.5 | 15.02±9.36 | 8.53±0.75 | 68.87±13.20 | |  |  |
|  |  |  | PBO+INS | 70 | 56.4±10.0 | 68.6 | 26.9±3.9 | 12.39±7.34 | 8.40±0.65 | 72.24±11.12 | |  |  |
| Seino, 2018(336) | | 16weeks | Luseogliflozin+INS | 159 | 57.4±10.3 | 70.4 | 25.42±3.53 | 11.7±7.6 | 8.70±0.83 | 68.10±11.32 | |  |  |
|  | |  | PBO+INS | 74 | 57.1±10.9 | 68.9 | 25.15±3.44 | 12.1±6.8 | 8.84±0.83 | 69.13±12.16 | |  |  |
| Ji,2015(337) | | 18 weeks | Canagliflozin 300mg | 227 | 56.4±9.2 | 49.8 | 26±3.4 | 6.9±4.9 | 8±0.9 | 69.6±11.9 | |  |  |
|  |  |  | PBO | 226 | 55.8±9.4 | 55.6 | 25.5±3.6 | 6.4±4.6 | 7.9±0.9 | 68.6±11.9 | |  |  |
| Neal, 2015(338) | | 18 weeks | Canagliflozin 300mg+INS | 660 | 63 | 65 | 33.3±6.2 | 16.3±7.4 | 8.3±0.9 | 94.8±21.3 | |  |  |
|  |  |  | PBO+INS | 636 | 63 | 66 | 33.1±6.5 | 16.0±7.8 | 8.3±0.9 | 94.8±22.3 | |  |  |
| Bailey, 2010(339) | | 24 weeks | Dapagliflozin 10 mg+MET | 135 | 52·7±9.9 | 57 | 31·2±5·1 | 6.1±5.4 | 7.92±0.82 | 86.3±11.5 | |  |  |
|  | |  | PBO+MET | 137 | 53.7±10.3 | 55 | 31·8±5·3 | 5.8±5.1 | 8.11±0.96 | 87.7±19.2 | |  |  |
| Strojek, 2011(340) | | 24 weeks | Dapagliflozin 10mg qd+SU | 151 | 58.9±8.32 | 43.7 | / | 7.2±5.5 | 8.07±0.79 | 80.56±0.65 | |  |  |
|  |  |  | PBO+SU | 145 | 60.3±10.2 | 49 | / | 7.4±5.7 | 8.15±0.74 | 80.94±1 | |  |  |
| Bolinder, 2012(341) | | 24 weeks | Dapagliflozin 10mg qd+MET | 89 | 60.6±8.2 | 58 | 32.1±3.9 | 6.0±4.5 | 7.19±0.44 | 92.1±14.1 | |  |  |
|  | |  | PBO+MET | 91 | 60.8±6.9 | 59.2 | 31.7±3.9 | 5.5±5.3 | 7.16±0.53 | 90.9±13.7 | |  |  |
| Henry, 2012(342) | | 24 weeks | Dapagliflozin 5mg qd+MET | 194 | 51.7±9.3 | 40.2 | / | 1.6±2.4 | 9.2±1.3 | 84.1±19.5 | |  |  |
|  |  |  | PBO+MET | 201 | 51.8±9.8 | 47.3 | / | 1.6±2.6 | 9.2±1.3 | 85.6±20 | |  |  |
| Henry, 2012(342) | | 24 weeks | Dapagliflozin 10mg qd+MET | 211 | 51.0±10.1 | 50.2 | / | 2.2±3.3 | 9.1±1.3 | 88.4±19.7 | |  |  |
|  |  |  | PBO+MET | 208 | 52.7±10.4 | 46.6 | / | 1.9±4.0 | 9.1±1.3 | 87.2±19.4 | |  |  |
| Häring2013(343) | | 24 weeks | Empagliflozin 25mg+MET+SU | 216 | 57.4±9.3 | 53 | 28.3±5.5 | / | 8.10±0.83 | 77.5±18.8 | |  |  |
|  |  |  | PBO+MET+SU | 225 | 56.9±9.2 | 50 | 27.9±4.9 | / | 8.15±0.83 | 76.2±16.9 | |  |  |
| Häring, 2014(344) | | 24 weeks | Empagliflozin 25mg+MET | 213 | 55.6±10.2 | 56 | 29.7±5.7 | / | 7.86±0.87 | 82.2±19.3 | |  |  |
|  | |  | PBO+MET | 207 | 56.0±9.7 | 56 | 28.7±5.2 | / | 7.90±0.88 | 79.7±18.6 | |  |  |
| Kovacs, 2014(345) | | 24 weeks | Empagliflozin 25mg+PIO+MET | 168 | 54.2±8.9 | 50.6 | 29.1±5.5 | / | 8.1±0.82 | 78.9±19.9 | |  |  |
|  | |  | PBO+PIO+MET | 165 | 54.6 ±10.5 | 44.2 | 29.3±5.4 | / | 8.2±0.92 | 78.1±20.1 | |  |  |
| Leiter, 2014(346) | | 24 weeks | Dapagliflozin 10mg | 480 | 63.9±7.6 | 66.9 | 33.0±5.3 | 13.5±8.2 | 8.0±0.8 | 94.5±17.8 | |  |  |
|  |  |  | PBO | 482 | 63.6±7.0 | 67.0 | 32.7±5.7 | 13.0±8.4 | 8.1±0.8 | 93.2±16.8 | |  |  |
| Mathieu, 2015(347) | | 24 weeks | Dapagliflozin 10mg+MET | 160 | 55.26±8.6 | 43.7 | 31.26±4.7 | 7.26±5.7 | 8.246±0.96 | 85.8±18.4 | |  |  |
|  |  |  | PBO+MET | 160 | 55.06±9.6 | 47.5 | 32.26±5.3 | 8.06±6.6 | 8.176±0.98 | 88.2±18.1 | |  |  |
| Matthaei,2015(348) | | 24 weeks | Dapagliflozin 10mg qd | 108 | 61.1±9.7 | 42.6 | 31.9±4.8 | 9.3±6.5 | 8.08±0.91 | 88.6±17.6 | |  |  |
|  |  |  | PBO | 108 | 60.9±9.2 | 55.6 | 32±4.6 | 9.6±6.2 | 8.24±0.87 | 90.1±16.2 | |  |  |
| Rosenstock, 2015(349) | | 24 weeks | Saxagliptin 5mg qd+Dapagliflozin 10 mg qd+MET | 179 | 53±10 | 47 | 31.8±4.8 | 7.1±5 | 8.92±1.18 | 87.1±18 | |  |  |
|  |  |  | Saxagliptin 5mg qd+PBO+MET | 176 | 55±10 | 53 | 31.8±5.1 | 8.2±5.5 | 9.03±1.05 | 88±18.7 | |  |  |
| Hadjadj, 2016(350) | | 24 weeks | Empagliflozin 12.5mg bid +MET 1000mg bid | 169 | 53.6±10.7 | 52.1 | 30.4±5.3 | / | 8.66±1.14 | 83.8±19.8 | |  |  |
|  |  |  | MET 1000mg bid | 164 | 51.6±10.8 | 56.1 | 30.5±5.9 | / | 8.58±1.13 | 83.7±20.1 | |  |  |
| Hadjadj, 2016(350) | | 24 weeks | Empagliflozin12.5mg bid+MET 500mg bid | 165 | 51.0±10.7 | 63.6 | 30.2±5.2 | / | 8.84±1.31 | 82.9±8.7 | |  |  |
|  |  |  | MET 500mg bid | 168 | 53.4±10.9 | 51.2 | 30.3±5.8 | / | 8.69±1.04 | 82.7±21.2 | |  |  |
| Lu, 2016(351) | | 24 weeks | Ipragliflozin 50mg qd+MET | 87 | 53.9 | 50.6 | 26.6 | 6.49 | 7.74±0.78 | 70.36±14.75 | |  |  |
|  |  |  | PBO+MET | 83 | 53.4 | 39.8 | 27.04 | 5.82 | 7.75±0.71 | 70.45±12.44 | |  |  |
| Kadowaki，2017(352) | | 24 weeks | Canagliflozin 100mg qd+teneligliptin 20mg qd | 70 | 58.4±8.9 | 77.1 | 25.53±4.21 | 8.34±7.74 | 8.18±0.9 | 71.33±15.94 | |  |  |
|  |  |  | PBO+teneligliptin 20mg qd | 68 | 56.0±9.5 | 77.9 | 26.44±3.87 | 6.50±3.89 | 7.87±0.83 | 73.26±12.91 | |  |  |
| Cusi, 2018(353) | | 24 weeks | Canagliflozin 300mg+MET | 26 | 58±9 | 62 | 32.2±4.1 | / | 7.6±0.8 | 94.5±12.8 | |  |  |
|  | |  | PBO+MET | 30 | 58±10 | 70 | 31.0±4.9 | / | 7.7±0.7 | 92.5±14.9 | |  |  |
| Fioretto, 2018(354) | | 24 weeks | Dapagliflozin+OAD | 160 | 65.3 | 56.9 | 32.6±4.7 | 14.3±8.1 | 8.33±1.08 | 92.4±16.8 | |  |  |
|  | |  | PBO+OAD | 161 | 66.2 | 56.5 | 31.6±5.0 | 14.5±8.3 | 8.03±1.08 | 88.3±16.2 | |  |  |
| Han,2018(355) | | 24 weeks | Ipragliflozin 50mg qd+MET | 73 | 57.6±8.26 | 50.7 | 25.0±30.7 | 11.61±5.89 | 7.9±0.69 | 67.5±12.5 | |  |  |
|  | |  | PBO+MET | 66 | 57.4±7.88 | 48.5 | 26.05±3.79 | 11.33±6.62 | 7.92±0.79 | 67.9±10.98 | |  |  |
| Shestakova, 2018(356) | | 24 weeks | Ipragliflozin 50mg qd+MET | 110 | 58.9±9.3 | 43.6 | 32.8±4.76 | 6.65±5.21 | 8.39±0.93 | 92.74±16.24 | |  |  |
|  | |  | PBO+MET | 55 | 58.0±9.5 | 40.0 | 31.95±4.18 | 4.65±6.55 | 8.46±0.96 | 89.54±15.6 | |  |  |
| Yang, 2018(357) | | 24 weeks | Dapagliflozin 10mg qd+INS | 139 | 56.5±8.4 | 47.5 | 26.4±3.8 | 12.7±7.2 | 8.52±0.76 | 71.1±12.0 | |  |  |
|  | |  | PBO+INS | 133 | 58.6±8.9 | 48.1 | 26.7±3.3 | 12.2±6.7 | 8.58±0.81 | 72.4±13.1 | |  |  |
| Ferdinand, 2019(358) | | 24 weeks | Empagliflozin10-25mg+OAD | 78 | 56.5±9.3 | 55.1 | 36.4±12.83 | 9.3±6.2 | 8.66±0.11 | 105±2.75 | |  |  |
|  | |  | PBO+OAD | 72 | 57.2±9.3 | 50.0 | 35.12±8.29 | 9.3±7.9 | 8.51±0.13 | 101.35±2.47 | |  |  |
| Pollock, 2019(191) | | 24 weeks | Dapagliflozin | 145 | 64.7±8.6 | 30 | 30.19±5.3 | 17.55±7.7 | 8.44±1.0 | / | |  |  |
|  | |  | PBO | 148 | 64.7±8.5 | 71 | 30.34±5.6 | 17.71±9.5 | 8.57±1.2 | / | |  |  |
| Lavalle-González, 2013(320) | | 26 weeks | Canagliflozin 300mg+MET | 367 | 55.4±9.4 | 45 | 31.4±6.3 | 7.1±5.4 | 7.9±0.9 | 85.4±20.9 | |  |  |
|  | |  | PBO+MET | 183 | 55.3±9.8 | 51.4 | 31.1±6.1 | 6.8±5.3 | 8.0±0.9 | 86.6±22.4 | |  |  |
| Yale, 2013(359) | | 26 weeks | Canagliflozin 300mg qd | 89 | 67.9±8.2 | 53.9 | 33.4±6.5 | 17.0±7.8 | 8.0±0.8 | 90.2±18.1 | |  |  |
|  |  |  | PBO | 90 | 68.2±8.4 | 63.3 | 33.1±6.5 | 16.4±10.1 | 8.0±0.9 | 92.8±17.4 | |  |  |
| Forst, 2014(360) | | 26 weeks | Canagliflozin 300mg+MET+PIO | 114 | 57.0±10.2 | 55.3 | 32.8±7.7 | 11.0±7.6 | 7.9±0.9 | 94.4±25.9 | |  |  |
|  | |  | PBO+MET+PIO | 115 | 58.3±9.6 | 66.1 | 32.5±6.4 | 10.1±6.6 | 8.0±1.0 | 93.8±22.4 | |  |  |
| Grunberger, 2018(361) | | 26 weeks | Ertugliflozin 5mg qd  +INS±SU | 158 | 66.7±8.3 | 53.2 | 32.6±6.8 | 14.9±9.0 | 8.2±1.0 | 89.4±22.5 | |  |  |
|  |  |  | Ertugliflozin 15mg qd+INS±SU | 155 | 67.5±8.5 | 48.4 | 31.7±5.3 | 14.5±8.5 | 8.2±0.9 | 85.8±17.4 | |  |  |
|  |  |  | PBO+INS±SU | 154 | 67.5±8.9 | 46.8 | 33.2±6.1 | 13.1±8.1 | 8.1±0.9 | 90.4±18.9 | |  |  |
| Miller, 2018(362) | | 26 weeks | Ertugliflozin 15mg+sitagliptin | 96 | 56.1±10.1 | 55.2 | 32.1±5.8 | 6.5±6.5 | 9.0±0.9 | 91.2±22.5 | |  |  |
|  | |  | PBO+sitagliptin | 97 | 54.3±10.3 | 58.8 | 32.7±6.2 | 6.3±6.05 | 9.0±0.9 | 95.0±20.5 | |  |  |
| Rosenstock, 2018(363) | | 26 weeks | Ertugliflozin 15mg qd +MET | 205 | 56.9±9.4 | 45.4 | 31.1±4.5 | 8.1±5.5 | 8.1±0.9 | 85.3±16.5 | |  |  |
|  | |  | PBO+MET | 209 | 56.5±8.7 | 46.9 | 30.7±4.7 | 8.0±6.3 | 8.2±0.9 | 84.5±17.1 | |  |  |
| Ji, 2019(364) | | 26 weeks | Ertugliflozin15mg+MET | 169 | 56.3±9.3 | 58.0 | 25.7±3.2 | 7.5±5.1 | 8.1±0.9 | 69.5±10.9 | |  |  |
|  | |  | PBO+MET | 167 | 56.9±9.0 | 52.7 | 26.1±3.4 | 6.4±5.1 | 8.1±1.0 | 70.1±12.4 | |  |  |
| Rosenstock, 2012(365) | | 48 weeks | Dapagliflozin 10mg+PIO | 140 | 53.8±10.4 | 42.1 | / | 5.75±6.44 | 8.37±0.96 | 84.8±22.2 | |  |  |
|  |  |  | PBO+PIO | 139 | 53.5±11.4 | 51.1 | / | 5.07±5.05 | 8.34±1.00 | 86.4±21.3 | |  |  |
| Wilding, 2012(366) | | 48 weeks | Dapagliflozin 10mg+INS | 194 | 59.3±8.8 | 44.8 | 33.4±5.1 | 14.2±7.3 | 8.57±0.82 | 94.5±16.8 | |  |  |
|  |  |  | PBO+INS | 193 | 58.8±8.6 | 49.2 | 33.1±5.9 | 13.5±7.3 | 8.47±0.77 | 94.5±19.8 | |  |  |
| Ljunggren, 2012(367) | | 52 weeks | Dapagliflozin 10mg qd+MET | 89 | 60.6±8.2 | 55.1 | 32.1±3.9 | 6.0±4.5 | 7.19±0.44 | 92.1±14.1 | |  |  |
|  |  |  | PBO+MET | 91 | 60.8±6.9 | 56 | 31.7±3.9 | 5.5±5.3 | 7.16±0.53 | 90.9±13.7 | |  |  |
| Barnett-1, 2014(368) | | 52 weeks | Empagliflozin 25mg (CKD2) | 97 | 62.0±8.4 | 62.9 | 31.3±5.8 | / | 7.96±0.73 | 86.4±0.45 | |  |  |
|  | |  | PBO (CKD2) | 95 | 62·6±8.1 | 58.9 | 30.8±5.6 | / | 8.09±0.80 | 88.4±0.45 | |  |  |
| Barnett-2, 2014(368) | | 52 weeks | Empagliflozin 25mg (CKD3) | 187 | 64.6±8.9 | 57.2 | 30.2±5.3 | / | 8.02±0.84 | 81.68±0.5 | |  |  |
|  | |  | PBO (CKD3) | 187 | 65.1±8.2 | 56.7 | 30.3±5.3 | / | 8.09±0.80 | 82.85±0.35 | |  |  |
| Barnett-3, 2014(368) | | 52 weeks | Empagliflozin 25mg (CKD4) | 37 | 65.4±10.2 | 56.8 | 29.0±4.9 | / | 8.06±1.05 | 76.5±15.9 | |  |  |
|  | |  | PBO (CKD4) | 37 | 62.9±11.9 | 51.4 | 31.8±6.0 | / | 8.16±0.99 | 84.1±21 | |  |  |
| Rosenstock, 2014(369) | | 52 weeks | Empagliflozin 25mg+INS | 189 | 58.0±9.4 | 44 | 35.0±4.0 | / | 8.29±0.72 | 93.41±1.72 | |  |  |
|  | |  | PBO+INS | 188 | 55.3±10.1 | 40 | 34.7±4.3 | / | 8.33±0.72 | 96.66±1.72 | |  |  |
| Cefalu, 2015(370) | | 52 weeks | Dapagliflozin 10mg+OAD/INS | 455 | 62.8±7.0 | 67.9 | 32.6±5.9 | 12.6±8.7 | 8.18±0.84 | 92.6±20.5 | |  |  |
|  |  |  | PBO+OAD/INS | 459 | 63.0±7.7 | 68.6 | 32.9±6.1 | 12.3±8.2 | 8.08±0.80 | 93.6±19.5 | |  |  |
| Neal, 2015 (338) | | 52 weeks | Canagliflozin300mg+INS | 664 | 63 | 65 | 33.3±6.2 | 16.3±7.4 | 8.3±0.9 | 94.8±21.3 | |  |  |
|  |  |  | PBO+INS | 639 | 63 | 66 | 33.1±6.5 | 16.0±7.8 | 8.3±0.9 | 94.8±22.3 | |  |  |
| Araki, 2017(371) | | 52 weeks | Dapagliflozin5/10mg qd+INS | 122 | 58.3±9.8 | 73.0 | / | 15.3±9.0 | 8.3±0.8 | 73.9±15.7 | |  |  |
|  | |  | PBO+INS | 60 | 57.6±9.9 | 66.7 | / | 14.2±8.9 | 8.5±0.9 | 71.9±13.4 | |  |  |
| Dagogo-Jack, 2018(372) | | 52 weeks | Ertugliflozin 5mg qd+MET1.5g qd +Sitagliptin 100mg qd | 156 | 59.2±9.3 | 51.9 | 31.2±5.5 | 9.9±6.1 | 8.1±0.9 | 87.6±18.6 | |  |  |
|  | |  | Ertugliflozin 15mg qd+MET 1.5g qd  +Sitagliptin100mg qd | 153 | 59.7±9.3 | 53.6 | 30.9±6.1 | 9.2±5.3 | 8.0±0.8 | 86.6±19.5 | |  |  |
|  | |  | PBO+MET1.5g qd+Sitagliptin 100mg qd | 153 | 58.3±9.2 | 65.4 | 30.3±6.4 | 9.4±5.6 | 8.0±0.9 | 86.4±20.8 | |  |  |
| Yale, 2017(373) | | 52 weeks | Canagliflozin 100mg qd+SU | 74 | 64.3±8.49 | 50.0 | / | 9.7±6.55 | 8.3±0.97 | 80.7±16.63 | |  |  |
|  | |  | Canagliflozin 300mgqd+SU | 72 | 65.8±7.88 | 58.3 | / | 8.8±6.24 | 8.1±0.95 | 80.5±18.97 | |  |  |
|  | |  | PBO+SU | 69 | 64.3±7.76 | 59.4 | / | 11.4±7.15 | 8.4±1.14 | 80.6±17.76 | |  |  |
| Hattori, 2018(374) | | 52 weeks | Empagliflozin10mg+OAD | 51 | 57.4±12.3 | 74.5 | 31 | / | 7.01±1.1 |  | |  |  |
|  | |  | PBO+OAD | 51 | 58.1±9.71 | 80.3 | 30 | / | 6.84±0.85 |  | |  |  |
| Jabbour, 2018(375) | | 52 weeks | Dapagliflozin 10mg qd+exenatide | 228 | 53.8±9.82 | 44.7 | / | / | / | / | |  |  |
|  | |  | PBO+exenatide | 227 | 54.2±9.62 | 51.1 | / | / | / | / | |  |  |
| Kawamori, 2018(376) | | 52 weeks | Empagliflozin 10/25mg+OAD | 182 | 60.0±9.9 | 78.0 | 26.0±3.8 | 9.0±7.2 | 8.27±0.65 | 71.2±12.6 | |  |  |
|  | |  | PBO+OAD | 93 | 59.8±10.8 | 77.4 | 26.6±4.5 | 8.7±6.1 | 8.36±0.74 | 73.1±15.9 | |  |  |
| Haering, 2015(377) | | 76 weeks | Empagliflozin 25mg  +MET+SU | 216 | 57.4±9.3 | 52.8 | 28.3±5.5 | / | 8.1±0.8 | 77.5±18.8 | |  |  |
|  | |  | PBO+MET+SU | 225 | 56.9±9.2 | 49.8 | 27.9±4.9 | / | 8.2±0.8 | 76.2±16.9 | |  |  |
| Merker, 2015(378) | | 76 weeks | Empagliflozin 25 mg+MET | 213 | 55.6±10.2 | 56.3 | 29.7±5.7 | / | 7.9±0.9 | 82.2±19.3 | |  |  |
|  |  |  | PBO+MET | 207 | 56.0±9.7 | 56 | 28.7±5.2 | / | 7.9±0.9 | 79.7±18.6 | |  |  |
| Bode, 2015(379) | | 104 weeks | Canagliflozin 300mg | 237 | 63.2±6.2 | 60.3 | 31.8±4.7 | 11.4±7.3 | 7.8±0.8 | 91.1±17.5 | |  |  |
|  | |  | PBO | 241 | 64.3±6.5 | 51.5 | 31.4±4.4 | 12.3±7.8 | 7.8±0.8 | 88.4±15.6 | |  |  |
| Perkovic, 2019(380) | | 168 weeks | Canagliflozin+INS | 2202 | 62.9±9.2 | 65.4 | 31.4±6.2 | 15.5±8.7 | 8.3±1.3 | / | |  |  |
|  | |  | PBO+INS | 2199 | 63.2±9.2 | 66.7 | 31.3±6.2 | 16.0±8.6 | 8.3±1.3 | / | |  |  |
| Neal, 2017(381) | | 188 weeks | Canagliflozin 300mg+INS | 1445 | 62.2±8 | 66.5 | / | / | / | / | |  |  |
|  | |  | Canagliflozin 100mg+INS | 1443 | 62.8±8.13 | 65.4 | / | / | / | / | |  |  |
|  | |  | PBO+INS | 1442 | 62.3±7.94 | 66.3 | / | / | / | / | |  |  |
| Neal, 2017(381) | | 188 weeks | Canagliflozin | 2907 | 63.9±8.42 | 63.8 | / | / | / | / | |  |  |
|  | |  | PBO | 2905 | 64±8.28 | 61.8 | / | / | / | / | |  |  |
| Wiviott, 2019(382) | | 4.2 years | Dapagliflozin 10mg qd+OAD | 8582 | 63.9±6.8 | 63.1 | 32.1±6.0 | 11.5±5 | 8.3±1.2 | / | |  |  |
|  | |  | PBO+OAD | 8578 | 64.0±6.8 | 62.1 | 32.0±6.1 | 10.0±5 | 8.3±1.2 | / | |  |  |
| **Exclusive PBO effect by injection** | | | | | | | | | | | |  |  |
| **GLP-1RA vs PBO** | |  |  |  |  |  |  |  |  |  | |  |  |
| Madsbad, 2004(59) | | 12 weeks | Liraglutide 0.045mg | 26 | 53±9.0 | 84.6 | 30.2±5.4 | 4.1±3.7 | 7.4±0.8 | / | |  |  |
|  | |  | Liraglutide 0.225mg | 24 | 58±7.5 | 62.5 | 32.0±5.3 | 4.4±4.0 | 7.9±0.8 | / | |  |  |
|  | |  | Liraglutide 0.45mg | 27 | 57±11.3 | 66.7 | 30.1±5.0 | 4.5±4.6 | 7.7±1.0 | / | |  |  |
|  | |  | Liraglutide 0.60mg | 30 | 57±7.7 | 66.7 | 30.4±4.8 | 4.6±4.6 | 7.4±1.2 | / | |  |  |
|  | |  | Liraglutide 0.75mg | 28 | 58±9.7 | 57.1 | 31.9±4.3 | 6.1±7.9 | 7.4±0.9 | / | |  |  |
|  | |  | PBO | 29 | 57±9.4 | 69 | 30.3±4.2 | 3.8±3.4 | 7.8±0.9 | / | |  |  |
|  | |  | Glimepiride | 26 | 57±9.2 | 61.5 | 30.2±4.6 | 3.4±2.9 | 7.4±1.2 | / | |  |  |
| Vilsbøll, 2007(383) | | 14 weeks | Liraglutide 0.65mg | 40 | 56.5±9.3 | 67.5 | 28.9±3.9 | 6 | 8.1±0.6 | / | |  |  |
|  | |  | Liraglutide 1.25mg | 42 | 53.8±10.7 | 54.8 | 31.2±4.7 | 7 | 8.3±0.8 | / | |  |  |
|  | |  | Liraglutide 1.9mg | 41 | 55.4±11.4 | 73.2 | 29.9±4.2 | 4 | 8.5±0.9 | / | |  |  |
|  | |  | PBO | 40 | 57.7±8.2 | 47.5 | 30.4±4.0 | 5 | 8.2±0.7 | / | |  |  |
| Seino, 2008(384) | | 14 weeks | Liraglutide 0.1mg | 45 | 56.5±8.4 | 68.9 | 24.26±2.77 | 7.15±5.14 | 8.50±0.84 | 64.82±10.29 | |  |  |
|  | |  | Liraglutide 0.3mg | 46 | 56.8±8.8 | 69.6 | 23.93±3.09 | 6.78±4.69 | 8.24±0.92 | 62.42±11.18 | |  |  |
|  | |  | Liraglutide 0.6mg | 45 | 60.0±7.0 | 62.2 | 23.74±2.78 | 8.87±6.77 | 8.21±0.83 | 61.97±9.40 | |  |  |
|  | |  | Liraglutide 0.9mg | 44 | 55.5±7.6 | 70.5 | 23.59±3.04 | 7.62±4.92 | 8.12±0.98 | 62.36±10.65 | |  |  |
|  | |  | PBO | 46 | 57.5±8.7 | 63 | 23.77±2.63 | 7.48±5.65 | 8.43±1.02 | 62.78±10.88 | |  |  |
| Vilsbøll, 2008(385) | | 14 weeks | Liraglutide 0.65mg | 8 | 61.1±7.6 | 100 | 26.6±2.8 | 7.1±2.5 | 8.7±0.7 | 83.1±13.1 | |  |  |
|  | |  | Liraglutide 1.25mg | 10 | 56.9±10.1 | 100 | 29.1±3.2 | 7.9±2.7 | 8.4±0.6 | 94.3±10.4 | |  |  |
|  | |  | Liraglutide 1.9mg | 11 | 58.6±10.3 | 86 | 31.4±3.3 | 4.6±3.2 | 8.2±0.6 | 93.1±19.7 | |  |  |
|  | |  | PBO | 10 | 55.4±6.7 | 80 | 30.3±4.3 | 1.8±0.8 | 8.1±0.3 | 84.5±19.5 | |  |  |
| Miyagawa, 2015(386) | | 26 weeks | Dulaglutide 0.75 mg qw | 280 | 57.2±9.6 | 81 | 25.6±3.6 | 6.8±5.6 | 8.15±0.77 | 71.3±12.5 | |  |  |
|  | |  | Liraglutide 0.9 mg qd | 137 | 57.9±10.4 | 83 | 25.5±3.5 | 6.3±6.0 | 8.08±0.89 | 70.2±12.5 | |  |  |
|  | |  | PBO | 70 | 57.7±8.3 | 79 | 25.2±3.2 | 6.3±5.1 | 8.20±0.83 | 69.3±11.6 | |  |  |
| Moretto, 2008(387) | | 24 weeks | Exenatide 5ug qd | 77 | 54±10 | 52 | 32±5 | 2±3 | 7.9±1.0 | 85±15 | |  |  |
|  | |  | Exenatide 10ug qd | 78 | 55±10 | 62 | 31±5 | 2±3 | 7.8±1.0 | 86±16 | |  |  |
|  | |  | PBO | 77 | 53±9 | 55 | 32±5 | 1±2 | 7.8±0.9 | 86±16 | |  |  |
| Rosenstock, 2009(388) | | 16 weeks | Exenatide 5-10ug bid | 34 | 53.7±9.4 | 45.7 | 32.4±5.1 | 6.4±5.4 | 8.0±0.9 | 94.2±23.2 | |  |  |
|  | |  | Albiglutide 4mg qw | 34 | 50.4±10.3 | 42.9 | 34.2±5.2 | 4.4±4.1 | 8.1±1.0 | 97.6±23.7 | |  |  |
|  | |  | Albiglutide 15mg qw | 34 | 55.5±10.5 | 51.4 | 31.1±4.1 | 4.7±4.6 | 8.0±0.9 | 88.4±14.9 | |  |  |
|  | |  | Albiglutide 30mg qw | 29 | 54.2±9.7 | 25.8 | 33.0±3.9 | 5.2±5.4 | 8.0±0.9 | 88.1±13.9 | |  |  |
|  | |  | Albiglutide 15mg q2w | 30 | 52.5±9.6 | 42.4 | 32.1±4.3 | 4.3±4.3 | 8.2±1.0 | 88.9±19.4 | |  |  |
|  | |  | Albiglutide 30mg q2w | 32 | 55.5±9.9 | 50 | 31.2±4.1 | 5.5±4.5 | 8.0±1.0 | 88.0±14.1 | |  |  |
|  | |  | Albiglutide 50mg q2w | 34 | 51.1±10.3 | 54.3 | 32.1±4.3 | 5.2±5.5 | 8.0±0.7 | 92.3±15.5 | |  |  |
|  | |  | Albiglutide 50mg monthly | 35 | 54.1±11.3 | 48.6 | 31.6±4.9 | 5.3±3.7 | 7.9±0.8 | 91.3±15.3 | |  |  |
|  | |  | Albiglutide 100mg monthly | 33 | 54.4±9.9 | 55.9 | 31.8±5.2 | 4.3±3.7 | 8.0±1.0 | 92.2±21.1 | |  |  |
|  | |  | PBO | 50 | 54.0±10.6 | 54.9 | 31.8±5.4 | 3.9±3.0 | 7.9±0.9 | 91.1±18.8 | |  |  |
| Seino,2014(389) | | 16 weeks | Albiglutide15mg qw | 52 | 53.3±10.3 | 61.5 | 25.3±3.5 | 6.3±4.6 | 8.54±0.52 | 68.6±12.7 | |  |  |
|  | |  | Albiglutide30mg qw | 54 | 58±9.3 | 70.4 | 25±3.8 | 7.8±5.4 | 8.54±0.86 | 67.4±12 | |  |  |
|  | |  | Albiglutide30mg biw | 53 | 59.1±8.5 | 77.4 | 25.3±3.8 | 7.2±5.6 | 8.54±0.81 | 68.2±13 | |  |  |
|  | |  | PBO | 53 | 57.5±11.1 | 69.8 | 25±3 | 6.7±7.8 | 8.57±0.79 | 66.3±11.2 | |  |  |
| Nauck, 2016(390) | | 52 weeks | Albiglutide 50mg qw | 99 | 52.0±11.8 | 50.5 | 33.9±5.5 | 4.2±4.6 | 8.2±0.9 | 97.1±17.8 | |  |  |
|  | |  | Albiglutide 30mg qw | 101 | 53.6±10.9 | 57.4 | 33.7±5.1 | 3.4±3.7 | 8.0±0.8 | 95.8±19.6 | |  |  |
|  | |  | PBO | 101 | 53.1±11.7 | 57.4 | 33.0±5.4 | 4.3±4.0 | 8.0±0.9 | 95.4±19.9 | |  |  |
| Grunberger, 2012(391) | | 12 weeks | Dulaglutide 0.1mg qw | 35 | 56.3±9.2 | 31.4 | 32.9±4.8 | 3.9±3.2 | 7.1±0.6 | 87.1±17.3 | |  |  |
|  | |  | Dulaglutide 0.5mg qw | 34 | 56.9±9.1 | 47.1 | 32.3±5.4 | 3.7±3.8 | 7.2±0.6 | 90.2±21.3 | |  |  |
|  | |  | Dulaglutide 1mg qw | 34 | 57.2±8.8 | 47.1 | 32.2±4.5 | 3.3±2.5 | 7.3±0.7 | 86.9±17.0 | |  |  |
|  | |  | Dulaglutide 1.5mg qw | 29 | 57.5±7.9 | 44.8 | 31.0±4.3 | 4.6±4.1 | 7.3±0.4 | 85.8±18.6 | |  |  |
|  | |  | PBO | 32 | 55.0±9.3 | 56.3 | 32.1±5.2 | 3.9±4.7 | 7.4±0.6 | 90.9±18.9 | |  |  |
| Terauchi, 2014(392) | | 12 weeks | Dulaglutide 0.75mg qd | 35 | 52.2±7.8 | 80 | 27.1±3.7 | 4.6±4.5 | 8.0±0.6 | 75.8±10.8 | |  |  |
|  | |  | Dulaglutide 0.5mg qd | 37 | 52.5±9.2 | 62.2 | 26.7±3.8 | 4.9±4.0 | 8.0±0.7 | 72.1±12.8 | |  |  |
|  | |  | Dulaglutide 0.25mg qd | 36 | 52.3±8.8 | 75 | 26.8±4.5 | 4.3±3.5 | 8.1±0.7 | 74.0±14.5 | |  |  |
|  | |  | PBO | 37 | 51.7±9.7 | 78.4 | 27.4±4.5 | 4.7±4.5 | 8.0±0.6 | 76.4±15.9 | |  |  |
| Fonseca, 2012(393) | | 12 weeks | Lixisenatide 2-step dose increase | 120 | 53.5±9.7 | 52.5 | 32.3±6.7 | 1.4 | 7.98±0.9 | 89±22 | |  |  |
|  | |  | Lixisenatide 1-step dose increase | 119 | 53.8±10.9 | 52.9 | 31.7±6.6 | 1.1 | 8.07±0.9 | 86.5±21 | |  |  |
|  | |  | PBO | 122 | 54.1±11.0 | 49.2 | 31.8±6.7 | 1.4 | 8.07±0.9 | 86.1±22 | |  |  |
| Raz, 2012(394) | | 24 weeks | Taspoglutide 20mg qw | 127 | 55.0±10.4 | 36 | 31.7±4.9 | 2.1±2.4 | 7.7±1.0 | 85.0±17.0 | |  |  |
|  | |  | Taspoglutide 10mg qw | 112 | 53.4±9.6 | 37 | 33.2±5.0 | 2.8±2.9 | 7.5±1.0 | 88.4±17.8 | |  |  |
|  | |  | PBO | 115 | 55.8±8.5 | 37 | 32.1±5.3 | 2.3±1.9 | 7.6±1.0 | 87.4±19.3 | |  |  |
| Hollander, 2013(395) | | 24 weeks | Taspoglutide 20mg qw | 149 | 53±10 | 42 | 36.9±5.0 | 5.2±4.3 | 7.54±0.84 | 103.6±19.5 | |  |  |
|  | |  | PBO | 143 | 54±10 | 39 | 36.5±4.8 | 4.9±4.1 | 7.55±0.84 | 101.4±16.4 | |  |  |
| **Augmented PBO effect by injection** | | | | | | | | | | | |  |  |
| **GLP-1RA vs PBO** | |  |  |  |  |  |  |  |  |  | |  |  |
| Ambery, 2018(396) | | 6 weeks | MEDI0382 | 25 | 56.0±7.2 | 52 | 32.0±4.0 | / | 7.2±0.6 | 95.9±18.9 | |  |  |
|  | |  | PBO | 26 | 56.9±6.2 | 58 | 33.4±3.4 | / | 7.3±0.7 | 99.9±15.1 | |  |  |
| Idorn, 2016(397) | | 12 weeks | Liraglutide+ESRD | 10 | 68.3±3.1 | 80 | 31.6±2.4 | 15.3±2.3 | 6.7±0.4 | / | |  |  |
|  | |  | PBO+ESRD | 10 | 65.9±4.4 | 90 | 31.5±2.4 | 13.0±2.4 | 6.6±0.4 | / | |  |  |
|  | |  | Control+Liraglutide | 10 | 60.7±3.2 | 70 | 30.2±1.3 | 9.9±3.0 | 7.9±0.4 | / | |  |  |
|  | |  | Control+PBO | 10 | 63.1±2.1 | 80 | 30.8±1.0 | 16.0±2.7 | 7.8±0.4 | / | |  |  |
| Tonneijck, 2016(398) | | 12 weeks | Liraglutide 1.8mg qd | 19 | 60.5±7.2 | 74 | 32.0±3.9 | 7±6 | 7.4±0.7 | 106.0±17.2 | |  |  |
|  | |  | Sitagliptin 100mg qd | 19 | 61.7±6.8 | 84 | 30.4±5.4 | 6±6 | 7.1±0.5 | 99.4±17.6 | |  |  |
|  | |  | PBO | 17 | 65.8±5.8 | 77 | 30.8±1.9 | 8±4 | 7.5±0.7 | 95.8±9.8 | |  |  |
| Scholten,2017 (399) | | 12 weeks | Liraglutide 1.8mg qd+OAD | / | / | / | / | / | 7.9±3.24 | 99.7±18.7 | |  |  |
|  | |  | PBO+OAD | / | / | / | / | / | 7.64±3.24 | 99.5±17.8 | |  |  |
| Gao, 2009(400) | | 12 weeks | Exenatide 10ug bid+MET or MET/SU | 234 | 55±9 | 48 | 26.4±3.2 | 8±6 | 8.3±1.0 | 69.6±11.2 | |  |  |
|  | |  | PBO+MET or MET/SU | 232 | 54±9 | 41 | 26.1±3.4 | 8±5 | 8.3±1.0 | 67.9±11.1 | |  |  |
| Kadowaki, 2009(401) | | 12 weeks | Exenatide 2.5ug bid | 37 | 62.2±7.8 | 70.3 | 24.2±3.3 | 14.8±10.9 | 8.0±0.8 | 64.9±11.6 | |  |  |
|  | |  | Exenatide 5ug bid | 37 | 60.7±9.8 | 67.6 | 25.0±3.4 | 11.3±6.4 | 7.9±0.8 | 65.6±9.8 | |  |  |
|  | |  | Exenatide 10ug bid | 37 | 57.8±10.4 | 62.6 | 26.1±5.3 | 9.6±6.0 | 7.9±0.9 | 70.3±15.9 | |  |  |
|  | |  | PBO | 40 | 60.5±10.2 | 75 | 25.8±4.6 | 11.9±6.0 | 8.1±0.7 | 71.1±14 | |  |  |
| Gill, 2010(402) | | 12 weeks | Exenatide 10μg bid | 28 | 57±11 | 68 | 29.5±3.4 | 7±4 | 7.5±0.9 | 91.6±15.2 | |  |  |
|  | |  | PBO | 26 | 54±10 | 42 | 30.1±3.9 | 6±4 | 7.1±0.7 | 85.9±12.2 | |  |  |
| Nauck, 2016(403) | | 12 weeks | Semaglutide 0.1mg qw | 47 | 55.2±10.1 | 66 | 31.5±4.6 | 3.6±5.0 | 8.2±0.9 | 89.5±14.2 | |  |  |
|  | |  | Semaglutide 0.2mg qw | 43 | 54.7±10.0 | 70 | 30.4±3.9 | 2.3±2.7 | 8.2±0.9 | 86.3±15.1 | |  |  |
|  | |  | Semaglutide 0.4mg qw | 48 | 53.8±10.2 | 77 | 29.7±4.5 | 2.0±2.3 | 8.1±0.9 | 87.0±14.0 | |  |  |
|  | |  | Semaglutide 0.8mg qw | 42 | 55.0±9.7 | 52 | 30.7±4.5 | 3.0±3.0 | 8.2±0.9 | 85.9±15.1 | |  |  |
|  | |  | Semaglutide 0.8mg qw evening | 43 | 55.9±7.9 | 63 | 31.2±4.2 | 2.6±2.1 | 8.0±0.8 | 85.7±12.6 | |  |  |
|  | |  | Semaglutide 1.6mg qw evening | 47 | 56.4±10.5 | 55 | 30.9±4.7 | 1.8±2.0 | 8.0±0.7 | 84.5±14.0 | |  |  |
|  | |  | Liraglutide 1.2mg qd | 45 | 54.8±9.2 | 69 | 31.0±4.6 | 3.3±3.4 | 8.0±0.8 | 90.5±13.5 | |  |  |
|  | |  | Liraglutide 1.8mg qd | 50 | 54.3±10.1 | 70 | 30.9±4.6 | 2.5±2.6 | 8.1±0.7 | 87.2±13.1 | |  |  |
|  | |  | PBO | 46 | 55.3±10.6 | 61 | 31.7±3.8 | 2.4±3.3 | 8.1±0.8 | 90.5±13.0 | |  |  |
| Ratner, 2010(404) | | 13 weeks | Lixisenatide 5ug qd+MET | 55 | 56.8±7.8 | 47.3 | 30.7±4.6 | 7.2±4.9 | 7.58±0.7 | 84.6±16 | |  |  |
|  | |  | Lixisenatide 10ug qd+MET | 52 | 55.4±9.2 | 59.6 | 31.9±4.0 | 6.2±4.1 | 7.52±0.6 | 90.5±17 | |  |  |
|  | |  | Lixisenatide 20ug qd+MET | 55 | 55.4±9.9 | 50.9 | 32.0±4.3 | 6.4±6.8 | 7.58±0.7 | 89.4±17 | |  |  |
|  | |  | Lixisenatide 30ug qd+MET | 54 | 56.5±8.7 | 50 | 31.6±3.6 | 6.0±4.8 | 7.52±0.7 | 87.6±15 | |  |  |
|  | |  | Lixisenatide 5ug bid+MET | 53 | 57.1±8.2 | 47.2 | 31.6±4.2 | 6.2±6.0 | 7.60±0.6 | 86.5±14 | |  |  |
|  | |  | Lixisenatide 10ug bid+MET | 56 | 56.0±7.9 | 51.8 | 32.8±4.4 | 6.4±5.0 | 7.54±0.6 | 89.8±17 | |  |  |
|  | |  | Lixisenatide 20ug bid+MET | 54 | 56.7±8.3 | 37 | 32.7±4.4 | 6.6±5.1 | 7.61±0.7 | 88.5±17 | |  |  |
|  | |  | Lixisenatide 30ug bid+MET | 54 | 55.3±9.1 | 42.6 | 32.3±4.5 | 7.0±5.4 | 7.46±0.5 | 87.5±13 | |  |  |
|  | |  | PBO | 109 | 56.3±9.2 | 56 | 31.7±4.2 | 7.1±5.4 | 7.53±0.6 | 87.7±14 | |  |  |
| Kim, 2007(405) | | 15 weeks | Exenatide LAR 0.8mg qw+MET | 15 | 51±12 | 75 | 35±6 | 5±3 | 8.6±1.1 | 107±24 | |  |  |
|  | |  | Exenatide LAR 2.0mg qw+MET | 15 | 51±11 | 67 | 36±6 | 4±5 | 8.3±1.1 | 110±17 | |  |  |
|  | |  | PBO+MET | 15 | 55±9 | 36 | 36±6 | 4±4 | 8.6±1.4 | 101±20 | |  |  |
| Umpierrez,2011(406) | | 16 weeks | LY 2189265 0.5/1.0mg | 66 | 59±12 | 53 | 33.7±4.1 | 9.0±7.6 | 8.25±0.9 | 94.8±17 | |  |  |
|  | |  | LY 2189265 1.0/1.0mg | 65 | 57±12 | 54 | 33.9±4.0 | 8.1±5.4 | 8.25±1.0 | 96.7±17 | |  |  |
|  | |  | LY 2189265 1.0/2.0mg | 65 | 54±11 | 52 | 34.2±4.1 | 8.6±6.9 | 8.43±1.0 | 98.6±18.4 | |  |  |
|  | |  | PBO | 66 | 56±12 | 44 | 33.9±4.3 | 7.5±5.4 | 8.05±0.8 | 94.7±15 | |  |  |
| Kaku, 2010(407) | | 24 weeks | Liraglutide 0.6mg qd+SU | 88 | 59.1±10.3 | 60 | 25.3±3.6 | 9.3±5.8 | 8.60±0.91 | 66.1±12.1 | |  |  |
|  | |  | Liraglutide 0.9mg qd+SU | 88 | 61.3±11.0 | 67 | 24.4±3.4 | 11.6±7.7 | 8.21±0.78 | 64.5±12.0 | |  |  |
|  | |  | PBO+SU | 88 | 58.6±9.7 | 65 | 24.9±4.0 | 10.1±7.3 | 8.45±0.99 | 66.7±13.5 | |  |  |
| Lind, 2015(408) | | 24 weeks | Liraglutide 1.8mg qd | 64 | 63.7±8.2 | 62.5 | 33.7±4.3 | 17.3±7.6 | 9.0±1.0 | 98.9±14.0 | |  |  |
|  | |  | PBO | 60 | 63.5±7.7 | 66.7 | 33.5±4.0 | 17.0±8.1 | 9.0±1.1 | 100.0±14.8 | |  |  |
| Apovian, 2010(409) | | 24 weeks | Exenatide 10ug bid+MET | 52 | 53.4±10.7 | 29 | 32.9±3.5 | 4.3±3.2 | 7.5±0.8 | 91.4±13.2 | |  |  |
|  | |  | Exenatide10ug bid+ MET+SU | 33 | 55.9±9.3 | 48 | 34.0±3.7 | 8.5±7.5 | 8.0±0.8 | 98.6±20.1 | |  |  |
|  | |  | Exenatide 10ug bid+SU | 11 | 55.6±8.7 | 45 | 36.2±4.2 | 4.0±3.5 | 7.9±1.1 | 100.6±16.5 | |  |  |
|  | |  | PBO+MET | 51 | 55.0±7.9 | 39 | 33.6±4.6 | 3.9±3.2 | 7.2±0.5 | 94.9±14.3 | |  |  |
|  | |  | PBO+MET+SU | 36 | 55.1±9.9 | 31 | 34.3±4.0 | 7.6±6.9 | 7.9±0.9 | 96.5±17 | |  |  |
|  | |  | PBO+SU | 11 | 55.3±11.3 | 54 | 33.8±4.3 | 4.3±2.8 | 7.7±1.1 | 100.8±17.4 | |  |  |
| Ludvik, 2018(410) | | 24 weeks | Duraglutide1.5mg qd | 142 | 56.2±9.26 | 54 | 32.87±5.56 | 9.21±5.74 | 8.04±0.65 | 92.87±19.73 | |  |  |
|  | |  | PBO | 140 | 57.1±9.59 | 47 | 32.39±4.98 | 8.87±6.13 | 8.05±0.66 | 90.05±19.47 | |  |  |
| Dungan, 2016(411) | | 24 weeks | Dulaglutide 1.5mg qw+SU | 239 | 57.7±10.2 | 43.5 | 30.9±5.2 | 7.8±5.3 | 8.4±0.7 | 84.5±16.4 | |  |  |
|  | |  | PBO+SU | 60 | 58.2±7.4 | 46.7 | 32.4±5.9 | 6.8±3.8 | 8.4±0.7 | 89.5±18.6 | |  |  |
| Seino, 2012(412) | | 24 weeks | Lixisenatide 20μg qd+INS±SU | 154 | 58.7±10.2 | 44.8 | 25.4±3.7 | 13.7±7.7 | 8.54±0.73 | 65.93±13.00 | |  |  |
|  | |  | PBO+INS±SU | 157 | 58.0±10.1 | 51.0 | 25.2±3.9 | 14.1±7.7 | 8.52±0.78 | 65.60±12.47 | |  |  |
| Ahrén, 2013(413) | | 24 weeks | Lixisenatide morning+MET | 255 | 54.5±9.2 | 38.4 | 33.2±6.9 | 6.2±5.3 | 8.0±0.9 | 90.1±21.0 | |  |  |
|  | |  | Lixisenatide evening+MET | 255 | 54.8±10.4 | 44.7 | 32.5±5.8 | 6.2±5.4 | 8.1±0.9 | 89.0±20.7 | |  |  |
|  | |  | PBO+MET | 170 | 55.0±9.4 | 47.6 | 33.1±6.5 | 5.9±4.7 | 8.1±0.9 | 90.4±20.1 | |  |  |
| Riddle, 2013-1(414) | | 24 weeks | Lixisenatide+INS+MET | 223 | 56±10 | 49 | 32.0±6.6 | 9.6±6.0 | 7.6±0.5 | 87.3±21.8 | |  |  |
|  | |  | PBO+INS+MET | 223 | 56±10 | 51 | 31.7±6.0 | 8.7±5.8 | 7.6±0.5 | 86.8±20.4 | |  |  |
| Riddle, 2013-2(415) | | 24 weeks | Lixisenatide+INS±MET | 328 | 57±10 | 45 | 31.9±6.2 | 12.5±7.0 | 8.4±0.9 | 87±20 | |  |  |
|  | |  | PBO+INS±MET | 167 | 57±10 | 49 | 32.6±6.3 | 12.4±6.3 | 8.4±0.8 | 89±21 | |  |  |
| Bolli, 2014(416) | | 24 weeks | Lixisenatide one-step+MET | 161 | 55.4±8.9 | 44 | 33.0±5.8 | 3.3±2.6 | 8.0±0.9 | 90.3±19.0 | |  |  |
|  | |  | Lixisenatide two-step+MET | 161 | 54.6±8.9 | 45 | 32.1±4.8 | 3.7±3.4 | 8.1±0.9 | 88.0±16.8 | |  |  |
|  | |  | PBO+MET | 160 | 58.2±9.8 | 45 | 32.4±5.5 | 3.6±3.1 | 8.0±0.8 | 87.9±17.3 | |  |  |
| Pan, 2014(417) | | 24 weeks | Lixisenatide 20μg qd | 196 | 54.5±10.3 | 51.5 | 26.8±3.9 | 6.5±4.6 | 7.95±0.81 | 73.18±13.93 | |  |  |
|  | |  | PBO | 194 | 55.1±10.5 | 46.9 | 27.1±3.8 | 6.8±4.8 | 7.85±0.71 | 72.74±13.64 | |  |  |
| Rosenstock, 2014(418) | | 24 weeks | Lixisenatide+SU±MET | 574 | 57.0±9.8 | 49.5 | 30.1±6.6 | 9.1±6.0 | 8.3±0.9 | 82.6±21.9 | |  |  |
|  | |  | PBO+SU±MET | 285 | 57.8±10.1 | 52.6 | 30.4±6.6 | 9.8±6.2 | 8.2±0.8 | 84.5±22.8 | |  |  |
| Meneilly, 2017(419) | | 24 weeks | Lixisenatide 20μg qd | 176 | 74.0±4.0 | 52.3 | 29.9±3.7 | 13.6±7.3 | 8.1±0.7 | 80.8±14.5 | |  |  |
|  | |  | PBO | 174 | 74.4±3.8 | 51.7 | 30.1±4.5 | 14.6±7.9 | 8.1±0.7 | 80.1±16.8 | |  |  |
| Yang, 2017(420) | | 24 weeks | Lixisenatide 20ug qd+ INS±MET | 224 | 53.9±9.9 | 46.9 | 27.5±4.39 | 10.3±6.1 | 7.9±0.66 | 74.2±14.1 | |  |  |
|  | |  | PBO+INS±MET | 224 | 56.2±9.1 | 43.8 | 27.9±4.48 | 10.2±6.2 | 7.9±0.70 | 74.6±13.3 | |  |  |
| Bergenstal, 2012(302) | | 24 weeks | Taspoglutide 10mg | 182 | 55.3±9.5 | 56 | 32.7±5.2 | 6.1±4.8 | 7.95±0.93 | 93.6±20.4 | |  |  |
|  |  |  | Taspoglutide 20mg | 187 | 56.8±8.8 | 52 | 32.3±5 | 5.7±4.7 | 7.97±0.86 | 91.8±18.0 | |  |  |
|  |  |  | Sitagliptin 100mg | 177 | 55.5±9.9 | 59 | 32.4±5 | 6±5 | 7.94±0.85 | 92.5±19.7 | |  |  |
|  |  |  | PBO | 90 | 56.1±10.1 | 52 | 32.5±5.5 | 5.5±3.9 | 8.03±0.83 | 91.1±19.0 | |  |  |
| Henry, 2012(421) | | 24 weeks | Taspoglutide 10mg qw+MET+PIO | 106 | 52.5±10.3 | 59 | 32.8±5.3 | 7.3±4.6 | 8.2±1.0 | 94.0±22.3 | |  |  |
|  | |  | Taspoglutide 20mg qw+MET+PIO | 113 | 55.5±10.1 | 53 | 33.0±5.0 | 8.3±5.3 | 8.1±0.9 | 93.5±21.8 | |  |  |
|  | |  | PBO+MET+PIO | 94 | 54.3±9.6 | 50 | 32.0±5.3 | 7.5±5.8 | 8.1±0.9 | 88.5±20.6 | |  |  |
| Vanderheiden, 2016(422) | | 6 months | Liraglutide 1.8mg qd+INS | 35 | 52.8±8.1 | 34 | 40.7±6.7 | 16±7 | 9.0±1.2 | 114.6±21.4 | |  |  |
|  | |  | PBO+INS | 36 | 55.5±6.6 | 39 | 41.6±10.4 | 18±9 | 8.9±1.0 | 113.6±20.8 | |  |  |
| Frias, 2018(423) | | 26 weeks | Duraglutide1.5mg qw | 54 | 58.7±7.8 | 44 | 32.4±5.4 | 9.3±7.1 | 8.1±1.0 | 89.8±16.9 | |  |  |
|  | |  | LY329817615mg | 53 | 56.0±7.6 | 42 | 32.3±6.2 | 8.5±6.1 | 8.1±1.1 | 89.1±22.7 | |  |  |
|  | |  | PBO | 51 | 56.6±8.9 | 57 | 32.4±6.0 | 8.6±7.0 | 8.0±0.9 | 91.5±23.1 | |  |  |
| Marre, 2009(257) | | 26 weeks | Liraglutide 0.6mg qd+glimipiride 2-4mg qd | 233 | 55.7±9.9 | 54 | 30.0±5.0 | 6.5 | 8.4±1.0 | 82.6±17.7 | |  |  |
|  | |  | Liraglutide 1.2mg qd+glimipiride 2-4mg qd | 228 | 57.7±9.0 | 45 | 29.8±5.1 | 6.7 | 8.5±1.1 | 80.0±17.1 | |  |  |
|  | |  | Liraglutide 1.8mg qd+glimipiride 2-4mg qd | 234 | 55.6±10.0 | 53 | 30.0±5.1 | 6.5 | 8.5±0.9 | 83.0±18.1 | |  |  |
|  | |  | PBO+glimipiride 2-4mg qd | 114 | 54.7±10.0 | 47 | 30.3±5.4 | 6.5 | 8.4±1.0 | 81.9±17.1 | |  |  |
|  | |  | Rosiglitazone 4mg qd+glimipiride 2-4mg qd | 232 | 56.0±9.8 | 47 | 29.4±4.8 | 6.6 | 8.4±1.0 | 80.6±17.0 | |  |  |
| Nauck, 2009(206) | | 26 weeks | Liraglutide 0.6mg qd+MET | 242 | 56±11 | 62 | 30.5±4.8 | 7±5 | 8.4±0.9 | / | |  |  |
|  | |  | Liraglutide 1.2mg qd+MET | 240 | 57±9 | 54 | 31.1±4.8 | 7±5 | 8.3±1.0 | / | |  |  |
|  | |  | Liraglutide 1.8mg qd+MET | 242 | 57±9 | 59 | 30.9±4.6 | 8±5 | 8.4±1.0 | / | |  |  |
|  | |  | Glimepiride 4mg qd+MET | 242 | 57±9 | 57 | 31.2±4.6 | 8±5 | 8.4±1.0 | / | |  |  |
|  | |  | PBO+MET | 121 | 56±9 | 60 | 31.6±4.4 | 8±6 | 8.4±1.1 | / | |  |  |
| Russell-Jones, 2009(424) | | 26 weeks | Liraglutide 1.8mg qd+MET+SU | 230 | 57.6±9.5 | 57 | 30.4±5.3 | 9.2±5.8 | 8.3±0.9 | 85.5±19.4 | |  |  |
|  | |  | PBO+MET+SU | 114 | 57.5±9.6 | 49 | 31.3±5.0 | 9.4±6.2 | 8.3±0.9 | 85.7±16.7 | |  |  |
| Zinman, 2009(425) | | 26 weeks | Liraglutide 1.2mg qd+MET 1g bid+RSG 4mg bid | 178 | 55±10 | 57 | 33.3±5.4 | 9±6 | 8.5±1.2 | / | |  |  |
|  | |  | Liraglutide 1.8mg qd+MET 1g bid+RSG 4mg bid | 178 | 55±11 | 51 | 33.5±5.1 | 9±6 | 8.6±1.2 | / | |  |  |
|  | |  | PBO+MET 1g bid+RSG 4mg bid | 177 | 55±10 | 62 | 33.9±5.2 | 9±6 | 8.4±1.2 | / | |  |  |
| Ahmann, 2015(426) | | 26 weeks | Liraglutide 1.8mg qd +INS±MET | 225 | 59.3±9.2 | 53.3 | 32.3±5.6 | 12.1±7.1 | 8.2±0.8 | 90.2±20.0 | |  |  |
|  | |  | PBO +INS±MET | 225 | 57.5±11.1 | 60.4 | 32.2±5.7 | 12.1±6.8 | 8.3±0.9 | 91.9±19.3 | |  |  |
| Davies, 2016(427) | | 26 weeks | Liraglutide 1.8mg qd | 140 | 68±8.3 | 53.6 | 33.4±5.4 | 15.9±8.9 | 8.08±0.792 | 93.63±17.41 | |  |  |
|  | |  | PBO | 137 | 66.3±8 | 47.4 | 34.5±5.4 | 14.2±7.5 | 8.0±0.853 | 95.63±17.65 | |  |  |
| Lingvay, 2018(428) | | 26 weeks | Liraglutide 1.8mg qd | 65 | 55.8±9.2 | 50.8 | 32.1±4.5 | 6.6±5.2 | 8.1±0.8 | 93.4±19.3 | |  |  |
|  | |  | Semaglutide 0.05-0.3mg qd | 64 | 54.8±9.7 | 56.2 | 33.2±4.4 | 8.0±7.1 | 8.1±0.9 | 95.3±15.4 | |  |  |
|  | |  | PBO | 129 | 57.1±9.2 | 55.8 | 32.8±4.2 | 7.1±4.5 | 8.1±0.9 | 94.0±17.8 | |  |  |
| Zinman, 2007(429) | | 26 weeks | Exenatide 10ug bid+TZD±MET | 121 | 55.6±10.8 | 53.7 | 34.0±5.1 | 7.3±4.9 | 7.9±0.9 | 97.5±18.8 | |  |  |
|  | |  | PBO+TZD±MET | 112 | 53.7±10.2 | 57.1 | 34.0±5.0 | 8.2±5.8 | 7.9±0.8 | 96.9±19.0 | |  |  |
| Liutkus, 2010(430) | | 26 weeks | Exenatide 10μg bid | 111 | 55±8 | 60 | 34±6 | 6.3±4.2 | 8.2±0.9 | 94.5±17.8 | |  |  |
|  | |  | PBO | 54 | 54±9 | 57 | 33±5 | 6.4±4.6 | 8.3±0.9 | 92.6±18.0 | |  |  |
| Wysham, 2014(431) | | 26 weeks | Dulaglutide 1.5mg qw+MET+PIO | 279 | 56±10 | 58 | 33±5 | 9±6 | 8.1±1.3 | 96±20 | |  |  |
|  | |  | Dulaglutide 0.75mg qw+MET+PIO | 280 | 56±9 | 60 | 33±6 | 9±5 | 8.1±1.2 | 96±21 | |  |  |
|  | |  | Exenatide+MET+PIO | 276 | 55±10 | 57 | 34±5 | 9±6 | 8.1±1.3 | 97±19 | |  |  |
|  | |  | PBO+MET+PIO | 141 | 55±10 | 59 | 33±6 | 9±6 | 8.1±1.3 | 94±19 | |  |  |
| Guja, 2018(432) | | 28 weeks | Exenatide 2mg qd | 231 | 57.8±9.0 | 49.4 | 33.3±6.1 | 11.5±6.6 | 8.53±0.91 | 93.3±20.0 | |  |  |
|  | |  | PBO | 230 | 57.6±10.3 | 46.5 | 34.1±6.6 | 11.1±6.1 | 8.53±0.92 | 94.7±19.8 | |  |  |
| Pozzilli, 2017(433) | | 28 weeks | Dulaglutide 1.5mg qw+INS | 150 | 60.2±9.5 | 56.7 | 32.8±4.9 | 13.0±7.5 | 8.4±0.9 | 93.3±17.5 | |  |  |
|  | |  | PBO+INS | 150 | 60.6±10.1 | 58.7 | 32.6±4.9 | 13.3±7.7 | 8.3±0.8 | 92.6±17.1 | |  |  |
| Buse, 2011(434) | | 30 weeks | Exenatide 10μg bid | 138 | 58.7±8.91 | 70 | 33.8±5.8 | 12±7 | 8.32±0.85 | 95.4±20.4 | |  |  |
|  | |  | PBO | 123 | 59.4±9.96 | 78 | 33.1±6.2 | 12±7 | 8.50±0.96 | 93.4±21.2 | |  |  |
| Buse, 2014(435) | | 30 weeks | Exenatide10ug bid+INS | 39 | 54±10 | 54 | 36±6 | 10±5 | 8.7±0.9 | 102±22 | |  |  |
|  | |  | PBO+INS | 49 | 56±8 | 61 | 36±5.9 | 11±6 | 8.8±1 | 101±21 | |  |  |
| Buse, 2004(436) | | 30 weeks | Exenatide 5ug bid+SU | 125 | 55±10 | 59.2 | 33±6 | 6.3±5.2 | 8.5±1.1 | 95±22 | |  |  |
|  | |  | Exenatide 10ug bid+SU | 129 | 56±11 | 57.4 | 33±6 | 6.6±6.6 | 8.6±1.2 | 95±18 | |  |  |
|  | |  | PBO+SU | 123 | 55±11 | 62.6 | 34±5 | 5.7±4.7 | 8.7±1.2 | 99±18 | |  |  |
| Defronzo, 2005(437) | | 30 weeks | Exenatide 5ug bid+MET | 110 | 55±11 | 51.8 | 34±6 | 6.2±5.9 | 8.3±1.1 | 100±22 | |  |  |
|  | |  | Exenatide 10ug bid+MET | 113 | 52±11 | 60.2 | 34±6 | 4.9±4.7 | 8.2±1.0 | 101±20 | |  |  |
|  | |  | PBO+MET | 113 | 54±9 | 59.3 | 34±6 | 6.6±6.1 | 8.2±1.0 | 100±19 | |  |  |
| Kendall, 2005(438) | | 30 weeks | Exenatide 5ug bid+MET+SU | 245 | 55±9 | 59.2 | 33±6 | 8.7±5.9 | 8.5±1.0 | 97±19 | |  |  |
|  | |  | Exenatide 10ug bid+MET+SU | 241 | 55±10 | 59.3 | 34±6 | 8.7±6.4 | 8.5±1.1 | 98±21 | |  |  |
|  | |  | PBO+MET+SU | 247 | 56±10 | 55.9 | 34±5 | 9.4±6.2 | 8.5±1.0 | 99±19 | |  |  |
| Rodbard, 2018(439) | | 30 weeks | Semaglutide1.0mg qw | 131 | 558.5±25 | 58.8 | 32.0±16.1 | / | 8.3±1.95 | 92.5±58.5 | |  |  |
|  | |  | PBO | 133 | 58.8±33.5 | 53.4 | 31.8±13.9 | / | 8.4±2.15 | 89.9±4.9 | |  |  |
| Seino, 2016(440) | | 36 weeks | Liraglutide 0.9mg qd+INS | 127 | 61.3±11.0 | 54.3 | 26.2±4.9 | 14.32±8.89 | 8.8±0.9 | 67.7±15.2 | |  |  |
|  | |  | PBO+INS | 130 | 59.8±11.3 | 57.7 | 25.2±4.0 | 14.69±8.60 | 8.8±0.9 | 65.9±13.0 | |  |  |
| Reusch, 2014(441) | | 52 weeks | Albiglutide 30mg qw+PIO±MET | 150 | 55.2±9.98 | 61.3 | 33.6±5.9 | 8.0±5.6 | 8.1±1.0 | 97.6±22.0 | |  |  |
|  | |  | PBO+PIO±MET | 151 | 54.9±9.40 | 58.3 | 34.7±5.6 | 7.9±6.1 | 8.1±0.9 | 100.2±23.1 | |  |  |
| Weinstock,2014(442) | | 52 weeks | Dulaglutide 1.5mg qw | 304 | 54±10 | 48 | 31±5 | 7±6 | 8.1±1.1 | 87±17 | |  |  |
|  | |  | Dulaglutide 0.75mg qw | 302 | 54±10 | 44 | 31±4 | 7±5 | 8.2±1.1 | 86±18 | |  |  |
|  | |  | Stagliptin 100mg qd | 315 | 54±10 | 48 | 31±4 | 7±5 | 8.1±1.1 | 86±17 | |  |  |
|  | |  | PBO | 177 | 55±9 | 51 | 31±4 | 7±5 | 8.1±1.1 | 87±17 | |  |  |
| Davies, 2015(443) | | 56 weeks | Liraglutide 3.0mg qd | 423 | 55.0±10.8 | 52.0 | 37.1±6.5 | 7.5±5.65 | 7.9±0.8 | 105.7±21.9 | |  |  |
|  | |  | Liraglutide 1.8mg qd | 211 | 54.9±10.7 | 51.2 | 37.0±6.9 | 7.4±5.16 | 8.0±0.8 | 105.8±21.0 | |  |  |
|  | |  | PBO | 212 | 54.7±9.8 | 45.8 | 37.4±7.1 | 6.7±5.07 | 7.9±0.8 | 106.5±21.3 | |  |  |
| Pinget, 2013(444) | | 76 weeks | Lixisenatide 20μg qd+PIO±MET | 323 | 56.0±9.5 | 53 | 33.7±6.7 | 8.1±5.4 | 8.1±0.9 | 92.9±22.9 | |  |  |
|  | |  | PBO+PIO±MET | 161 | 55.3±9.5 | 51 | 34.4±7.0 | 8.1±5.6 | 8.1±0.8 | 96.7±25.6 | |  |  |
| Hernandez, 2018(445) | | 1.6 years | Albiglutide 30-50mg qw | 4731 | 64.1±8.7 | 70 | 32.3±5.9 | 14.1±8.6 | 8.76±1.5 | 93±2.5 | |  |  |
|  | |  | PBO | 4732 | 64.2±8.7 | 69 | 32.3±5.9 | 14.2±8.9 | 8.72±1.5 | 93±2.5 | |  |  |
| Ahrén, 2014(446) | | 104 weeks | Albiglutide 30mg qw+MET | 302 | 54.3±10.1 | 44.7 | 32.7±5.6 | 6.0±4.3 | 8.1±0.8 | 89.6±18.4 | |  |  |
|  | |  | Sitagliptin 100mg qd+MET | 302 | 54.3±9.8 | 46.0 | 32.5±5.4 | 5.8±4.8 | 8.1±0.8 | 90.3±19.1 | |  |  |
|  | |  | Glimeripiride 2mg qd+MET | 307 | 54.4±10.0 | 51.5 | 32.5±5.5 | 6.0±4.8 | 8.1±0.8 | 91.8±20.4 | |  |  |
|  | |  | PBO+MET | 101 | 56.1±10.0 | 49.5 | 32.8±5.4 | 6.7±6.6 | 8.2±0.9 | 91.6±19.3 | |  |  |
| Marso, 2016(447) | | 104 weeks | Semaglutide 0.5mg qd | 826 | 64.6±7.3 | 59.9 | 32.7±6.29 | 14.3±8.2 | 8.7±1.4 | 91.8±20.3 | |  |  |
|  | |  | Semaglutide 1.0mg qd | 822 | 64.7±7.1 | 63.0 | 32.9±6.18 | 14.1±8.2 | 8.7±1.5 | 92.9±21.1 | |  |  |
|  | |  | PBO 0.5mg qd | 824 | 64.8±7.6 | 58.5 | 32.9±6.35 | 14.0±8.5 | 8.7±1.5 | 91.8±20.3 | |  |  |
|  | |  | PBO 1.0mg qd | 825 | 64.4±7.5 | 61.5 | 32.7±5.97 | 13.2±7.4 | 8.7±1.5 | 92.1±20.6 | |  |  |
| Home, 2015(448) | | 156 weeks | Albiglutide 30mg qw+MET+SU | 271 | 54.5±9.5 | 49.8 | 32.4±5.5 | 8.5±6.3 | 8.19±0.91 | 90.9±20.2 | |  |  |
|  | |  | PBO+MET+SU | 115 | 55.7±9.6 | 60.9 | 31.8±4.9 | 9.3±6.1 | 8.26±0.98 | 89.9±18.8 | |  |  |
| Holman, 2017 (449) | | 3.2 years | Exenatide 2mg qw+OAD+INS | 7356 | 62 | 62 | 31.8 | 12±5 | 8±0.8 |  | |  |  |
|  | |  | PBO+OAD+INS | 7396 | 62 | 62 | 31.7 | 12±5 | 8±0.8 |  | |  |  |
| Marso, 2016 (450) | | 182 weeks | Liraglutide 1.8mg qd | 4668 | 64.2±7.2 | 64.5 | 32.5± 6.3 | 12.8±8.0 | 8.7 ± 1.6 | 91.9±21.2 | |  |  |
|  | |  | PBO | 4672 | 64.4±7.2 | 64 | 32.5±6.3 | 12.9±8.1 | 8.7 ± 1.5 | 91.6±20.8 | |  |  |

**Table S3: Risk of bias of included RCT studies with type 1 diabetes**

|  | **Selection bias (random sequence generation)** | **Selection bias (allocation concealment)** | **Performance bias** | **Detection bias** | **Attrition bias** | **Reporting bias** | **Other bias** |
| --- | --- | --- | --- | --- | --- | --- | --- |
| **MET** |  |  |  |  |  |  |  |
| Anderson, 2017(10) | Low risk | Low risk | Low risk | Low risk | Low risk | Low risk | Low risk |
| Codner, 2013(11) | Low risk | Low risk | Low risk | Low risk | Low risk | Low risk | Low risk |
| Hamilton, 2003(12) | Low risk | Low risk | Low risk | Low risk | Low risk | Low risk | Low risk |
| Jacobsen, 2008(13) | Unclear risk | Unclear risk | Low risk | Low risk | Low risk | Low risk | Low risk |
| Lund, 2008(14) | Unclear risk | Unclear risk | Low risk | Low risk | Low risk | Low risk | Low risk |
| Lund, 2009(15) | Unclear risk | Unclear risk | Low risk | Low risk | Low risk | Low risk | Low risk |
| Libman, 2015(16) | Low risk | Low risk | Low risk | Low risk | Low risk | Low risk | Low risk |
| Meyer, 2002(17) | Unclear risk | High risk | Low risk | Low risk | High risk | Low risk | Unclear risk |
| Nadeau, 2016(18) | Unclear risk | Low risk | Low risk | Low risk | Low risk | Low risk | Low risk |
| Nwosu, 2015(19) | Unclear risk | Low risk | Low risk | Low risk | Low risk | Low risk | Low risk |
| Petrie, 2016(20) | Low risk | Low risk | Low risk | Low risk | Low risk | Low risk | Low risk |
| Petrie, 2017(21) | Low risk | Low risk | Low risk | Low risk | Low risk | Low risk | Low risk |
| Pitocco, 2013(22) | Unclear risk | Unclear risk | Low risk | Low risk | Low risk | Low risk | Low risk |
| Sarnblad, 2003(23) | High risk | High risk | Low risk | Low risk | Low risk | Low risk | Unclear risk |
| Ziaee, 2017(24) | Unclear risk | Unclear risk | Low risk | Low risk | Low risk | Low risk | Low risk |
| **AGI** |  |  |  |  |  |  |  |
| Hollander, 1997(25) | Unclear risk | Unclear risk | Low risk | Low risk | High risk | Low risk | Unclear risk |
| Riccardi, 1999(26) | Unclear risk | Unclear risk | Low risk | Low risk | High risk | Low risk | Unclear risk |
| **TZD** |  |  |  |  |  |  |  |
| Bhat, 2007(27) | Unclear risk | Unclear risk | Low risk | Low risk | Low risk | Low risk | Unclear risk |
| Strowig, 2005(28) | High risk | High risk | Low risk | Low risk | Low risk | Low risk | Unclear risk |
| Tafuri, 2013(29) | Unclear risk | Unclear risk | Low risk | Low risk | Low risk | Low risk | Unclear risk |
| Zdravkovic, 2006(30) | Low risk | Low risk | Low risk | Low risk | Low risk | Low risk | Low risk |
| **GLP-1 RA** |  |  |  |  |  |  |  |
| Ahren,2016(31) | Low risk | Low risk | Low risk | Low risk | Low risk | Low risk | Low risk |
| Dejgaard,2015(32) | Low risk | Low risk | Low risk | Low risk | Low risk | Low risk | Low risk |
| Dejgaard,2016(33) | Low risk | Low risk | Low risk | Low risk | Low risk | Low risk | Low risk |
| Fradsen, 2015(34) | Low risk | Low risk | Low risk | Low risk | Low risk | Low risk | Low risk |
| Fradsen, 2017(35) | Low risk | Low risk | Low risk | Low risk | Low risk | Low risk | Low risk |
| Kuhadiya, 2016(36) | Low risk | Low risk | Low risk | Low risk | Low risk | Low risk | Low risk |
| Mathieu, 2016(37) | Low risk | Low risk | Low risk | Low risk | Low risk | Low risk | Low risk |
| **DPP-4 inhibitor** |  |  |  |  |  |  |  |
| Garg, 2013(38) | Unclear risk | Low risk | Low risk | Low risk | Low risk | Low risk | Unclear risk |
| **SGLT2 inhibitor** |  |  |  |  |  |  |  |
| Buse, 2018(39) | Low risk | Low risk | Low risk | Low risk | Low risk | Low risk | Low risk |
| Dandona,2017(40) | Low risk | Low risk | Low risk | Low risk | Low risk | Low risk | Low risk |
| Danne, 2018(41) | Low risk | Low risk | Low risk | Low risk | Low risk | Low risk | Low risk |
| Famulla, 2016(42) | Low risk | Low risk | Low risk | Low risk | Low risk | Low risk | Low risk |
| Pieber, 2015(43) | Low risk | Low risk | Low risk | Low risk | Low risk | Low risk | Low risk |
| Garg, 2017(44) | Low risk | Low risk | Low risk | Low risk | Low risk | Low risk | Low risk |
| Henry, 2015(45) | Low risk | Low risk | Low risk | Low risk | Low risk | Low risk | Low risk |
| Rodbard, 2016(46) | Low risk | Low risk | Low risk | Low risk | Low risk | Low risk | Low risk |
| Peters, 2016(47) | Low risk | Low risk | Low risk | Low risk | Low risk | Low risk | Low risk |
| Kuhadjya, 2016(48) | Low risk | Low risk | Low risk | Low risk | Low risk | Low risk | Unclear risk |
| Sands, 2015(49) | Low risk | Low risk | Low risk | Low risk | Low risk | Low risk | Unclear risk |
| Shimada, 2018(50) | Low risk | Low risk | Low risk | Low risk | Low risk | Low risk | Low risk |
| Mathieu, 2018(51) | Low risk | Low risk | Low risk | Low risk | Low risk | Low risk | Low risk |
| Rosenstock, 2018(52) | Low risk | Low risk | Low risk | Low risk | Low risk | Low risk | Low risk |
| **Pramlintide** |  |  |  |  |  |  |  |
| Edelman, 2006(53) | High risk | Unclear risk | Low risk | Low risk | High risk | Low risk | Unclear risk |
| Marrero, 2007(54) | Unclear risk | Unclear risk | Low risk | Low risk | Low risk | Low risk | Low risk |
| Kovatchev, 2008(55) | Unclear risk | Unclear risk | Low risk | Low risk | Low risk | Low risk | Low risk |
| Hermann, 2013(56) | Unclear risk | Unclear risk | Low risk | Low risk | Low risk | Low risk | Unclear risk |
| Ratner, 2004(57) | High risk | High risk | Low risk | Low risk | Low risk | Low risk | Unclear risk |
| Ratner, 2005(58) | High risk | Unclear risk | Low risk | Low risk | Low risk | Low risk | Unclear risk |
| Whitehouse, 2002(59) | Unclear risk | Unclear risk | Low risk | Low risk | High risk | Low risk | Unclear risk |

**Table S4. Risk of bias of included RCT studies with type 2 diabetes**

|  | **Selection bias (random sequence generation)** | | **Selection bias (allocation concealment)** | **Performance bias** | **Detection bias** | **Attrition bias** | **Reporting bias** | **Other bias** |
| --- | --- | --- | --- | --- | --- | --- | --- | --- |
| **Exclusive PBO effect (monotherapy)** | | | | | | | | |
| **SU versus PBO** |  | |  |  |  |  |  |  |
| Madsbad, 2004(60) | | Low risk | Low risk | Low risk | Low risk | Low risk | Low risk | Unclear risk |
| Scott, 2007(61) | | Low risk | Low risk | Unclear risk | Low risk | Low risk | Low risk | Unclear risk |
| Goldberg, 1996(62) | | Low risk | Low risk | Unclear risk | Low risk | Low risk | Low risk | Low risk |
| Rosenstock, 1996(63) | | Low risk | Low risk | Low risk | Low risk | Low risk | Low risk | Low risk |
| Simonson, 1997(64) | | Unclear risk | Unclear risk | Unclear risk | Low risk | Low risk | Low risk | Low risk |
| Fischer, 2003(65) | | Low risk | Low risk | Low risk | Low risk | High risk | Unclear risk | Unclear risk |
| Hanefeld, 2002(66) | | High risk | Unclear risk | Unclear risk | Low risk | Low risk | Unclear risk | Unclear risk |
| Hoffmann, 1994(67) | | Low risk | Low risk | Low risk | Low risk | High risk | Low risk | Unclear risk |
| Segal, 1997(68) | | Low risk | Low risk | Low risk | Low risk | High risk | Low risk | Unclear risk |
| Ebeling, 2001(69) | | Low risk | Low risk | Low risk | Low risk | High risk | Low risk | Unclear risk |
| Coniff, 1995(70) | | Low risk | Low risk | Low risk | Low risk | High risk | Low risk | Unclear risk |
| **MET versus PBO** | |  |  |  |  |  |  |  |
| Johnson, 1993(71) | | Low risk | Low risk | Low risk | Low risk | High risk | Low risk | Unclear risk |
| Tessari, 1994(72) | | High risk | High risk | Low risk | Low risk | High risk | Low risk | Unclear risk |
| List, 2009(73) | | Unclear risk | Unclear risk | Low risk | Low risk | Low risk | Low risk | Low risk |
| Fonseca, 2013(74) | | Low risk | Low risk | Low risk | Low risk | Low risk | Low risk | Unclear risk |
| Garber, 1997(75) | | Unclear risk | Unclear risk | Low risk | Low risk | Low risk | Low risk | Unclear risk |
| Natali, 2004(76) | | Low risk | Low risk | Unclear risk | Low risk | Low risk | Unclear risk | Unclear risk |
| Fujioka, 2005(77) | | Low risk | Low risk | Low risk | Low risk | Low risk | Low risk | Unclear risk |
| Hoffmann, 1997(78) | | Low risk | Low risk | Low risk | Low risk | High risk | Low risk | Unclear risk |
| Horton, 2000(79) | | Low risk | Low risk | Low risk | Low risk | Low risk | Low risk | Unclear risk |
| Goldstein, 2007(80) | | Low risk | Low risk | Low risk | Low risk | Low risk | Low risk | Low risk |
| Haak, 2012(81) | | Low risk | Low risk | Low risk | Low risk | Low risk | Low risk | Unclear risk |
| Hällsten, 2002(82) | | Low risk | Low risk | Low risk | Low risk | High risk | Low risk | Unclear risk |
| Viljanen, 2005(83) | | Unclear risk | Unclear risk | Low risk | Low risk | Unclear risk | Low risk | Low risk |
| DeFronzo, 1995(84) | | Low risk | Low risk | Low risk | Low risk | High risk | Low risk | Unclear risk |
| Dornan, 1991(85) | | Low risk | Low risk | Unclear risk | Unclear risk | Low risk | Low risk | Unclear risk |
| Chiasson, 2001(86) | | Low risk | Low risk | Low risk | Low risk | Low risk | Low risk | Unclear risk |
| **AGI versus PBO** | |  |  |  |  |  |  |  |
| Wagner, 2006(87) | | High risk | High risk | Unclear risk | Low risk | Low risk | Unclear risk | Unclear risk |
| Coniff, 1995(88) | | Low risk | Low risk | Low risk | Low risk | Unclear risk | Low risk | Unclear risk |
| Calle-Pascuac, 1996(89) | | Low risk | Low risk | Low risk | Low risk | Low risk | Unclear risk | Unclear risk |
| Scott, 1999(90) | | Low risk | Low risk | Low risk | Low risk | Unclear risk | Low risk | Low risk |
| Hanefeld, 2002(66) | | High risk | Unclear risk | Unclear risk | Low risk | Low risk | Unclear risk | Unclear risk |
| Fischer, 2003(65) | | Low risk | Low risk | Low risk | Low risk | High risk | Unclear risk | Unclear risk |
| Delgado, 2002(91) | | Low risk | Low risk | Low risk | Low risk | Unclear risk | Unclear risk | Unclear risk |
| Hanefeld, 2009(92) | | Low risk | Low risk | Low risk | Low risk | Unclear risk | Low risk | Low risk |
| Rosenbaum, 2002(93) | | Low risk | Low risk | Low risk | Low risk | Low risk | Low risk | Low risk |
| Hotta, 1993(94) | | High risk | High risk | Low risk | Low risk | Low risk | Low risk | Unclear risk |
| Hoffmann, 1994(67) | | Low risk | Low risk | Low risk | Low risk | High risk | Low risk | Unclear risk |
| Hoffmann, 1997(78) | | Low risk | Low risk | Low risk | Low risk | High risk | Low risk | Unclear risk |
| Segal, 1997(68) | | Low risk | Low risk | Low risk | Low risk | High risk | Low risk | Unclear risk |
| Chan, 1998(95) | | Low risk | Low risk | Low risk | Low risk | Low risk | Low risk | Low risk |
| Gentile, 2001(96) | | Low risk | Low risk | Low risk | Low risk | Low risk | Unclear risk | Unclear risk |
| Coniff, 1994(97) | | Low risk | Low risk | Low risk | Low risk | Low risk | Low risk | Low risk |
| Coniff, 1995(70) | | Low risk | Low risk | Low risk | Low risk | High risk | Low risk | Unclear risk |
| Chiasson, 2001(86) | | Low risk | Low risk | Low risk | Low risk | Low risk | Low risk | Unclear risk |
| Chiasson, 1994(98) | | Low risk | Low risk | Low risk | Low risk | Unclear risk | Low risk | Unclear risk |
| Meneilly, 2000(99) | | Low risk | Low risk | Low risk | Low risk | High risk | Unclear risk | Unclear risk |
| Josse, 2003(100) | | Low risk | Low risk | Low risk | Low risk | High risk | Low risk | Unclear risk |
| Hasche, 1999(101) | | Low risk | Low risk | Low risk | Low risk | Low risk | Low risk | Low risk |
| **TZD versus PBO** | |  |  |  |  |  |  |  |
| Iwamoto, 1996(102) | | Low risk | Low risk | Low risk | Low risk | High risk | Low risk | Low risk |
| Kumar, 1996(103) | | Low risk | Low risk | Low risk | Low risk | High risk | Low risk | Low risk |
| Patel, 1999(104) | | Low risk | Low risk | Unclear risk | Unclear risk | High risk | Low risk | Low risk |
| Raskin, 2000(105) | | Low risk | Low risk | Low risk | Low risk | Low risk | Low risk | Low risk |
| Miyazaki, 2001(106) | | Low risk | Low risk | Low risk | Low risk | High risk | Low risk | Low risk |
| Juhl, 2003(107) | | Low risk | Low risk | Low risk | Low risk | Low risk | Low risk | Low risk |
| Wallace, 2004(108) | | Low risk | Unclear risk | Low risk | Low risk | High risk | Low risk | Low risk |
| Gastaldelli, 2006(109) | | Low risk | Low risk | Low risk | Low risk | High risk | Low risk | Low risk |
| Sourij, 2006(110) | | Low risk | Low risk | Low risk | Low risk | High risk | Low risk | Low risk |
| Oz Gul, 2008(111) | | Low risk | Low risk | Low risk | Low risk | High risk | Low risk | Low risk |
| Oz Gul, 2010(112) | | Low risk | Low risk | Low risk | Low risk | Low risk | Low risk | Low risk |
| Kong, 2011(113) | | Low risk | Low risk | Low risk | Low risk | Low risk | Low risk | Low risk |
| Colca, 2013(114) | | Low risk | Low risk | Low risk | Low risk | Low risk | Low risk | Low risk |
| Ebeling, 1999(115) | | Low risk | Low risk | Low risk | Low risk | High risk | Low risk | Low risk |
| Miyazaki, 2001(116) | | Unclear risk | Unclear risk | Low risk | Low risk | Unclear risk | Low risk | Low risk |
| Phillips, 2001(117) | | Unclear risk | Unclear risk | Low risk | Low risk | Unclear risk | Low risk | Low risk |
| Rosenblatt, 2001(118) | | Low risk | Low risk | Low risk | Low risk | Low risk | Low risk | Low risk |
| Carey, 2002(119) | | Low risk | Low risk | Low risk | Low risk | Low risk | Low risk | Low risk |
| Rosenstock, 2002(120) | | Low risk | Low risk | Low risk | Low risk | Low risk | Low risk | Low risk |
| Natali, 2004(76) | | Low risk | Low risk | Unclear risk | Low risk | Low risk | Unclear risk | Unclear risk |
| Lautamäki, 2005(121) | | Low risk | Low risk | Low risk | Low risk | Low risk | Low risk | Low risk |
| Gastaldelli, 2007-1(122) | | Low risk | Low risk | Low risk | Low risk | Low risk | Low risk | Low risk |
| Tan, 2005(123) | | Low risk | Low risk | Low risk | Low risk | Unclear risk | Low risk | Low risk |
| Miyazaki, 2002(124) | | Low risk | Low risk | Low risk | Low risk | Low risk | Low risk | Low risk |
| Fonseca, 1998(125) | | Low risk | Low risk | Low risk | Low risk | Low risk | Low risk | Low risk |
| Fonseca, 1998(126) | | Low risk | Low risk | Low risk | Low risk | Low risk | Low risk | Low risk |
| Aronoff, 2000(127) | | Low risk | Low risk | Low risk | Low risk | Low risk | Low risk | Low risk |
| Haffner, 2002(128) | | Low risk | Low risk | Low risk | Low risk | High risk | Low risk | Low risk |
| Hällsten, 2002(82) | | Low risk | Low risk | Low risk | Low risk | High risk | Low risk | Unclear risk |
| Scherbaum, 2002(129) | | Low risk | Low risk | Low risk | Low risk | Low risk | Low risk | Low risk |
| Viljanen, 2005(83) | | Unclear risk | Unclear risk | Low risk | Low risk | Unclear risk | Low risk | Low risk |
| Khan, 2006(130) | | Low risk | Unclear risk | Low risk | Low risk | Unclear risk | Low risk | Low risk |
| Truitt, 2010(131) | | Low risk | Low risk | Low risk | Low risk | Low risk | Low risk | Low risk |
| Chou, 2012(132) | | Low risk | Low risk | Low risk | Low risk | Low risk | Low risk | Low risk |
| Ebeling, 2001(69) | | Low risk | Low risk | Low risk | Low risk | High risk | Low risk | Unclear risk |
| **DPP-4 inhibitor versus PBO** | |  |  |  |  |  |  |  |
| Ristic, 2005(133) | | Low risk | Low risk | Low risk | Low risk | Low risk | Low risk | Unclear risk |
| Hanefeld, 2007(134) | | Low risk | Low risk | Low risk | Low risk | Low risk | Low risk | Low risk |
| Scott, 2007(61) | | Low risk | Low risk | Unclear risk | Low risk | Low risk | Low risk | Unclear risk |
| Nonaka, 2008(135) | | Low risk | Low risk | Low risk | Low risk | Low risk | Low risk | Low risk |
| Rosenstock, 2008(136) | | Low risk | Low risk | Low risk | Low risk | Low risk | Low risk | Unclear risk |
| Kikuchi, 2009(137) | | Low risk | Low risk | Low risk | Low risk | Low risk | Low risk | Low risk |
| Iwamoto, 2010(138) | | Low risk | Low risk | Low risk | Low risk | Low risk | Low risk | Low risk |
| Pattzi, 2010(139) | | Low risk | Low risk | Low risk | Low risk | Low risk | Low risk | Unclear risk |
| cRhee, 2010(140) | | Low risk | Low risk | Low risk | Low risk | Low risk | Low risk | Unclear risk |
| Seino, 2011(141) | | Low risk | Low risk | Low risk | Low risk | Low risk | Low risk | Low risk |
| Kawamori, 2012(142) | | Low risk | Low risk | Low risk | Low risk | Low risk | Low risk | Low risk |
| Kadowaki, 2013(143) | | Low risk | Low risk | Low risk | Low risk | Low risk | Low risk | Low risk |
| Inagaki, 2014(144) | | Low risk | Low risk | Low risk | Low risk | Low risk | Low risk | Low risk |
| Sheu,2015(145) | | Low risk | Low risk | Low risk | Low risk | Unclear risk | Low risk | Low risk |
| Yoon, 2017(146) | | Low risk | Low risk | Low risk | Low risk | Unclear risk | Low risk | Low risk |
| Pan 2015(147) | | Low risk | Low risk | Low risk | Low risk | Low risk | Low risk | Low risk |
| Agarwal, 2018(148) | | Low risk | Low risk | Low risk | Low risk | Low risk | Low risk | Unclear risk |
| Raz, 2006(149) | | Low risk | Low risk | Low risk | Low risk | Low risk | Low risk | Unclear risk |
| Mohan, 2009(150) | | Low risk | Low risk | Low risk | Low risk | Low risk | Low risk | Low risk |
| Aschner, 2006(151) | | Low risk | Low risk | Low risk | Low risk | Low risk | Low risk | Low risk |
| Dejager, 2007(152) | | Low risk | Low risk | Low risk | Low risk | Low risk | Low risk | Low risk |
| Goldstein, 2007(80) | | Low risk | Low risk | Low risk | Low risk | Low risk | Low risk | Low risk |
| Pi-Sunyer, 2007(153) | | Low risk | Low risk | Low risk | Low risk | Low risk | Low risk | Low risk |
| Rosenstock, 2009(154) | | Low risk | Low risk | Low risk | Low risk | Low risk | Low risk | Unclear risk |
| Del Prato, 2011(155) | | Low risk | Low risk | Low risk | Low risk | Low risk | Low risk | Low risk |
| Pan, 2012(156) | | Low risk | Low risk | Low risk | Low risk | Low risk | Low risk | Low risk |
| Frederich, 2012(157) | | Low risk | Low risk | Low risk | Low risk | Low risk | Low risk | Low risk |
| Haak, 2012(81) | | Low risk | Low risk | Low risk | Low risk | Low risk | Low risk | Unclear risk |
| Yang, 2012(158) | | Low risk | Low risk | Low risk | Low risk | Low risk | Low risk | Low risk |
| Roden, 2013(159) | | Low risk | Low risk | Unclear risk | Low risk | Low risk | Low risk | Unclear risk |
| Inagaki, 2015(160) | | Unclear risk | Low risk | Low risk | Low risk | Unclear risk | Low risk | Unclear risk |
| Wu 2015(161) | | Low risk | Low risk | Low risk | Low risk | Low risk | Low risk | Low risk |
| Hong, 2016(162) | | Low risk | Low risk | Low risk | Low risk | Low risk | Low risk | Unclear risk |
| Ji 2016(163) | | Low risk | Low risk | Low risk | Unclear risk | Low risk | Low risk | Low risk |
| Chacra, 2017(164) | | Low risk | Low risk | Low risk | Low risk | Low risk | Low risk | Unclear risk |
| Home, 2018(165) | | Low risk | Low risk | Low risk | Unclear risk | Low risk | Low risk | Unclear risk |
| Gantz, 2017(166) | | Low risk | Low risk | Low risk | Low risk | Low risk | Low risk | Low risk |
| Prately,2014(167) | | Low risk | Low risk | Low risk | Low risk | Low risk | Low risk | Low risk |
| Ji, 2017(168) | | Low risk | Low risk | Low risk | Low risk | Low risk | Low risk | Low risk |
| Scherbaum, 2008(169) | | Low risk | Low risk | Low risk | Low risk | Low risk | Low risk | Unclear risk |
| **SGLT2i versus PBO** | |  |  |  |  |  |  |  |
| Ott, 2017(170) | | Low risk | Low risk | Low risk | Low risk | Low risk | Low risk | Low risk |
| List, 2009(73) | | Unclear risk | Unclear risk | Low risk | Low risk | Low risk | Low risk | Low risk |
| Ferrannini, 2013(171) | | Low risk | Low risk | Low risk | Low risk | Low risk | Low risk | Low risk |
| Fonseca, 2013(74) | | Low risk | Low risk | Low risk | Low risk | Low risk | Low risk | Unclear risk |
| Inagaki, 2013(172) | | Low risk | Low risk | Low risk | Low risk | Low risk | Low risk | Low risk |
| Kaku, 2013(173) | | Low risk | Low risk | Low risk | Low risk | Low risk | Low risk | Unclear risk |
| Seino, 2014-2(174) | | Low risk | Low risk | Low risk | Low risk | Low risk | Low risk | Low risk |
| Seino, 2014-3(175) | | Low risk | Low risk | Low risk | Low risk | Low risk | Low risk | Low risk |
| Ikeda, 2015(176) | | Low risk | Low risk | Low risk | Low risk | Low risk | Low risk | Low risk |
| Sykes, 2015(177) | | Low risk | Low risk | Low risk | Low risk | Low risk | Low risk | Low risk |
| Sykes2015-1(178) | | Low risk | Low risk | Low risk | Low risk | Low risk | Low risk | Low risk |
| Heerspink,2016(179) | | Low risk | Low risk | Low risk | Low risk | Low risk | Low risk | Unclear risk |
| Ferrannini, 2010(180) | | Low risk | Low risk | Low risk | Low risk | Low risk | Low risk | Unclear risk |
| Bailey, 2012(181) | | Low risk | Low risk | Low risk | Low risk | Low risk | Low risk | Low risk |
| Roden, 2013(159) | | Low risk | Low risk | Unclear risk | Low risk | Low risk | Low risk | Unclear risk |
| Inagaki, 2014(182) | | Low risk | Low risk | Low risk | Low risk | Low risk | Low risk | Low risk |
| Kaku, 2014(183) | | Unclear risk | Unclear risk | Low risk | Unclear risk | Low risk | Low risk | Low risk |
| Ji, 2014(184) | | Low risk | Low risk | Low risk | Low risk | Low risk | Low risk | Low risk |
| Kashiwagi, 2015(185) | | Low risk | Low risk | Low risk | Low risk | Low risk | Low risk | Low risk |
| Kashiwagi, 2015(186) | | Low risk | Low risk | Low risk | Low risk | Low risk | Low risk | Low risk |
| Seino, 2014-1 (187) | | Low risk | Low risk | Low risk | Low risk | Low risk | Low risk | Low risk |
| Seino, 2015(188) | | Low risk | Low risk | Low risk | Low risk | Low risk | Low risk | Low risk |
| Haneda, 2016(189) | | Low risk | Low risk | Unclear risk | Low risk | Low risk | Low risk | Low risk |
| Li, 2016(190) | | Low risk | Low risk | Low risk | Low risk | Low risk | Low risk | Low risk |
| Pollock, 2019[(191)](#_ENREF_132) | | Low risk | Low risk | Low risk | Low risk | Low risk | Low risk | Low risk |
| Stenlöf, 2013(192) | | Low risk | Low risk | Low risk | Low risk | Low risk | Low risk | Unclear risk |
| Terra,2017(193) | | Low risk | Low risk | Low risk | Low risk | Low risk | Low risk | Low risk |
| Aronson, 2018(194) | | Low risk | Low risk | Low risk | Low risk | Low risk | Low risk | Low risk |
| **Augmented PBO effect (add-on therapy)** | | | | | | | | |
| **SU versus PBO** | |  |  |  |  |  |  |  |
| Lins, 1988(195) | | Low risk | Low risk | Low risk | Low risk | Low risk | Low risk | Low risk |
| Stuart, 1997(196) | | High risk | High risk | Low risk | Unclear risk | Low risk | Low risk | Low risk |
| Forst, 2010(197) | | Low risk | Low risk | Low risk | Low risk | Low risk | Low risk | Low risk |
| Burant, 2012(198) | | Low risk | Low risk | Low risk | Low risk | Low risk | Low risk | Low risk |
| Schade, 1987(199) | | Low risk | Low risk | Low risk | Low risk | Low risk | Low risk | Low risk |
| Stenman, 1988(200) | | Low risk | Low risk | Low risk | Low risk | High risk | Low risk | Low risk |
| Riddle, 1992(201) | | Low risk | Low risk | Low risk | Low risk | High risk | Low risk | Low risk |
| Feinglos, 2005(202) | | Low risk | Low risk | Low risk | Low risk | Low risk | Low risk | Low risk |
| Lewitt, 1989(203) | | High risk | High risk | Low risk | Low risk | High risk | Low risk | Low risk |
| Riddle, 1998(204) | | Low risk | Low risk | Low risk | Low risk | Low risk | Low risk | Low risk |
| Roberts, 2005(205) | | Low risk | Low risk | Low risk | Low risk | Low risk | Low risk | Low risk |
| Nauck, 2009(206) | | Low risk | Low risk | Unclear risk | Low risk | Low risk | Low risk | Low risk |
| Karlander, 1991(207) | | Low risk | Low risk | Low risk | Low risk | High risk | Low risk | Low risk |
| Camerini-Davalos, 1994(208) | | Low risk | Low risk | Low risk | Low risk | Low risk | Low risk | Low risk |
| **MET versus PBO** | |  |  |  |  |  |  |  |
| Willms, 1999(209) | | Low risk | Low risk | Low risk | Low risk | Low risk | Low risk | Unclear risk |
| Avilés-Santa, 1999(210) | | Low risk | Low risk | Low risk | Low risk | Low risk | Low risk | Low risk |
| Chiasson, 2001(86) | | Low risk | Low risk | Low risk | Low risk | Low risk | Low risk | Unclear risk |
| Hermann, 2001(211) | | Low risk | Low risk | Low risk | Low risk | High risk | Low risk | Unclear risk |
| Douek, 2005(212) | | Low risk | Low risk | Low risk | Low risk | Low risk | Low risk | Low risk |
| Gram, 2011(213) | | Low risk | Low risk | Unclear risk | Low risk | Low risk | Unclear risk | Unclear risk |
| Kooy, 2009(214) | | Low risk | Unclear risk | Unclear risk | Low risk | Low risk | Low risk | Unclear risk |
| **AGI versus PBO** | |  |  |  |  |  |  |  |
| Willms, 1999(209) | | Low risk | Low risk | Low risk | Low risk | Low risk | Low risk | Unclear risk |
| Nemoto, 2011(215) | | Low risk | Low risk | Low risk | Low risk | Unclear risk | Low risk | Low risk |
| Hwu, 2003(216) | | Low risk | Low risk | Low risk | Low risk | Low risk | Low risk | Low risk |
| Schnell, 2007(217) | | Low risk | Low risk | Low risk | Low risk | Low risk | Low risk | Low risk |
| Kelley, 1998(218) | | Low risk | Low risk | Low risk | Low risk | High risk | Low risk | Unclear risk |
| Lam KSL, 1998(219) | | Low risk | Low risk | Low risk | Low risk | Unclear risk | Low risk | Unclear risk |
| Mitrakou,1998(220) | | Low risk | Low risk | Low risk | Low risk | High risk | Low risk | Low risk |
| Standl, 1999(221) | | Unclear risk | Unclear risk | Low risk | Low risk | Low risk | Low risk | Low risk |
| Standl, 2001(222) | | Low risk | Low risk | Low risk | Low risk | Unclear risk | Low risk | Low risk |
| Lin BJ, 2003(223) | | Unclear risk | Unclear risk | Low risk | Low risk | Unclear risk | Low risk | Low risk |
| Phillips, 2003(224) | | Low risk | Low risk | Low risk | Low risk | Low risk | Low risk | Low risk |
| Hsieh, 2011(225) | | Low risk | Low risk | Low risk | Low risk | Low risk | Low risk | Low risk |
| Halimi, 2000(226) | | Low risk | Low risk | Low risk | Low risk | High risk | Low risk | Low risk |
| Van Gaal, 2001(227) | | Low risk | Low risk | Low risk | Low risk | Low risk | Low risk | Low risk |
| Chiasson, 2001(86) | | Low risk | Low risk | Low risk | Low risk | Low risk | Low risk | Unclear risk |
| Chiasson-1, 1994(98) | | Low risk | Low risk | Low risk | Low risk | Unclear risk | Low risk | Unclear risk |
| Chiasson-2, 1994(98) | | Low risk | Low risk | Low risk | Low risk | Unclear risk | Low risk | Unclear risk |
| Chiasson-3, 1994(98) | | Low risk | Low risk | Low risk | Low risk | Unclear risk | Low risk | Unclear risk |
| Johnston, 1998 (228) | | Low risk | Low risk | Low risk | Low risk | High risk | Low risk | Unclear risk |
| Johnston, 1998-2(229) | | Low risk | Low risk | Low risk | Low risk | High risk | Low risk | Unclear risk |
| Bachmann, 2003(230) | | Low risk | Low risk | Low risk | Low risk | Low risk | Low risk | Low risk |
| **TZD versus PBO** | |  |  |  |  |  |  |  |
| Iwamoto, 1996(231) | | Low risk | Low risk | Low risk | Low risk | Unclear risk | Low risk | Low risk |
| Kawamori, 1998(232) | | Low risk | Low risk | Low risk | Low risk | Low risk | Low risk | Low risk |
| Kelly, 1999(233) | | Low risk | Low risk | Low risk | Low risk | High risk | Low risk | Low risk |
| Pan, 2002(234) | | Low risk | Low risk | Low risk | Low risk | High risk | Low risk | Low risk |
| Buras, 2005(235) | | High risk | Unclear risk | Low risk | Low risk | Unclear risk | Low risk | Low risk |
| Mimura, 1994(236) | | Low risk | Low risk | Low risk | Low risk | High risk | Low risk | Low risk |
| Osende, 2001(237) | | Low risk | Low risk | Low risk | Low risk | Unclear risk | Low risk | Low risk |
| Brackenridge, 2009(238) | | Low risk | Low risk | Low risk | Low risk | Low risk | Unclear risk | Low risk |
| Buysschaert, 1999(239) | | Low risk | Low risk | Low risk | Low risk | Low risk | Low risk | Low risk |
| Kipnes, 2001(240) | | Low risk | Low risk | Low risk | Low risk | Unclear risk | Low risk | Low risk |
| Scott, 2008(241) | | Low risk | Low risk | Low risk | Low risk | Low risk | Low risk | Unclear risk |
| Gastaldelli, 2007-2([122](#_ENREF_63)) | | Low risk | Low risk | Low risk | Low risk | Low risk | Low risk | Low risk |
| Berhanu, 2007(242) | | Low risk | Low risk | Low risk | Low risk | Low risk | Low risk | Low risk |
| Yale, 2001(243) | | Low risk | Low risk | Low risk | Low risk | Unclear risk | Low risk | Low risk |
| Zhu, 2003(244) | | Low risk | Low risk | Low risk | Low risk | Low risk | Low risk | Low risk |
| Dailey, 2004(245) | | Low risk | Low risk | Low risk | Low risk | Low risk | Low risk | Low risk |
| Smith, 2005(246) | | Low risk | Low risk | Low risk | Low risk | Unclear risk | Low risk | Low risk |
| Davidson, 2007(247) | | Low risk | Low risk | Low risk | Low risk | Low risk | Low risk | Low risk |
| Hollander, 2007(248) | | Low risk | Low risk | Low risk | Low risk | Low risk | Low risk | Low risk |
| Sridhar, 2013(249) | | Low risk | Low risk | Low risk | Low risk | Low risk | Low risk | Low risk |
| Buse, 1998(250) | | Low risk | Low risk | Low risk | Low risk | Low risk | Low risk | Low risk |
| Schwartz, 1998(251) | | Low risk | Low risk | Low risk | Low risk | High risk | Low risk | Low risk |
| Fonseca, 2000(252) | | Low risk | Low risk | Low risk | Low risk | Low risk | Low risk | Low risk |
| Wolffenbuttel, 2000(253) | | Low risk | Low risk | Low risk | Low risk | High risk | Low risk | Low risk |
| Raskin, 2001(254) | | Low risk | Low risk | Low risk | Low risk | Low risk | Low risk | Low risk |
| Barnett, 2003(255) | | Low risk | Low risk | Low risk | Low risk | Low risk | Low risk | Low risk |
| Rosenstock, 2008(256) | | Low risk | Low risk | Low risk | Low risk | Low risk | Low risk | Low risk |
| Marre, 2009(257) | | Low risk | Low risk | Low risk | Low risk | Low risk | Low risk | Low risk |
| Henriksen, 2011(258) | | Low risk | Low risk | Low risk | Low risk | Low risk | Low risk | Low risk |
| Nakamura, 2001(259) | | Low risk | Low risk | Low risk | Low risk | Unclear risk | Low risk | Low risk |
| Reynolds, 2002(260) | | High risk | Unclear risk | Unclear risk | Low risk | Unclear risk | Low risk | Low risk |
| Yang, 2002(261) | | Low risk | Low risk | Low risk | Low risk | Low risk | Low risk | Low risk |
| Mattoo, 2005(262) | | Low risk | Low risk | Low risk | Low risk | Low risk | Low risk | Low risk |
| Derosa, 2008(263) | | Low risk | Low risk | Low risk | Low risk | Low risk | Low risk | Low risk |
| Galle, 2012(264) | | Low risk | Low risk | Low risk | Low risk | Low risk | Low risk | Low risk |
| Grey, 2012(265) | | Low risk | Low risk | Low risk | Low risk | Unclear risk | Low risk | Low risk |
| Kaku, 2009(266) | | Low risk | Low risk | Low risk | Low risk | Low risk | Low risk | Low risk |
| Charpentier, 2009(267) | | Low risk | Low risk | Low risk | Low risk | Low risk | Low risk | Low risk |
| Negro, 2005(268) | | Low risk | Low risk | Low risk | Low risk | Low risk | Low risk | Low risk |
| Bertrand, 2010(269) | | Low risk | Low risk | Low risk | Low risk | Low risk | Low risk | Low risk |
| Gram, 2011(213) | | Low risk | Low risk | Unclear risk | Low risk | Low risk | Unclear risk | Unclear risk |
| **DPP-4 inhibitor versus PBO** | |  |  |  |  |  |  |  |
| Ahrén, 2004(270) | | Low risk | Low risk | Low risk | Low risk | Low risk | Low risk | Unclear risk |
| Forst, 2010(197) | | Low risk | Low risk | Low risk | Low risk | Low risk | Low risk | Low risk |
| Kikuchi, 2010(271) | | Low risk | Low risk | Low risk | Low risk | Low risk | Low risk | Unclear risk |
| Kaku, 2011(272) | | Low risk | Low risk | Low risk | Low risk | Low risk | Low risk | Low risk |
| Nowicki, 2011(273) | | Low risk | Low risk | Low risk | Low risk | Low risk | Low risk | Low risk |
| Seino, 2011(274) | | Low risk | Low risk | Low risk | Low risk | Low risk | Low risk | Low risk |
| Rosenstock, 2012(275) | | Low risk | Low risk | Low risk | Low risk | Low risk | Low risk | Low risk |
| Ross, 2012(276) | | Low risk | Low risk | Low risk | Low risk | Low risk | Low risk | Low risk |
| Kadowaki, 2013(277) | | Low risk | Low risk | Unclear risk | Low risk | Low risk | Low risk | Unclear risk |
| Kadowaki, 2014(278) | | Low risk | Low risk | Low risk | Low risk | Low risk | Low risk | Low risk |
| Kim, 2014(279) | | Low risk | Low risk | Low risk | Low risk | Low risk | Low risk | Low risk |
| Kadowaki, 2017(280) | | Low risk | Low risk | Low risk | Low risk | Low risk | Low risk | Unclear risk |
| Pan, 2015(147) | | Low risk | Low risk | Low risk | Low risk | Low risk | Low risk | Low risk |
| Raz, 2008(281) | | Low risk | Low risk | Low risk | Low risk | Low risk | Low risk | Unclear risk |
| Scott, 2008(241) | | Low risk | Low risk | Low risk | Low risk | Low risk | Low risk | Unclear risk |
| Lewin, 2012(282) | | Low risk | Low risk | Low risk | Low risk | Low risk | Low risk | Low risk |
| Dobs, 2013(283) | | Low risk | Low risk | Low risk | Low risk | Low risk | Low risk | Low risk |
| Gantz, 2017(284) | | Low risk | Low risk | Low risk | Low risk | Low risk | Low risk | Low risk |
| Charbonnel, 2006(285) | | Low risk | Low risk | Low risk | Low risk | Low risk | Low risk | Low risk |
| Rosenstock, 2006(286) | | Low risk | Low risk | Low risk | Low risk | Low risk | Low risk | Unclear risk |
| Bosi, 2007(287) | | Low risk | Low risk | Low risk | Low risk | Low risk | Low risk | Unclear risk |
| Fonseca, 2007(288) | | Low risk | Low risk | Low risk | Low risk | Low risk | Low risk | Unclear risk |
| Garber, 2007(289) | | Low risk | Low risk | Low risk | Low risk | Low risk | Low risk | Unclear risk |
| Hermansen, 2007(290) | | Low risk | Low risk | Low risk | Low risk | Low risk | Low risk | Low risk |
| Garber, 2008(291) | | Low risk | Low risk | Low risk | Low risk | Low risk | Low risk | Unclear risk |
| Goodman, 2009(292) | | Low risk | Low risk | Low risk | Low risk | Low risk | Low risk | Unclear risk |
| Hollander, 2009(293) | | Low risk | Low risk | Low risk | Low risk | Low risk | Low risk | Low risk |
| Jadzinsky, 2009(294) | | Low risk | Low risk | Low risk | Low risk | Low risk | Low risk | Low risk |
| Pratley, 2009(295) | | Low risk | Low risk | Low risk | Low risk | Low risk | Low risk | Unclear risk |
| Vilsbøll, 2010(296) | | Low risk | Low risk | Low risk | Low risk | Low risk | Low risk | Unclear risk |
| Gomis, 2011(297) | | Low risk | Low risk | Low risk | Low risk | Low risk | Low risk | Low risk |
| Owens, 2011(298) | | Low risk | Low risk | Low risk | Low risk | Low risk | Low risk | Unclear risk |
| Taskinen, 2011(299) | | Low risk | Low risk | Low risk | Low risk | Low risk | Low risk | Low risk |
| Yang, 2011(300) | | Low risk | Low risk | Low risk | Low risk | Low risk | Low risk | Low risk |
| Barnett, 2012(301) | | Low risk | Low risk | Low risk | Low risk | Low risk | Low risk | Unclear risk |
| Bergenstal, 2012(302) | | Unclear risk | Unclear risk | Low risk | Low risk | Low risk | Low risk | Low risk |
| Haak, 2012(81) | | Low risk | Low risk | Low risk | Low risk | Low risk | Low risk | Unclear risk |
| Pan, 2012(303) | | Low risk | Low risk | Low risk | Low risk | Low risk | Low risk | Low risk |
| Seino, 2012(304) | | Low risk | Low risk | Low risk | Unclear risk | Unclear risk | Low risk | Unclear risk |
| Yang, 2012(305) | | Low risk | Low risk | Low risk | Low risk | Low risk | Low risk | Low risk |
| Barnett, 2013(306) | | Low risk | Low risk | Low risk | Low risk | Low risk | Low risk | Unclear risk |
| Strain, 2013(307) | | Low risk | Low risk | Low risk | Low risk | Unclear risk | Low risk | Low risk |
| Zeng 2013(308) | | Low risk | Low risk | Low risk | Low risk | Low risk | Low risk | Low risk |
| Lukashevic, 2014(309) | | Low risk | Low risk | Low risk | Low risk | Low risk | Low risk | Low risk |
| Wang 2015(310) | | Low risk | Low risk | Low risk | Low risk | Low risk | Low risk | Low risk |
| Yang, 2015(311) | | Low risk | Low risk | Low risk | Low risk | Low risk | Low risk | Low risk |
| Ning, 2016(312) | | Low risk | Low risk | Low risk | Low risk | Low risk | Low risk | Unclear risk |
| Ahn, 2017(313) | | Low risk | Low risk | Low risk | Low risk | Low risk | Low risk | Unclear risk |
| Lee, 2017(314) | | Low risk | Low risk | Low risk | Low risk | Low risk | Low risk | Unclear risk |
| Shankar, 2017(315) | | Low risk | Low risk | Unclear risk | Low risk | Low risk | Unclear risk | Low risk |
| Kadowaki, 2018(316) | | Low risk | Low risk | Low risk | Low risk | Low risk | Low risk | Low risk |
| Nauck, 2009(317) | | Low risk | Low risk | Low risk | Low risk | Low risk | Low risk | Unclear risk |
| DeFronzo, 2012(318) | | Low risk | Low risk | Low risk | Low risk | Low risk | Low risk | Unclear risk |
| Fonseca, 2013(319) | | Low risk | Low risk | Low risk | Low risk | Low risk | Low risk | Unclear risk |
| Lavalle-González, 2013(320) | | Low risk | Low risk | Low risk | Low risk | Low risk | Low risk | Low risk |
| Ji, 2017(168) | | Low risk | Low risk | Low risk | Low risk | Low risk | Low risk | Low risk |
| Derosa, 2012(321) | | Low risk | Low risk | Low risk | Low risk | Low risk | Low risk | Low risk |
| Derosa, 2012(322) | | Low risk | Low risk | Low risk | Low risk | Low risk | Low risk | Low risk |
| Bosi, 2011(323) | | Low risk | Low risk | Low risk | Low risk | Low risk | Low risk | Unclear risk |
| Kothny, 2012(324) | | Low risk | Low risk | Low risk | Low risk | Low risk | Low risk | Low risk |
| **SGLT2i versus PBO** | |  |  |  |  |  |  |  |
| Henry,2018(325) | | Low risk | Low risk | Low risk | Low risk | Low risk | Low risk | Low risk |
| Latva-Rasku, 2019(326) | | Low risk | Low risk | Low risk | Low risk | Low risk | Low risk | Low risk |
| Wilding, 2009(327) | | Low risk | Low risk | Low risk | Low risk | Low risk | Low risk | Low risk |
| Rosenstock, 2012(275) | | Low risk | Low risk | Low risk | Low risk | Low risk | Low risk | Low risk |
| Wilding, 2013(328) | | Low risk | Low risk | Low risk | Low risk | Low risk | Low risk | Low risk |
| Amin, 2015(329) | | Low risk | Low risk | Low risk | Low risk | Low risk | Low risk | Low risk |
| Weber,2016(330) | | Low risk | Low risk | Low risk | Low risk | Low risk | Low risk | Low risk |
| Kario,2018(331) | | Low risk | Low risk | Low risk | Low risk | Low risk | Low risk | Low risk |
| Ross, 2015(332) | | Low risk | Unclear risk | Unclear risk | Low risk | Low risk | Low risk | Low risk |
| Schumm-Draeger,2015(333) | | Low risk | Low risk | Low risk | Low risk | Low risk | Low risk | Low risk |
| Ishihara, 2016(334) | | Low risk | Low risk | Low risk | Low risk | Low risk | Low risk | Low risk |
| Terauchi, 2017(335) | | Low risk | Low risk | Low risk | Low risk | Low risk | Low risk | Low risk |
| Seino, 2018(336) | | Low risk | Low risk | Low risk | Low risk | Low risk | Low risk | Low risk |
| Ji,2015(337) | | Low risk | Low risk | Low risk | Low risk | Low risk | Low risk | Low risk |
| Neal, 2015(338) | | Low risk | Low risk | Low risk | Low risk | Low risk | Low risk | Unclear risk |
| Bailey, 2010(339) | | Low risk | Low risk | Low risk | Low risk | Low risk | Low risk | Low risk |
| Strojek, 2011(340) | | Low risk | Low risk | Low risk | Low risk | Low risk | Low risk | Low risk |
| Bolinder, 2012(341) | | Low risk | Low risk | Low risk | Low risk | Low risk | Low risk | Low risk |
| Henry, 2012(342) | | Low risk | Low risk | Low risk | Low risk | Low risk | Low risk | Low risk |
| Häring2013(343) | | Low risk | Low risk | Low risk | Unclear risk | Low risk | Low risk | Low risk |
| Häring, 2014(344) | | Low risk | Low risk | Low risk | Low risk | Low risk | Low risk | Low risk |
| Kovacs, 2014(345) | | Low risk | Low risk | Low risk | Low risk | Low risk | Low risk | Low risk |
| Leiter, 2014(346) | | Low risk | Low risk | Low risk | Low risk | Low risk | Low risk | Low risk |
| Mathieu, 2015(347) | | Low risk | Low risk | Low risk | Low risk | Low risk | Low risk | Low risk |
| Matthaei,2015(348) | | Low risk | Low risk | Low risk | Low risk | Low risk | Low risk | Unclear risk |
| Rosenstock, 2015(349) | | Low risk | Low risk | Low risk | Low risk | Low risk | Low risk | Low risk |
| Hadjadj, 2016(350) | | Low risk | Low risk | Low risk | Low risk | Low risk | Low risk | Low risk |
| Lu, 2016(351) | | Low risk | Low risk | Low risk | Low risk | Low risk | Low risk | Low risk |
| Kadowaki，2017(352) | | Low risk | Low risk | Low risk | Low risk | Low risk | Low risk | Low risk |
| Cusi, 2018(343) | | Unclear risk | Unclear risk | Low risk | Unclear risk | Low risk | Unclear risk | Low risk |
| Fioretto, 2018(354) | | Low risk | Low risk | Low risk | Low risk | Low risk | Low risk | Low risk |
| Han,2018(355) | | Low risk | Low risk | Low risk | Low risk | Low risk | Low risk | Low risk |
| Shestakova, 2018(356) | | Low risk | Low risk | Low risk | Low risk | Low risk | Low risk | Low risk |
| Yang, 2018(357) | | Low risk | Low risk | Low risk | Low risk | Low risk | Low risk | Low risk |
| Ferdinand, 2019(358) | | Low risk | Low risk | Low risk | Low risk | Low risk | Low risk | Low risk |
| Pollock, 2019(191) | | Low risk | Low risk | Low risk | Low risk | Low risk | Low risk | Low risk |
| Lavalle-González, 2013(320) | | Low risk | Low risk | Low risk | Low risk | Low risk | Low risk | Low risk |
| Yale, 2013(359) | | Low risk | Low risk | Low risk | Low risk | Low risk | Low risk | Low risk |
| Forst, 2014(360) | | Low risk | Low risk | Low risk | Low risk | Low risk | Low risk | Low risk |
| Grunberger, 2018(361) | | Low risk | Low risk | Low risk | Low risk | Low risk | Low risk | Low risk |
| Miller, 2018(362) | | Low risk | Low risk | Low risk | Low risk | Low risk | Low risk | Low risk |
| Rosenstock, 2018(363) | | Low risk | Low risk | Low risk | Low risk | Low risk | Low risk | Low risk |
| Ji, 2019(364) | | Low risk | Low risk | Low risk | Low risk | Low risk | Low risk | Low risk |
| Rosenstock, 2012(365) | | Low risk | Unclear risk | Low risk | Low risk | Low risk | Low risk | Low risk |
| Wilding, 2012(366) | | Low risk | Low risk | Low risk | Low risk | Low risk | Low risk | Low risk |
| Ljunggren, 2012(367) | | Low risk | Low risk | Low risk | Low risk | Low risk | Low risk | Low risk |
| Barnett, 2014(368) | | Low risk | Low risk | Low risk | Low risk | Low risk | Low risk | Low risk |
| Rosenstock, 2014(369) | | Low risk | Low risk | Low risk | Low risk | Low risk | Low risk | Low risk |
| Cefalu, 2015(370) | | Low risk | Low risk | Low risk | Low risk | Low risk | Low risk | Low risk |
| Neal, 2015 (338) | | Low risk | Low risk | Low risk | Low risk | Low risk | Low risk | Unclear risk |
| Araki, 2017(371) | | Low risk | Low risk | Low risk | Low risk | Low risk | Low risk | Low risk |
| Dagogo-Jack, 2018(372) | | Low risk | Low risk | Low risk | Low risk | Low risk | Low risk | Low risk |
| Yale, 2017(373) | | Low risk | Low risk | Low risk | Low risk | Low risk | Low risk | Low risk |
| Hattori, 2018(374) | | Low risk | Low risk | Low risk | Low risk | Low risk | Low risk | Low risk |
| Jabbour, 2018(375) | | Low risk | Low risk | Low risk | Low risk | Low risk | Low risk | Unclear risk |
| Kawamori, 2018(376) | | Low risk | Low risk | Low risk | Low risk | Low risk | Low risk | Low risk |
| Haering, 2015(377) | | Low risk | Low risk | Low risk | Low risk | Low risk | Low risk | Low risk |
| Merker, 2015(378) | | Low risk | Low risk | Low risk | Low risk | Low risk | Low risk | Low risk |
| Bode, 2015(379) | | Low risk | Low risk | Low risk | Low risk | Low risk | Low risk | Low risk |
| Perkovic, 2019(380) | | Low risk | Low risk | Low risk | Low risk | Low risk | Low risk | Low risk |
| Neal, 2017(381) | | Low risk | Low risk | Low risk | Low risk | Low risk | Low risk | Unclear risk |
| Wiviott, 2019(382) | | Low risk | Low risk | Low risk | Low risk | Low risk | Low risk | Low risk |
| **Exclusive PBO effect by injection** | | | | | | | | |
| **GLP-1RA vs PBO** | |  |  |  |  |  |  |  |
| Madsbad, 2004(60) | | Low risk | Low risk | Low risk | Low risk | Low risk | Low risk | Unclear risk |
| Vilsbøll, 2007(383) | | Low risk | Low risk | Low risk | Low risk | Unclear risk | Low risk | Low risk |
| Seino, 2008(384) | | Low risk | Low risk | Low risk | Low risk | Low risk | Low risk | Low risk |
| Vilsbøll, 2008(385) | | Low risk | Low risk | Low risk | Low risk | Low risk | Low risk | Low risk |
| Miyagawa, 2015(386) | | Low risk | Low risk | Low risk | Low risk | Low risk | Low risk | Low risk |
| Moretto, 2008(387) | | Low risk | Low risk | Low risk | Low risk | Low risk | Low risk | Low risk |
| Rosenstock, 2009(388) | | Low risk | Low risk | Low risk | Low risk | Low risk | Low risk | Low risk |
| Seino,2014(389) | | Low risk | Low risk | Low risk | Low risk | Unclear risk | Low risk | Low risk |
| Nauck, 2016(390) | | Low risk | Low risk | Low risk | Low risk | Low risk | Low risk | Low risk |
| Grunberger, 2012(391) | | Low risk | Low risk | Low risk | Low risk | Low risk | Low risk | Low risk |
| Terauchi, 2014(392) | | Low risk | Low risk | Low risk | Low risk | Low risk | Low risk | Low risk |
| Fonseca, 2012(393) | | Low risk | Low risk | Low risk | Unclear risk | Low risk | Low risk | Low risk |
| Raz, 2012(394) | | Low risk | Low risk | Low risk | Low risk | Low risk | Low risk | Low risk |
| Hollander, 2013(395) | | Low risk | Low risk | Low risk | Low risk | Low risk | Low risk | Low risk |
| **Augmented PBO effect by injection** | | | | | | | | |
| **GLP-1RA vs PBO** | |  |  |  |  |  |  |  |
| Ambery, 2018(396) | | Low risk | Low risk | Low risk | Low risk | Low risk | Low risk | Low risk |
| Idorn, 2016(397) | | Low risk | Low risk | Low risk | Low risk | Low risk | Low risk | Low risk |
| Tonneijck, 2016(398) | | Low risk | Low risk | Low risk | Low risk | Low risk | Low risk | Low risk |
| Scholten,2017 (399) | | Low risk | Low risk | Low risk | Low risk | Low risk | Low risk | Low risk |
| Gao, 2009(400) | | Unclear risk | Unclear risk | Low risk | Low risk | Low risk | Low risk | Unclear risk |
| Kadowaki, 2009(401) | | Low risk | Low risk | Low risk | Low risk | Low risk | Low risk | Low risk |
| Gill, 2010(402) | | Low risk | Low risk | Low risk | Low risk | Low risk | Low risk | Low risk |
| Nauck, 2016(403) | | Low risk | Low risk | Low risk | Low risk | Low risk | Low risk | Low risk |
| Ratner, 2010(404) | | Low risk | Low risk | Low risk | Low risk | Unclear risk | Low risk | Low risk |
| Kim, 2007(405) | | Unclear risk | Unclear risk | Low risk | Low risk | Low risk | Low risk | Low risk |
| Umpierrez, 2011(406) | | Low risk | Low risk | Low risk | Low risk | Low risk | Low risk | Low risk |
| Kaku, 2010(407) | | Low risk | Low risk | Low risk | Low risk | Unclear risk | Low risk | Low risk |
| Lind, 2015(408) | | Low risk | Low risk | Low risk | Low risk | Low risk | Low risk | Low risk |
| Apovian, 2010(409) | | Low risk | Low risk | Low risk | Low risk | Low risk | Low risk | Low risk |
| Ludvik, 2018(410) | | Low risk | Low risk | Low risk | Low risk | Low risk | Low risk | Low risk |
| Dungan, 2016(411) | | Low risk | Low risk | Low risk | Low risk | Low risk | Low risk | Low risk |
| Seino, 2012(412) | | Low risk | Low risk | Low risk | Low risk | Low risk | Low risk | Low risk |
| Ahrén, 2013(413) | | Low risk | Low risk | Low risk | Low risk | Low risk | Low risk | Low risk |
| Riddle, 2013-1(414) | | Low risk | Low risk | Low risk | Low risk | Low risk | Low risk | Low risk |
| Riddle, 2013-2(415) | | Low risk | Low risk | Low risk | Low risk | Low risk | Low risk | Low risk |
| Bolli, 2014(416) | | Low risk | Low risk | Low risk | Low risk | Low risk | Low risk | Low risk |
| Pan, 2014(417) | | Low risk | Low risk | Low risk | Low risk | Low risk | Low risk | Unclear risk |
| Rosenstock, 2014(418) | | Low risk | Low risk | Low risk | Low risk | Low risk | Low risk | Low risk |
| Meneilly, 2017(419) | | Low risk | Low risk | Low risk | Low risk | Low risk | Low risk | Low risk |
| Yang, 2017(420) | | Low risk | Low risk | Low risk | Low risk | Low risk | Low risk | Low risk |
| Bergenstal, 2012(302) | | Unclear risk | Unclear risk | Low risk | Low risk | Low risk | Low risk | Low risk |
| Henry, 2012(421) | | Low risk | Low risk | Low risk | Low risk | Low risk | Low risk | Low risk |
| Vanderheiden, 2016(422) | | Low risk | Low risk | Low risk | Low risk | Low risk | Low risk | Low risk |
| Frias, 2018(423) | | Low risk | Low risk | Low risk | Low risk | Low risk | Low risk | Low risk |
| Marre, 2009(257) | | Low risk | Low risk | Low risk | Low risk | Low risk | Low risk | Low risk |
| Nauck, 2009(206) | | Low risk | Low risk | Unclear risk | Low risk | Low risk | Low risk | Low risk |
| Russell-Jones, 2009(424) | | Low risk | Low risk | Low risk | Unclear risk | Low risk | Low risk | Unclear risk |
| Zinman, 2009(425) | | Low risk | Low risk | Low risk | Low risk | Low risk | Low risk | Low risk |
| Ahmann, 2015(426) | | Unclear risk | Unclear risk | Low risk | Low risk | Low risk | Low risk | Low risk |
| Davies, 2016(427) | | Low risk | Low risk | Low risk | Low risk | Low risk | Low risk | Low risk |
| Lingvay, 2018(428) | | Low risk | Low risk | Low risk | Low risk | Low risk | Low risk | Low risk |
| Zinman, 2007(429) | | Low risk | Low risk | Low risk | Low risk | Low risk | Low risk | Low risk |
| Liutkus, 2010(430) | | Low risk | Low risk | Low risk | Low risk | Low risk | Low risk | Low risk |
| Wysham, 2014(431) | | Low risk | Low risk | Low risk | Low risk | Low risk | Low risk | Low risk |
| Guja, 2018(432) | | Low risk | Low risk | Low risk | Low risk | Low risk | Low risk | Unclear risk |
| Pozzilli, 2017(433) | | Low risk | Low risk | Unclear risk | Low risk | Low risk | Low risk | Low risk |
| Buse, 2011(434) | | Low risk | Low risk | Low risk | Low risk | Low risk | Low risk | Low risk |
| Buse, 2014(435) | | Low risk | Low risk | Unclear risk | Low risk | Low risk | Low risk | Unclear risk |
| Buse, 2004(436) | | Low risk | Low risk | Low risk | Low risk | Low risk | Low risk | Low risk |
| Defronzo, 2005(437) | | Low risk | Low risk | Low risk | Low risk | Low risk | Low risk | Low risk |
| Kendall, 2005(438) | | Low risk | Low risk | Low risk | Low risk | High risk | Low risk | Unclear risk |
| Rodbard, 2018(439) | | Low risk | Low risk | Low risk | Low risk | Low risk | Low risk | Low risk |
| Seino, 2016(440) | | Low risk | Low risk | Low risk | Low risk | Low risk | Low risk | Low risk |
| Reusch, 2014(441) | | Low risk | Low risk | Low risk | Low risk | Low risk | Low risk | Low risk |
| Weinstock, 2014(442) | | Low risk | Low risk | Low risk | Low risk | Low risk | Low risk | Low risk |
| Davies, 2015(443) | | Low risk | Low risk | Low risk | Low risk | Low risk | Low risk | Low risk |
| Pinget, 2013(444) | | Low risk | Low risk | Low risk | Low risk | Low risk | Low risk | Low risk |
| Hernandez, 2018(445) | | Low risk | Low risk | Low risk | Low risk | Low risk | Low risk | Unclear risk |
| Ahrén, 2014(446) | | Low risk | Low risk | Low risk | Low risk | Low risk | Low risk | Low risk |
| Marso, 2016(447) | | Low risk | Low risk | Low risk | Low risk | Low risk | Low risk | Low risk |
| Home, 2015(448) | | Low risk | Low risk | Low risk | Low risk | Low risk | Low risk | Low risk |
| Holman, 2017 (449) | | Low risk | Low risk | Low risk | Low risk | Low risk | Low risk | Low risk |
| Marso, 2016 (450) | | Low risk | Low risk | Low risk | Low risk | Low risk | Low risk | Low risk |

**Table S5. Baseline characteristics by placebo response with HbA1c and weight change**

| Variables | HbA1c change | | | Weight change | | |
| --- | --- | --- | --- | --- | --- | --- |
|  | HbA1c reduction | HbA1c elevation | P value | Weight reduction | Weight elevation | P value |
| Analysis for type 1 diabetes | | | | | | |
| Age (year) | 41.10 (36.15, 43.78) | 39.70 (16.00, 42.00) | 0.302 | 41.30 (37.55, 48.18) | 41.00 (16.9, 42.70) | 0.366 |
| Male percentage (%) | 51.00 (45.50, 57.50) | 50.00 (39.60, 54.25) | 0.404 | 53.00 (46.00, 53.00) | 49.00 (44.38, 56.50) | 0.708 |
| Duration of DM (year) | 18.35 (14.80, 21.05) | 15.75 (6.38, 23.48) | 0.542 | 18.20 (18.20, 20.70) | 18.75 (18.20, 20.70) | 0.757 |
| Study duration (week) | 24 (12, 48) | 26 (24, 36) | 0.276 | 39 (24, 52) | 24 (12, 36) | 0.100 |
| Baseline HbA1c (%) | 8.38 (8.01, 8.98) | 8.00 (7.79, 8.80) | 0.262 | 8.56 (7.77, 9.00) | 8.23 (7.90, 8.80) | 0.754 |
| Baseline BMI (kg/m^2^) | 26.35 (24.11, 28.08) | 27.00 (25.30, 28.00) | 0.833 | 26.50 (25.18, 28.38) | 27.15 (24.60, 28.00) | 0.939 |
| Publication year | 2013 (2004, 2016) | 2015 (2013, 2017) | 0.391 | 2005 (2004, 2016) | 2015 (2006, 2017) | 0.181 |
| Analysis for type 2 diabetes | | | | | | |
| Age (year) | 56.60 (54.60, 58.75) | 57.30 (55.00, 59.88) | **0.044** | 56.50 (54.70, 58.60) | 57.00 (55.00, 59.78) | 0.225 |
| Male percentage (%) | 54.40 (48.00, 61.00) | 59.20 (50.38, 66.70) | **0.001** | 55.70 (48.10, 62.00) | 57.70 (49.60, 66.00) | 0.202 |
| Duration of DM (year) | 7.10 (5.19, 9.80) | 6.30 (4.62, 8.50) | 0.109 | 6.40 (5.00, 8.60) | 7.30 (4.80, 10.00) | **<0.001** |
| Study duration (week) | 24 (16, 26) | 24 (14, 26) | 0.315 | 24 (16, 26) | 24 (16, 28) | **0.025** |
| Baseline HbA1c (%) | 8.37 (7.97, 8.70) | 8.11 (7.90, 8.60) | **0.027** | 8.09 (7.84, 8.40) | 8.34 (7.99, 8.70) | **0.001** |
| Baseline BMI (kg/m^2^) | 31.05 (28.20, 32.48) | 30.00 (27.80, 31.60) | **0.002** | 30.70 (27.83, 32.20) | 31.70 (28.20, 32.00) | **0.040** |
| Publication year | 2012 (2008, 2015) | 2007 (2001, 2013) | **<0.001** | 2012 (2008, 2015) | 2009 (2001, 2013) | **0.001** |

Baseline characteristics were expressed as median with interquartile range. Font bold was used if P<0.05 was noticed. BMI, body mass index; DM, diabetes, mellitus.

**Figure S1. Flowchart of the study**


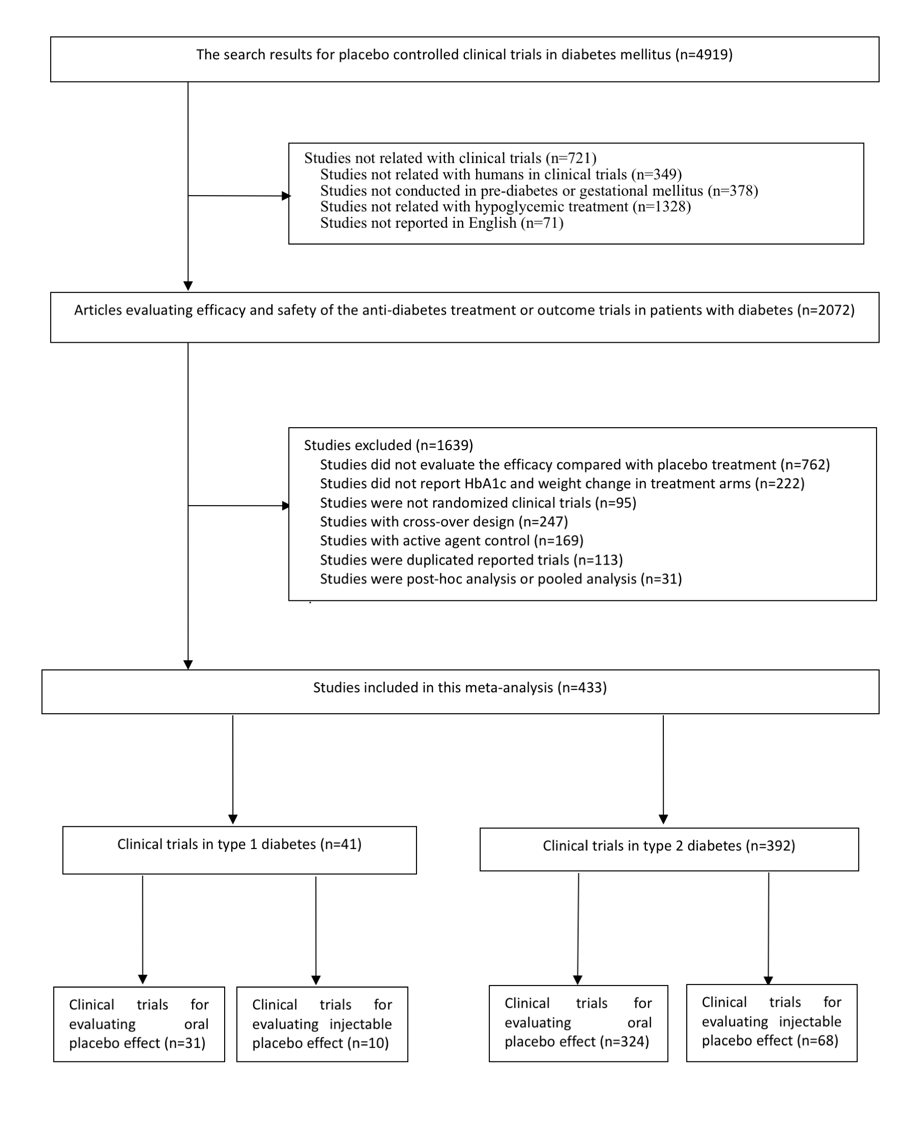


**Figure S2. Egger’s test for trials with type 1 diabetes**


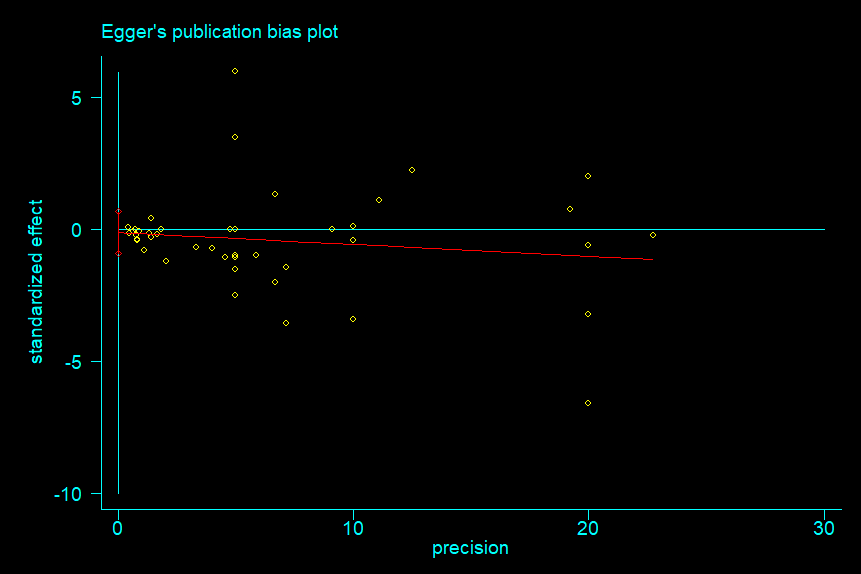


**Figure S3. Egger’s test for trials with type 2 diabetes**


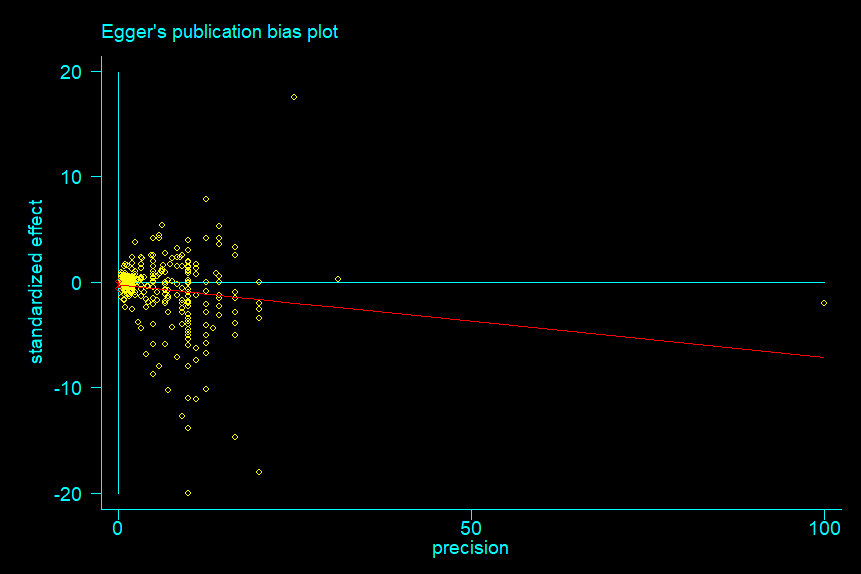


**Figure S4. Placebo response stratified by patient age in type 1 diabetes mellitus**

**
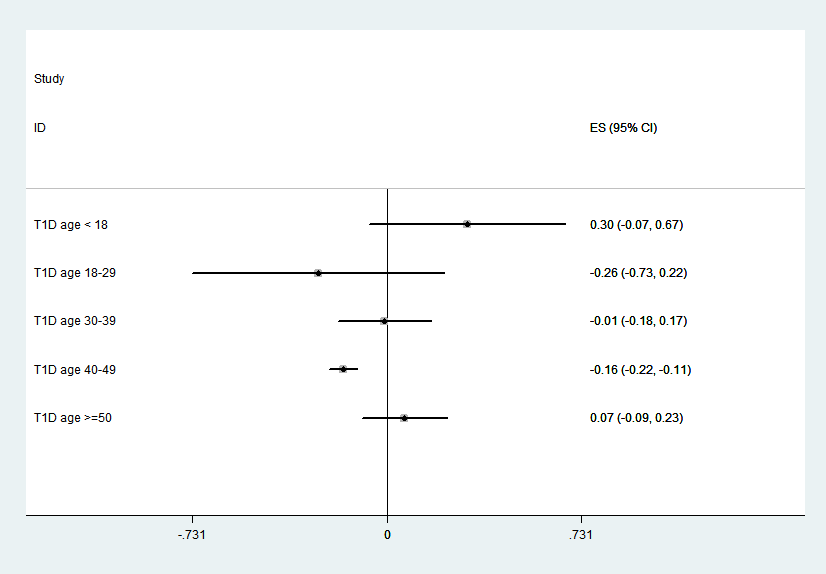

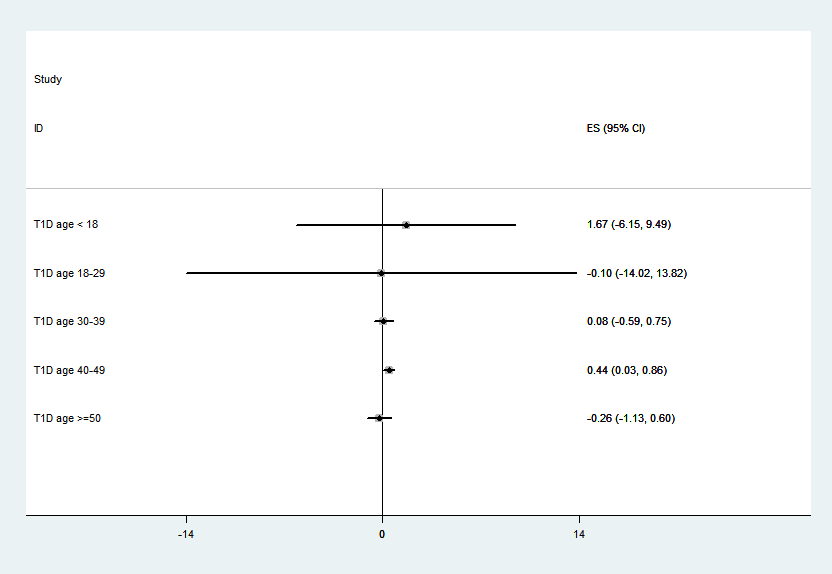
**

1. **HbA1c change (b) Weight change**

**Figure S5. Placebo response stratified by patient age in type 2 diabetes mellitus**

**
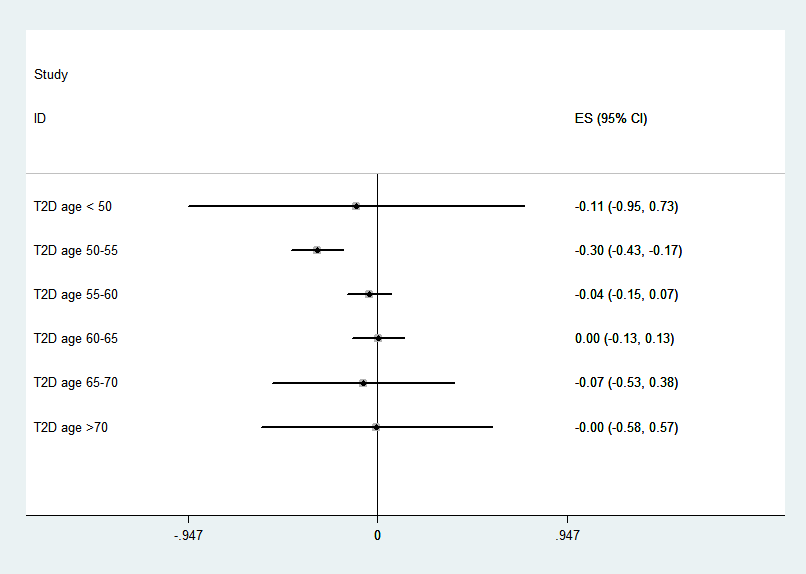

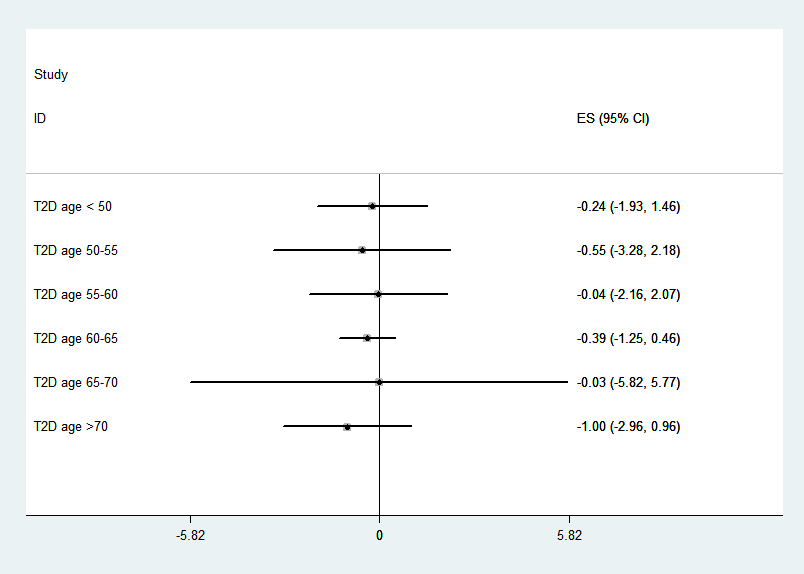
**

1. **HbA1c change (b) Weight change**

**Figure S6. Placebo response stratified by male percentage in type 2 diabetes mellitus**

**
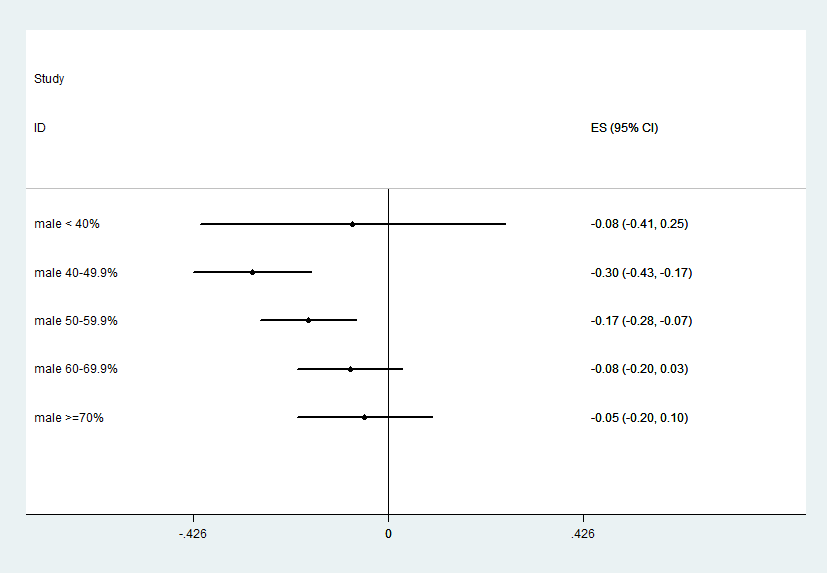

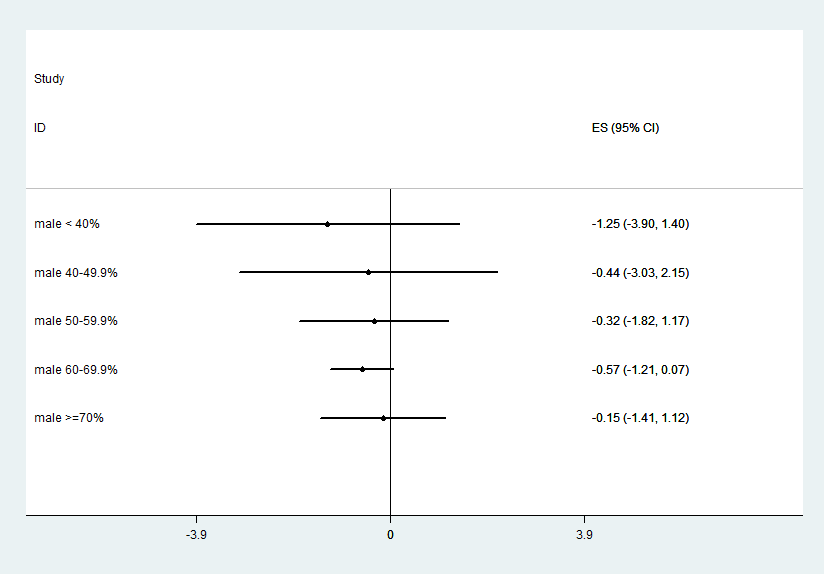
**

1. **HbA1c change (b) Weight change**

**Figure S7. Placebo response stratified by male percentage in type 1 diabetes mellitus**

**
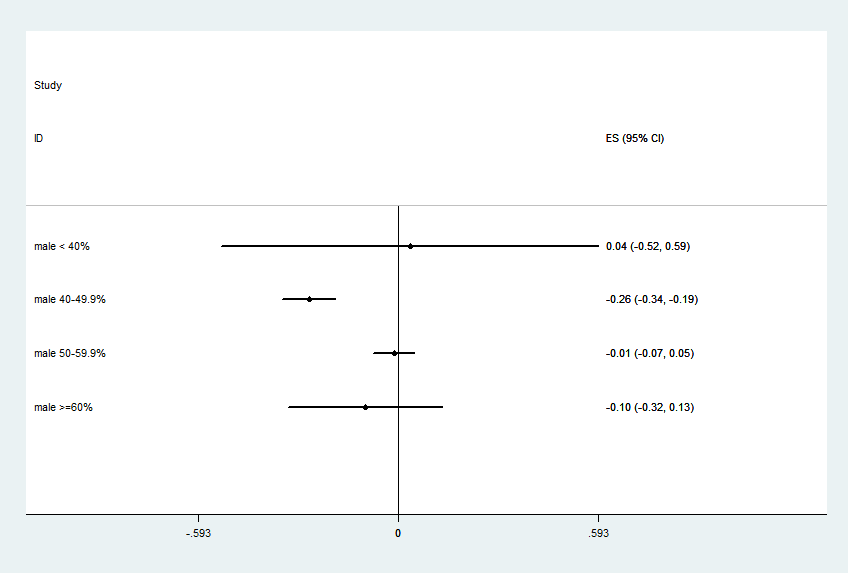

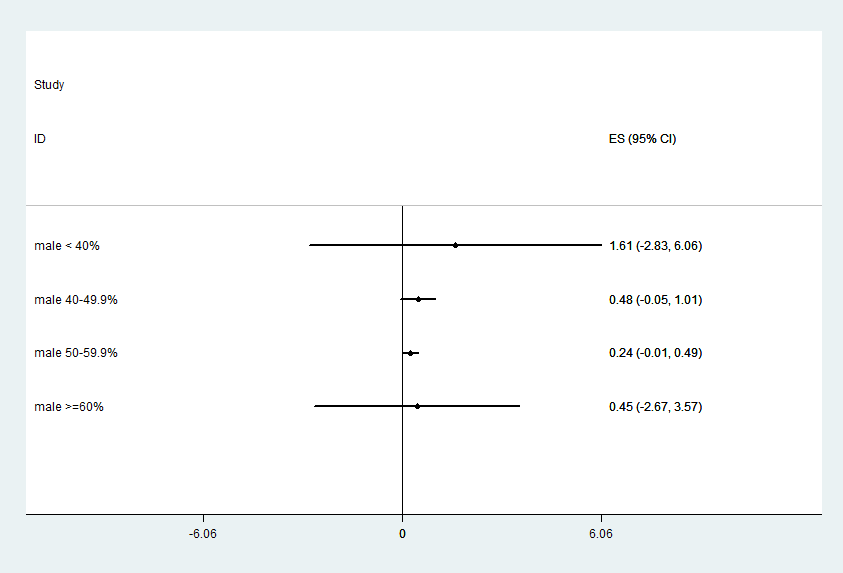
**

1. **HbA1c change (b) Weight change**

**Figure S8. Placebo response stratified by baseline BMI in type 2 diabetes mellitus**

**
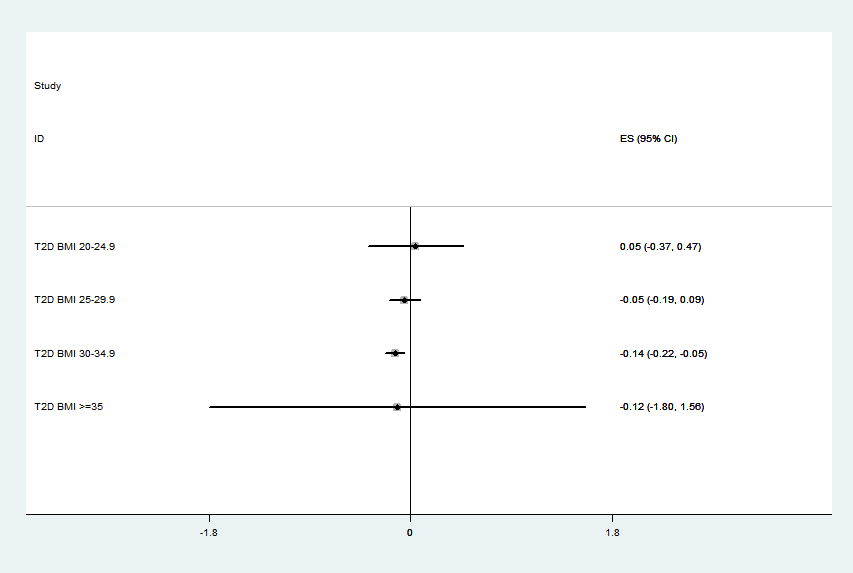

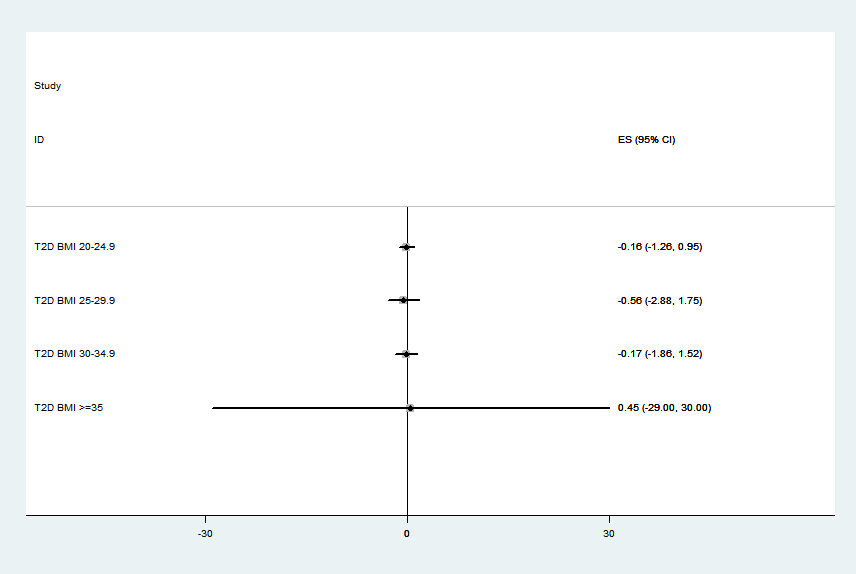
**

1. **HbA1c change (b) Weight change**

**Figure S9. Placebo response stratified by baseline BMI in type 1 diabetes mellitus**

**
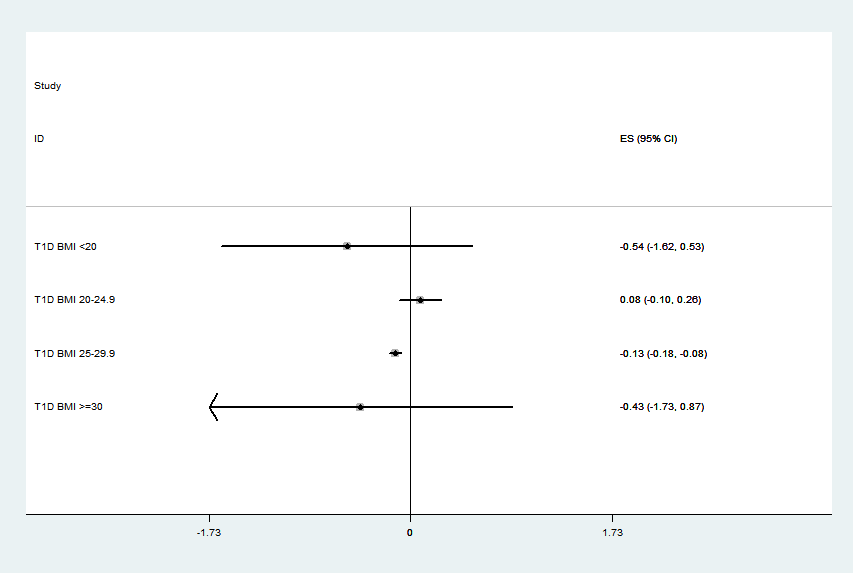

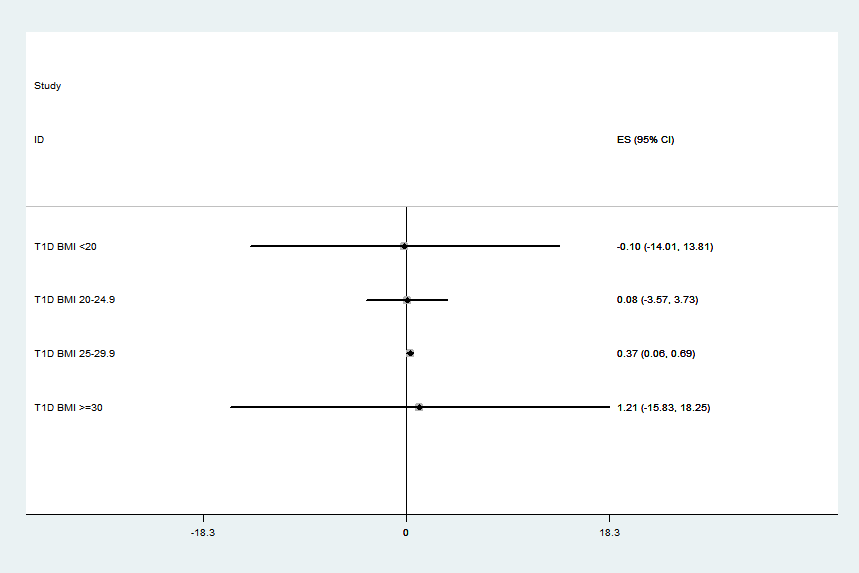
**

1. **HbA1c change (b) Weight change**

**Figure S10. Placebo response stratified by baseline HbA1c in type 2 diabetes mellitus**

**
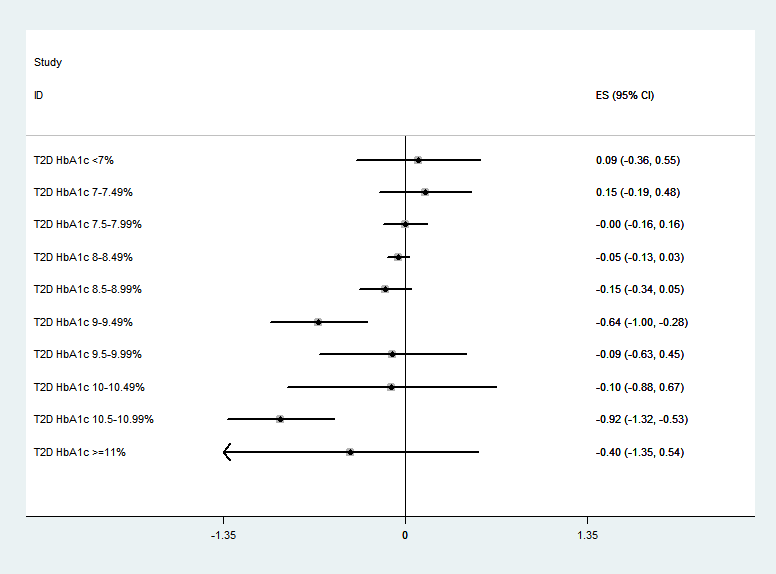

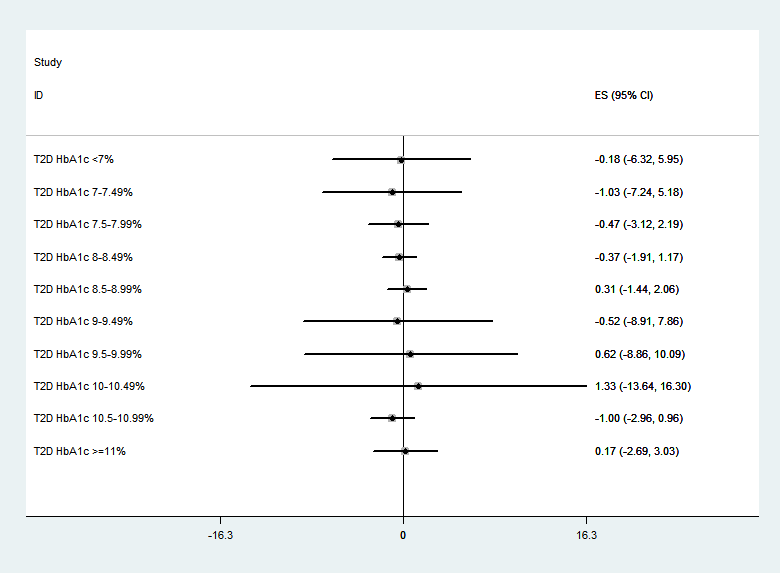
**

1. **HbA1c change (b) Weight change**

**Figure S11. Placebo response stratified by baseline HbA1c in type 1 diabetes mellitus**

**
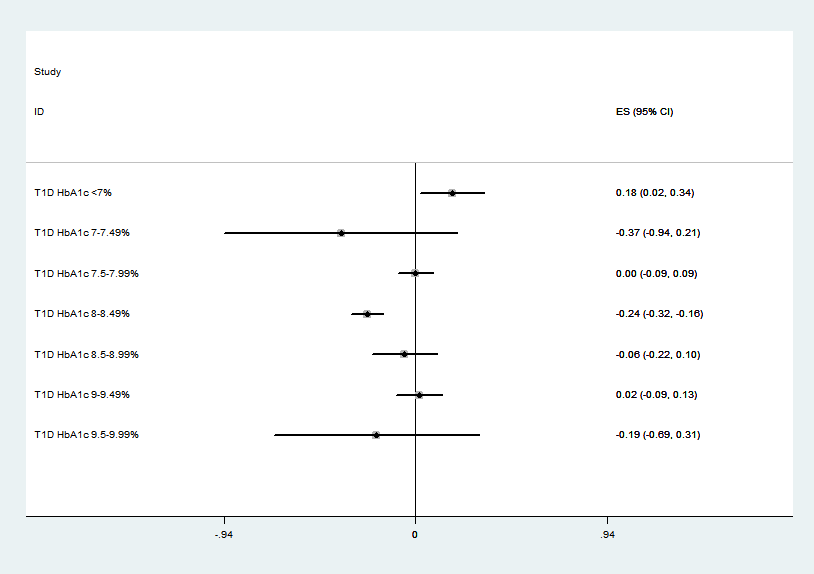

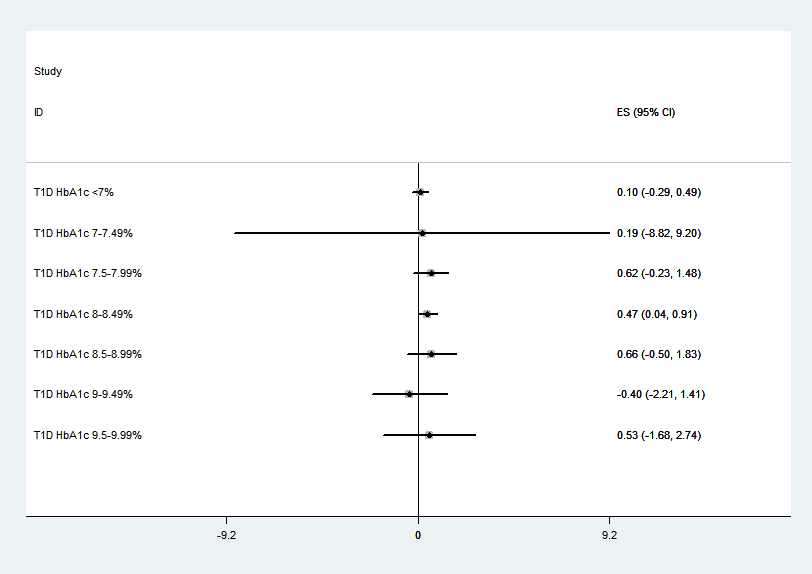
**

**Figure S12. Placebo response stratified by disease duration in type 1 diabetes mellitus**

**
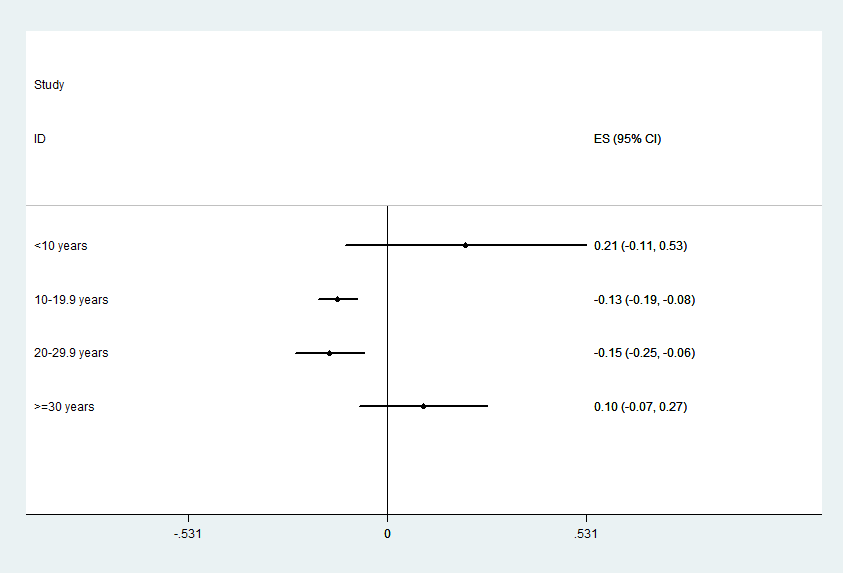

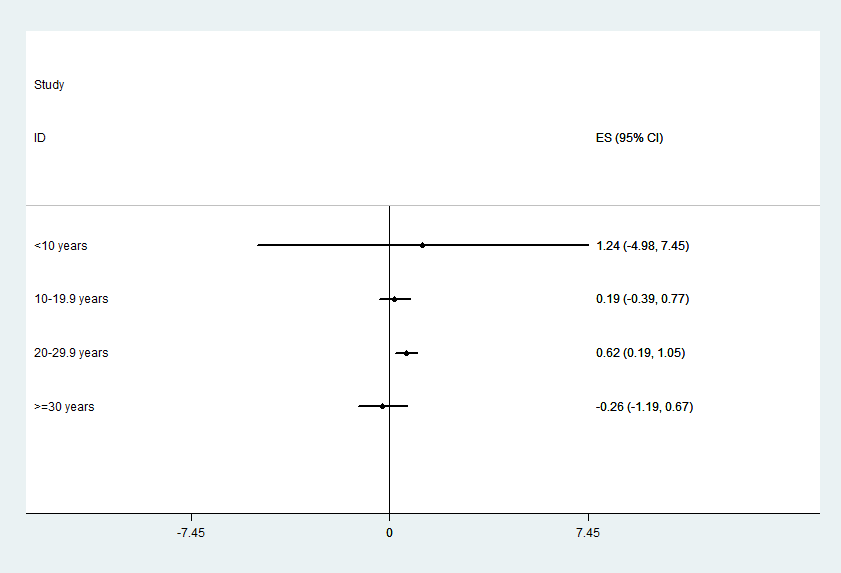
**

1. **HbA1c change (b) Weight change**

**Figure S13. Placebo response stratified by disease duration in type 2 diabetes mellitus**

**
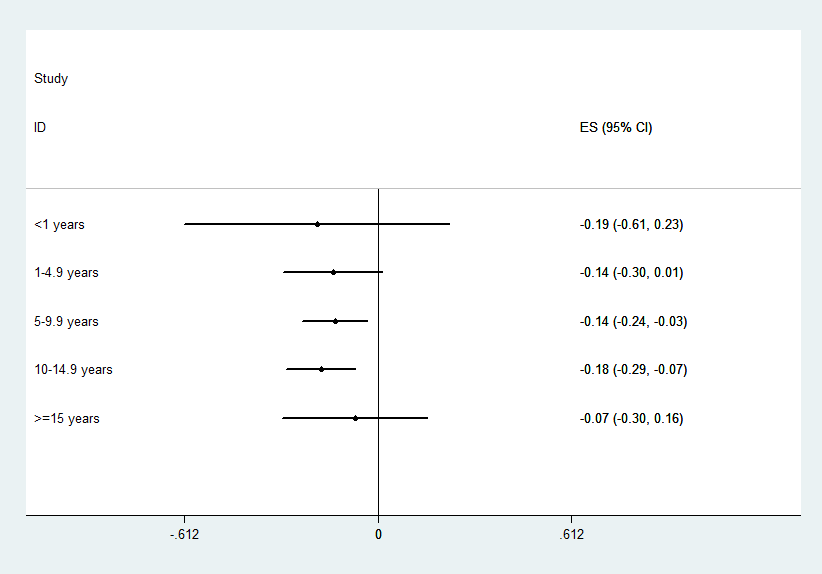

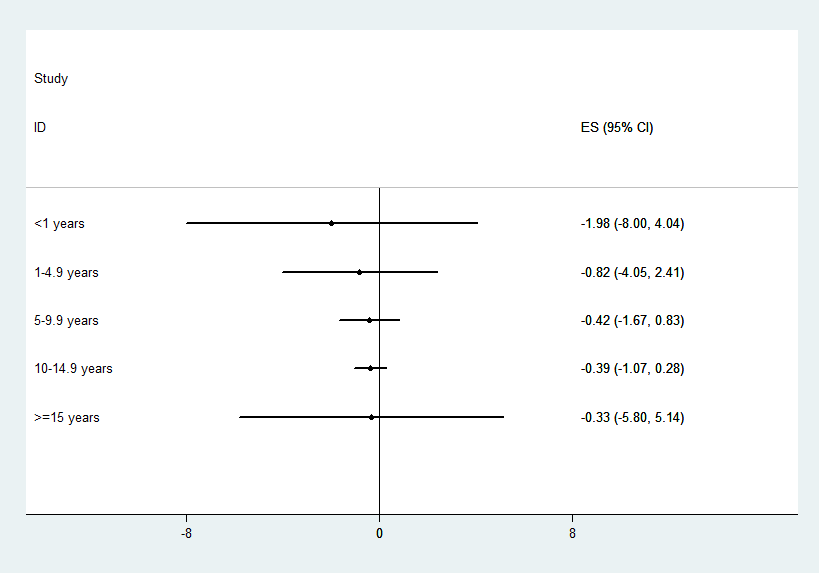
**

1. **HbA1c change (b) Weight change**

**Figure S14. Placebo response stratified by study duration in type 1 diabetes mellitus**

**
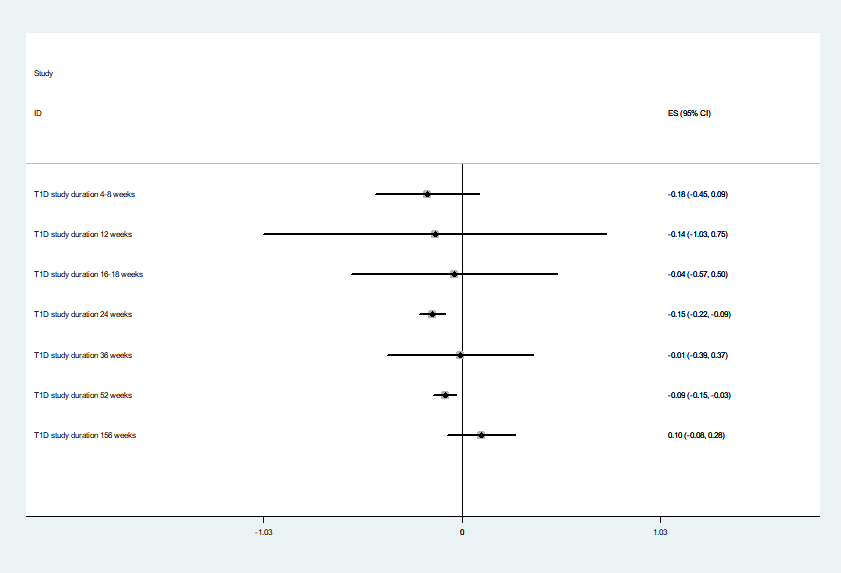

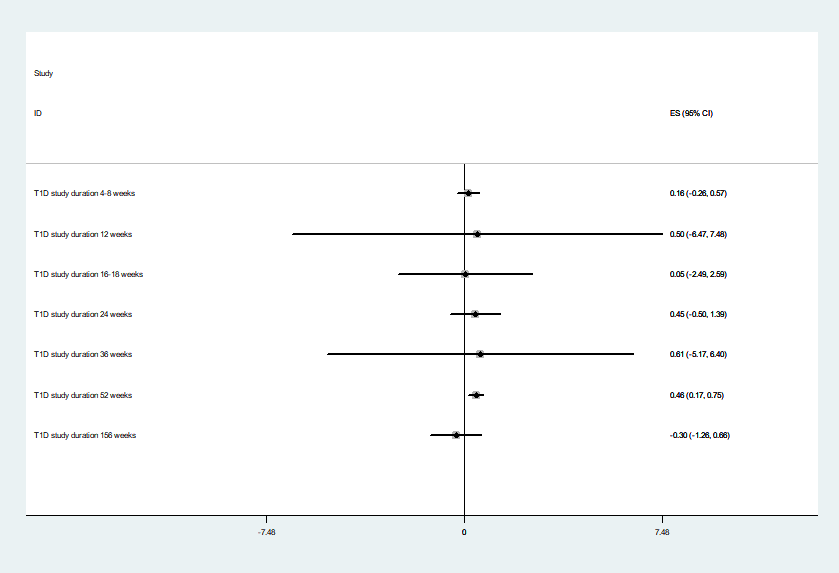
**

1. **HbA1c change (b) Weight change**

**Figure S15. Placebo response stratified by study duration in type 2 diabetes mellitus**

**
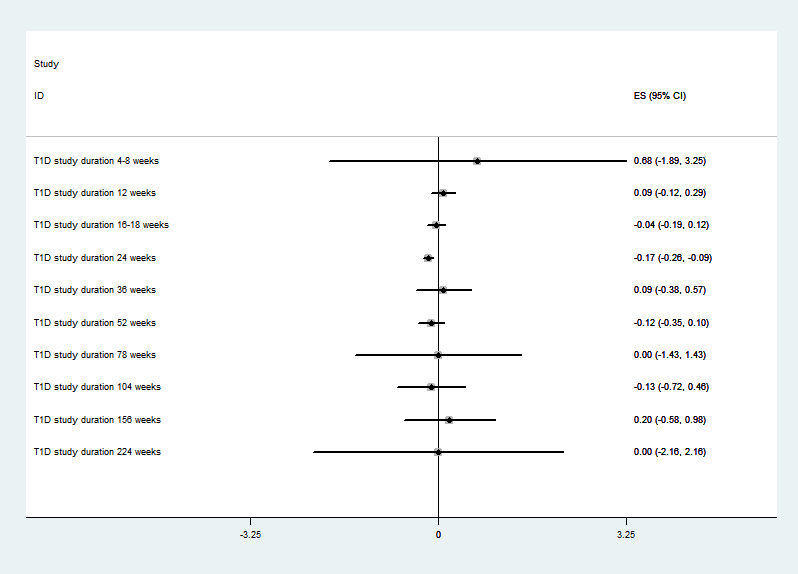

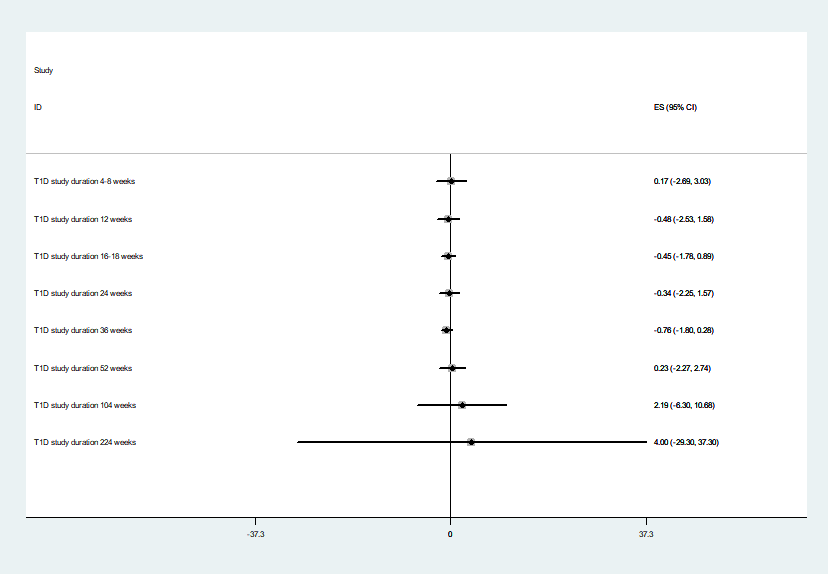
**

1. **HbA1c change (b) Weight change**

**Figure S16. Placebo response stratified by publication year in type 1 diabetes mellitus**

**
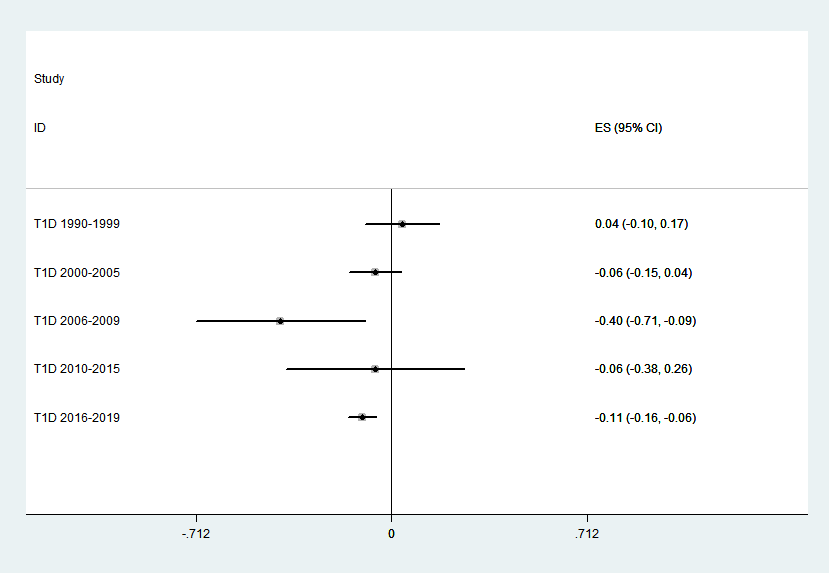

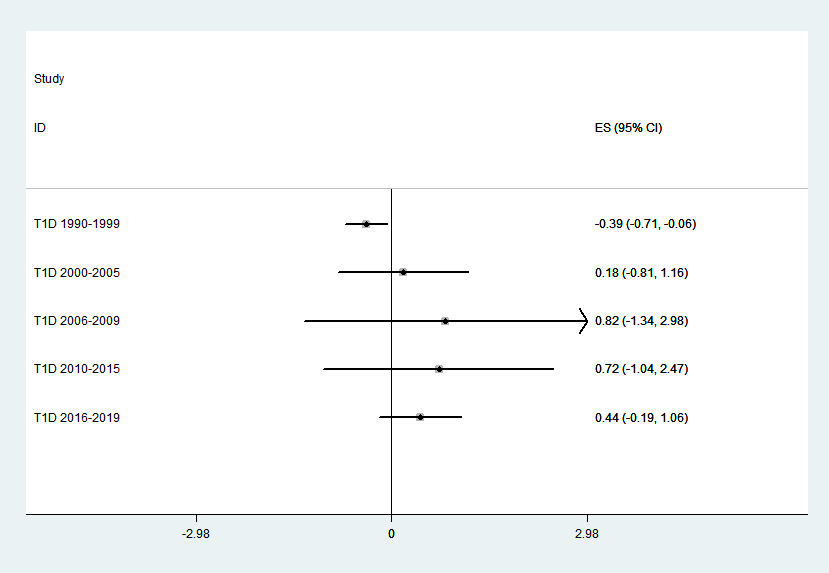
**

1. **HbA1c change (b) Weight change**

**Figure S17. Placebo response stratified by publication year in type 2 diabetes mellitus**

**
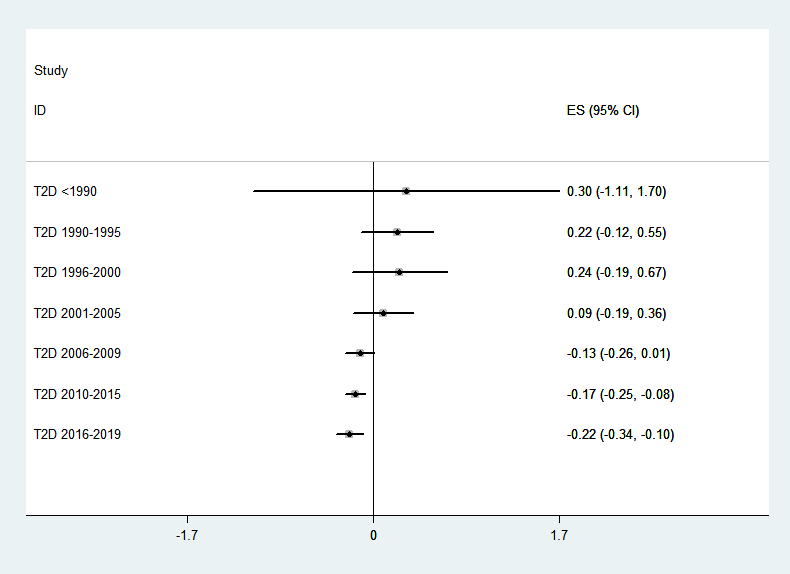

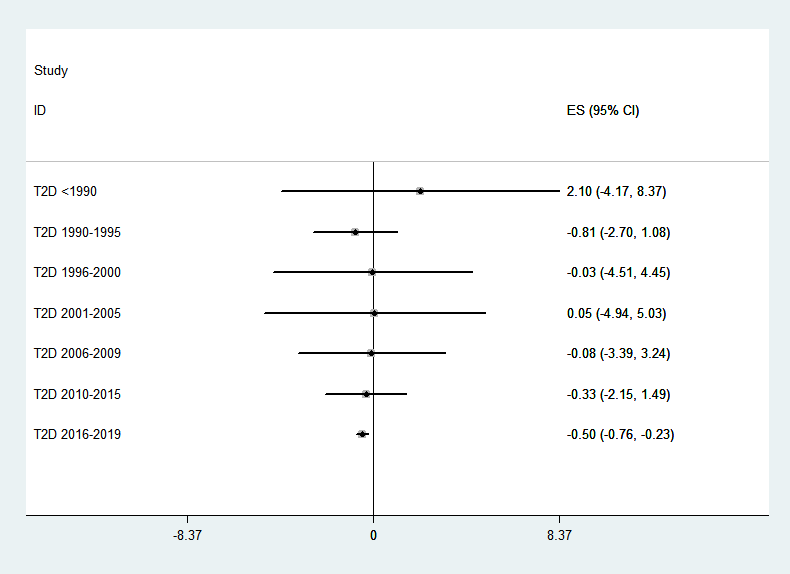
**

1. **HbA1c change (b) Weight change**
